# Global biogeography of living brachiopods: Bioregionalization patterns and possible controls

**DOI:** 10.1371/journal.pone.0259004

**Published:** 2021-11-08

**Authors:** Facheng Ye, G. R. Shi, Maria Aleksandra Bitner

**Affiliations:** 1 School of Earth, Atmospheric and Life Sciences, Faculty of Science, Medicine and Health, University of Wollongong, Wollongong, New South Wales, Australia; 2 Department of Evolutionary Paleobiology, Institute of Paleobiology, Polish Academy of Sciences, Warsaw, Poland; University of California, UNITED STATES

## Abstract

The global distribution patterns of 14918 geo-referenced occurrences from 394 living brachiopod species were mapped in 5° grid cells, which enabled the visualization and delineation of distinct bioregions and biodiversity hotspots. Further investigation using cluster and network analyses allowed us to propose the first systematically and quantitatively recognized global bioregionalization framework for living brachiopods, consisting of five bioregions and thirteen bioprovinces. No single environmental or ecological variable is accountable for the newly proposed global bioregionalization patterns of living brachiopods. Instead, the combined effects of large-scale ocean gyres, climatic zonation as well as some geohistorical factors (e.g., formation of land bridges and geological recent closure of ancient seaways) are considered as the main drivers at the global scale. At the regional scale, however, the faunal composition, diversity and biogeographical differentiation appear to be mainly controlled by seawater temperature variation, regional ocean currents and coastal upwelling systems.

## Introduction

As a phylum, brachiopods are a common and important component of the Phanerozoic fossil record; they were the dominant and essential elements in the marine ecosystems throughout the Palaeozoic Era (542~252 million years ago/Ma). Although their global abundance and ecological role were abruptly diminished by the end-Permian global mass extinction *ca* 252 Ma, representatives of this phylum persisted through the Mesozoic and Cenozoic till the present time, still with approximately 400 species in approximately 100 genera surviving today.

Presumably due to their sheer relative abundance, distributional prevalence and ecological dominance, the palaeobiogeography of fossil brachiopods has been studied extensively [e.g., 1–6]. In contrast, however, the biogeography of living brachiopods has received only limited attention (Table 1). To our knowledge, in the past 110 years since the publication of Schuchert’s [7], pioneering study on the global provincial patterns of living brachiopods, there have been no more than 20 studies on the global biogeography of living brachiopods. The focus and significance of these early studies could be summarized in three broad areas. First, there appears to be a consistent recognition in support of a degree of global provincialism within living brachiopods [7–15], although the data and methodology used in these early investigations and the provincial patterns recognized differed significantly among these studies. Second, there has been an increasing recognition and confirmation that the global biodiversity distribution of living brachiopods is conspicuously asymmetrical with a peak of diversity in the mid latitudes of both hemispheres [10, 12, 13, 15–18], in stark contrast to the commonly accepted bell-shaped latitudinal biodiversity gradient (LBG) model perceived for most organisms [e.g., 19]. A third significant area of progress made in previous research on the global biogeography of living brachiopods is the appreciation that there exist strong bathymetrical and oceanographic (hydrographic) controls on the distribution of brachiopod species in world oceans resulting in distinct depth-related zonation patterns in species richness [12–15, 20, 21] or body size [22].

**Table 1:** Summary of key references on the biogeography, bioregionalization of living brachiopods.

Reference	Analysed taxa	Region	Methodology and hierarchical pattern for bioregionalization	Remarks
Schuchert [7]	166 (158 established) species in 33 genera	Global, but discussed separately based on regions	Based on distribution of brachiopod genera, 1 realm and 4 regions	Most brachiopods live in shallow water and of continental or epicontinental seas, 5 great brachiopod regions have identified
Elliott [8]	Terebratellides in 17 genera	Global	Grouped terebratelloid brachiopods into different distributional classes, 3 distributional groups	Distributional classes: worldwide and northern/southern hemisphere groups have identified
Rudwick [16]		Global		Varied asymmetric latitudinal diversity curves in different brachiopod groups with peaks at tropical-subtropical and temperate zones
Zezina [9]	279 species	Global	Distribution of brachiopods, 5 groups with 19 ranges	5 main and 19 basic geographical elements of fauna distinguished
Emig [20]	340 described species	Global		Peak bathymetrical distribution between 50 to 400m
Walsh [17]	Articulated brachiopod	Pacific and Atlantic regions		Pacific: Asymmetric bimodal latitudinal diversity curve with peak zones at 30°~40°S/N
				Atlantic: Asymmetric bimodal latitudinal diversity curve with peak zones at mid-latitudes in Northern Hemisphere, and 30°~40°S
Emig [10]	Inarticulate brachiopod	Global	Based on distribution of different families, 3 families correspond to 3 different distribution areas	Variable latitudinal distribution in different families, a peak diversity zone occurs around 20°N when consider all inarticulated brachiopods
Richardson [11]	Articulated brachiopod	Global	Based on distribution of different families, 3 regional patters	Detail distribution of different articulated brachiopod families. In addition to Austral and boreal families, regional patterns: southern/northern areas, northern Pacific region have also been identified
Zezina [12]		Global		Identified seven (<700m) and three (>700m) latitudinal fauna area belts, asymmetrical features of faunistic arrangement along equator and meridian
Zezina [13]		Global		Eutrophication cause by constant upwelling plays a critical impact on living brachiopod asymmetry distribution phenomenon between West and East of oceans
Logan [14]	Articulated brachiopod with 336 species in 100 genera	Global		Geographical distribution plotted on the world map. 46% species found only within 200 m depth
Zezina [15]	370 species in 116 genera	Global	Based on similarity in their distribution, 24 groups (geographical elements)	Summary on vertical and latitudinal and circum-continental distribution patterns of living brachiopod, and revealed the govern effect by global hydrological condition and dynamical evolution change
Powell [18]	Database with 4394 fossil and recent brachiopod genera	Global		Asymmetric bimodal latitudinal diversity curve with a peak at 30°N ~40°N
Peck and Harper [22]	71 genera of terebratulide, 15 genera of rhynchonellide and 4 genera of thecideid	Global		In Terebratulide, length of shell increase as the latitude increases and decrease as depth increase.
Álvarez et al. [21]		Atlantic ocean and Mediterranean sea		Peak diversity at nearly 100m with high density

Notwithstanding the significance of these early studies in providing an excellent foundation for the modern understanding of the global distribution patterns of living brachiopods, they all have suffered from the lack of a robust quantitative approach applied to a comprehensive global database inclusive of all verified species and occurrence records. This deficiency may explain why, until now, there is yet no unified framework depicting and characterizing the global biogeography of living brachiopods in the form of bioregions, hierarchy, regional endemism and mutual distinctions. This is in sharp contrast to living marine bivalves—a group of marine invertebrates that are often considered to have replaced the role of brachiopods as the ecological dominants in the marine ecosystems since the end-Permian mass extinction [e.g., 23, but see 24 for a different view]—whose global biogeography has been extensively studied and documented [e.g., 25–28].

To advance the research of living brachiopod biogeography and to build on existing knowledge, the present project was designed to serve three purposes. First, we attempted to build the first global database of the spatial distribution of living brachiopods, by conducting a thorough and detailed review and taxonomic evaluation of all known and published living brachiopod species and their occurrence records (S1 Table). Second, following the establishment of the brachiopod distributional database, we then conducted a comprehensive analysis on the biogeography of living brachiopods through quantitative analyses (cluster analysis and network analysis), from which the global bioregionalization patterns of living brachiopods were revealed and mapped. Finally, by comparing the recognized global brachiopod bioregionalization patterns with the distribution of world major ocean currents, climatic zones, upwelling systems and land barriers created by recent geological events, we evaluated how each of these factors might have contributed to the present-day distributional patterns of living brachiopods.

This paper is the first of a sequence of studies designed to investigate the ecological biogeography of global living brachiopods, in an attempt to pursue a deeper understanding of the global distributional patterns and underlying control mechanisms of this substantially under-studied phylum. In at least two subsequent papers, we plan to explore (1) the relationships among brachiopod species richness, latitudes, latitudinal ranges, shelf area, and latitude-bound diversification rates; and (2) how the body size of living brachiopods have responded to water depth, latitudes, substrate type, latitude-bound calcification rate, and geological age. The implications expected to derive from these studies are important not only for providing an independent source of data and study model to test some of the enduring global biogeographical theories, they are also expected to add significant intellectual benefits to palaeobiology and palaeobiogeography by demonstrating the fact the distribution of brachiopods could be used as a crucial tool to reconstruct the geological history of large-scale geographical, oceanographical, climatic and biotic changes in the Phanerozoic, especially for the Palaeozoic Era when brachiopods were the dominant invertebrates of the ocean ecosystems.

## Materials and methods

### Sampling and data acquisition

A database of living brachiopod occurrences (records) was first compiled from available literature (references list is available from the online S2 Table), GBIF (Global Biodiversity Information Facility, https://www.gbif.org/) and OBIS (Ocean Biogeographic Information System, http://www.iobis.org/). This raw database contains 19203 records, and most of occurrence record is featured by the brachiopod’s original genus name, species name, and details of the specific collection locality, which includes the geographical name of the locality and its precise latitude and longitude geo-information.

To optimize the database suitable for a global quantitative biogeographical analysis, a deliberate ‘data cleaning’ effort was undertaken to reduce data dimensionality and the impacts of potential data noise inherently associated with multidimensional raw datasets. In performing this task, we first removed any taxonomic records with syntaxes like aff., cf., and indet., but retained species with syntaxes sp. and spp. for genus-level analysis. Taxonomic misspellings were also carefully checked and reconciled (S1 Table). Subsequently, the updated species names and synonyms were standardized according to the BrachNet (http://paleopolis.rediris.es/BrachNet/), and WoRMS (World Register of Marine Species, http://www.marinespecies.org/), then carefully checked and confirmed by one of us (M. A. Bitner) who has studied living brachiopods for many years.

Following on from the above steps, we then manually checked the location details of each occurrence record. More than 80% of the occurrence records in our raw database have detailed latitude and longitude geo-information. All geographical data were converted to a Decimal Degrees system. For the records that only have provided location names (e.g., Macquarie Island, Cook Strait, Cape Finisterre) without specific latitudes and longitudes, we manually matched and entered their geographical coordinates through Google Maps accurate to 0.5° latitude/longitude resolution. Some obvious errors recorded in the original literature were detected (e.g., recording a brachiopod occurrence from land, or recording latitude greater than 90° or longitude over 180°) and removed.

Finally, after data rationalization following the above raw data cleaning procedures, a secondary dataset consisting of 14918 geo-referenced occurrences from 394 living brachiopod species was obtained, which formed the basis of our subsequent analysis. Of this dataset, approximately 70% of the geo-referenced occurrences came from literature, and 80% of them have bathymetric information. Of the 394 species included in the final dataset, 19 (< 5%) are represented by only one single occurrence record in each case. They are rarity species and have been included in our analysis for the sake of maintaining data integrity and preserving potentially important biological and biogeographical signals that otherwise would have been lost had they been excluded. The challenge of potential sampling biases and their likely impacts on the robustness of the biogeographical patterns recognized here are discussed in the last section of this paper in order to provide a context for the conclusions reached in this study.

### Data visualization and selection of spatial scales

We used *The Biological Records Tool* function in QGIS 3.10 (QGIS Development Team, 2020,QGIS Geographic Information System, Open Source Geospatial Foundation Project: http://qgis.osgeo.org) for geospatial analysis and map visualization. Because we collected the data from variable geo-referenced sources with different locality resolutions, we used 5° latitude/longitude grid cells to project the visualization and display of species distribution at a global scale. The use of 5° grid cells offers one of the highest possible spatial resolutions while keeping potential artificial bias at a minimum; it also readily allows the visualization of global distribution maps of species under QGIS environment, as well as the comparisons with biogeographical patterns derived from other previous studies. The contour map was visualized based on the species richness value of 5° grid cells using the “contour” tool in the QGIS 3.10 software.

Although the use of latitudinal bins is a common approach in global biogeographical studies [29, 30], potentially it can introduce the effect of sampling bias because the latitude bins are of different area sizes. However, a plot of continental shelf area with brachiopod species richness (S1 Fig) failed to show a strong positive correlation between them. Therefore, if there had been any sampling bias introduced as a result of the use of latitudinal bins and 5° grid cells, the impact would be minimal, especially in regards to its effect on global biogeographical patterns. Similarly, we decided not to use any of the existing global marine biogeographical schemes [e.g., 15, 31] as the template for our study because our primary purpose was to reveal the global patterns of living brachiopod biogeography using a new and independent dataset without embedding any pre-conceived models.

### Sampling efficiency and data completeness

**Fig 1:**
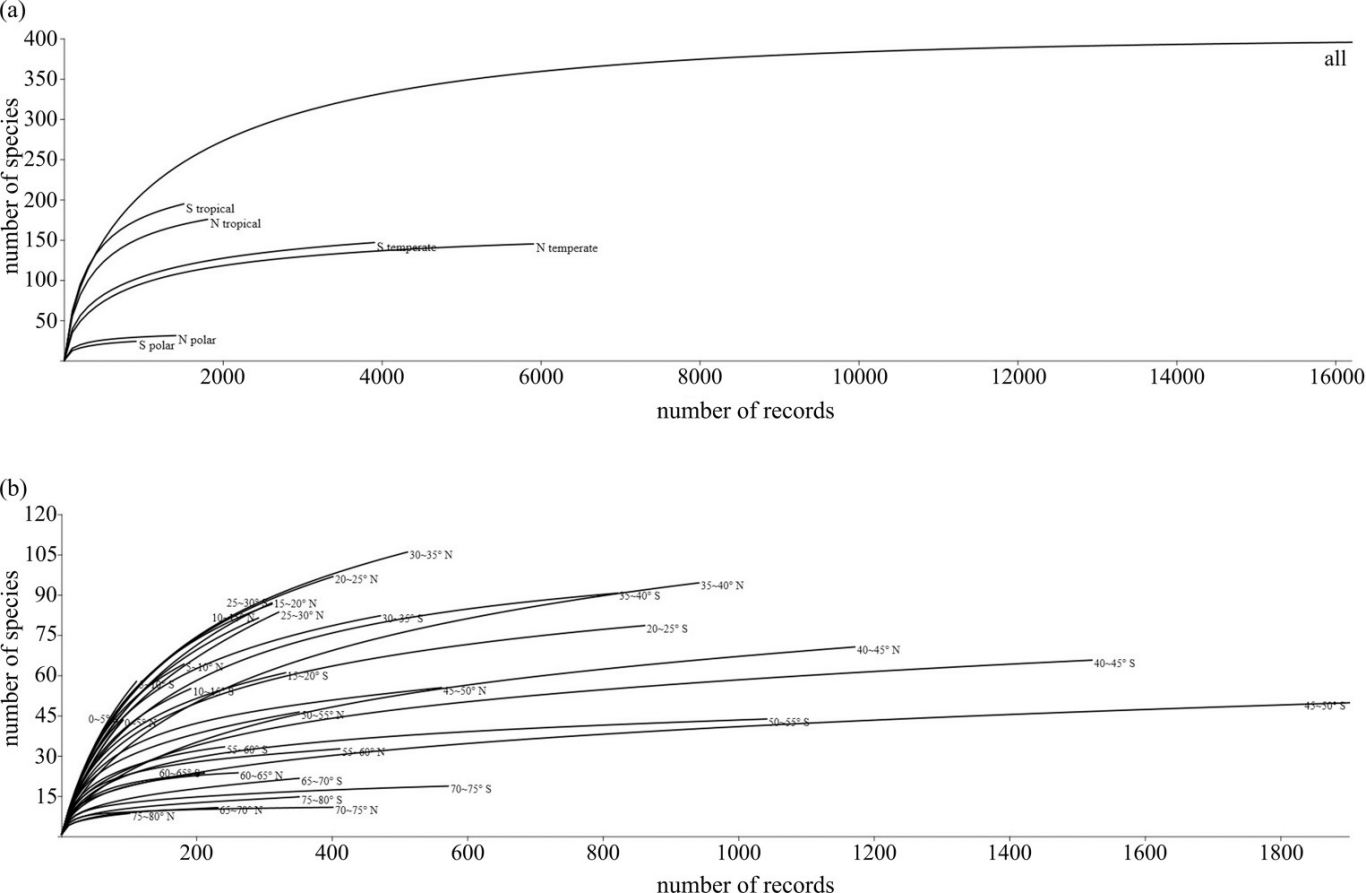
Sample rarefaction curves based on the data from our literature-derived database (software program PAST 4.01). (a), All: all data, N polar: data from 90° ~ 60° N, N temperate: data from 60° ~ 30° N, N tropical: data from 30° N ~ 0°, S polar: data from 90° ~ 60° S, S temperate: data from 60° ~ 30° S, S tropical: data from 30° S ~ 0°. (b), rarefaction curves for the data from each five-degree latitudinal bin.

To assess the robustness of our data sampling, we performed a rarefaction analysis [32, 33] and generated curves as a means to evaluating and illustrating the degree of data completeness, by using the PAST software (version 4.01, https://folk.uio.no/ohammer/past/, [34]) (Fig 1). Two different spatial resolutions were tested here. First, rarefaction analyses were performed on each of the three main climatic zones (tropical, temperate, and polar, each climatic zone roughly corresponds to a 30-degree latitudinal span) in each hemisphere (Fig 1A). Following this, we then carried out a second rarefaction analysis on each 5° latitudinal bin in both hemispheres with more details (Fig 1B). Overall, both rarefaction analyses indicate that most of the rarefaction curves demonstrate an asymptotic tendency, suggesting that further data input would not fundamentally affect the integrity of our database nor significantly alter the global patterns revealed in this study. Nevertheless, it is acknowledged that despite our best efforts, some caveats and sampling deficiencies may still remain in our dataset, as further elaborated and discussed in detail later in the paper (Discussion section).

### Statistical analysis

Data collections and plots were drawn in Microsoft Excel 2016. Package “Vegan” version 2.5–6, “Jaccard” method in R software (Version 4.0) was used for the agglomerative hierarchical cluster analysis procedures [35, 36]. Packages “factoextra” and “NbClust”, “Elbow” and “Silhouette” methods were performed in R to plot figure (S2 Fig). Cluster analysis was performed in two steps. In the initial clustering procedure, we included all raw 5° grid cells with ≥ 7 species richness for classifying grid cells into closely related (similar) cluster. This involved 180 grid cells, and the rationale here was to reduce data dimensionality of the original dataset to a level that the groupings between grid cells based on their mutual biotic similarities can be simplified and summarized into a smaller number of clusters, which can then be further synthesized through a secondary cluster analysis (step 2). In this initial procedure, the R software gave us the ability to compare and choose an optimal number of clusters (or K-means) through the so-called “Elbow method” and by balancing the number of clusters chosen with the higher average Silhouette width values (S2 Fig). “Elbow method” is a method designed to determine the optimal number of clusters quantitatively, by measuring the sum of the squared averages of all intra-cluster distances to their respective cluster centroids (i.e., total within sum of square or the y-axis in S2A Fig; the smaller the total within the sum of square values the more convergent of each cluster). Therefore, ideally we should pick the K-value where a bend (elbow) is located in the plot as an appropriate number, so that increasing clusters would not significantly alter the outcome of the analysis [37]. The so-called “Silhouette Method” measures how closely clusters are related to one another by deriving a value ranging from 1 to -1 (i.e., the average Silhouette width in S2B Fig): the higher the value the less related the clusters are to one another [38, 39]. In our study, the choice of a K value of two was clearly not appropriate as it would lead to the over-generalization of any potential inherent biogeographical structures within the dataset [40]. The next level of a higher average Silhouette width value appears when the K value is set at either 20, 23, 26 or 29 (S2B Fig). We performed cluster analysis and compared their dendrograms at these different K values (S3–S6 Figs). The result was that these dendrograms, regardless of the K value used, demonstrated strikingly similar grouping patterns. Consequently, for this study and further discussion, we chose the dendrogram derived with K = 20 as the output of our initial cluster analysis (S3 Fig). In this dendrogram, the 180 grid cells were grouped into 20 clusters. To further explore how these 20 clusters relate to one another hierarchically, we subjected these 20 clusters to a secondary cluster analysis with the aim to reveal the final and further simplified grouping structure of the dataset. Since the cluster analysis only involved 5° grid cells containing seven or more species, the grid cells included in this analysis were sourced mainly from coastal and shelf habitats where most (91% species and 76.4% occurrences of all data) living brachiopods occur. Consequently, the cells from other areas like small and remote oceanic islands and deep-sea settings were not included in this analytical procedure and, therefore, their contributions to the global biogeographical patterns of living brachiopods could not be assessed in the present study.

Next, to identify and visualize the faunal interlinkages between different bioregions, a network analysis was performed using the software Gephi version 0.9.2 [41] with settings of Force Atlas 2, number of threads 2, edge weight influence 1.0, scaling 10.0, gravity 1.0, tolerance 0.1 and approximation 1.2. The method of network analysis had been widely used in recent years as a promising tool in palaeobiogeography [42–46]. If the original dataset contains a robust hierarchical structure of biogeographical entities, the same or a highly comparable pattern should be revealed by both cluster analysis and network analysis with a high degree of complementarity and fidelity.

Because varying sets of biogeographical bioregions/bioprovinces could be delimited if different numbers of clusters are used [e.g., 47, 48], here three explicit criteria were applied to translate the cluster analysis result into an appropriate pattern of biogeographical bioregions/bioprovinces, following Kreft and Jetz [49], and Linder et al. [50]. First, the proposed bioregions/bioprovinces should not be nested into each other. Second, theoretically, the areas within the same bioregion/bioprovince should be geographically close. Finally, the hierarchical structure of biogeographical entities derived from the cluster analysis can also be supported by network analysis.

To further identify, characterize and distinguish potential biogeographical entities and possible subdivisions, we also considered and quantified a range of important biogeographical indices, including species and genus richness, typical taxa (genus and species), species/genus ratio (a proxy for biodiversification rate within an area, used by [30, 51]) and endemism (number of species unique to the biogeographical areas recognized) (Tables 2 and 3).

**Table 2:** A biogeographical division scheme of living brachiopods proposed in this study.

Bioregions	Bioprovinces	Cluster included (see Fig 5A)	Locality	Taxonomic composition character and (typical genera or species)	Biodiversity, number of species (genera) and diversification rate [species/genus ratio]	Endemism (number of endemic species)	Latitude and sea surface temperature ranges (temperature adopted from Climate Data Library: https://iridl.ldeo.columbia.edu/)	Key limiting ocean currents and upwelling systems
North Atlantic (A)		Clusters 4, 7, 8, 9	Northeast Atlantic & Mediterranean Sea, Caribbean & Gulf of Mexico	Largest family pool, most of Megathyrididae, (*Gryphus vitreus*, *Terebratulina retusa*)	117 (50) [2.34]	88 (75.2%)	10° N ~ 70° N	North Atlantic Gyre
							0~30°C	
	Northeast Atlantic & Mediterranean (A_1_)	Clusters 8, 9	Northeast Atlantic & Mediterranean Sea	No Lingulidae	60 (41) [1.46]	26 (43.3%)	15° N ~ 70° N	Canary current, Norwegian current, North Atlantic current, Canary upwelling
							0~30°C	
				exclusive distribution of Tethyrhynchiidae, (*Tethyrhynchia*, *Hispanirhynchia*)				
	Caribbean & Gulf of Mexico (A_2_)	Clusters 4, 7	Caribbean & Gulf of Mexico	**(***Tichosina*)	72 (31) [2.32]	46 (63.9%)	15° N ~ 35° N	Gulf Stream
							18~30°C	
North and West Pacific (B)		Clusters 1, 2, 12, 17, 18, 20	Northwest Pacific, Northeast Pacific, Japan & Indo-Malayan Archipelago	Most of Terebrataliidae, (*Frieleia*, *Laqueus*, *Terebratalia*, *Hemithiris*)	126 (52) [2.42]	82 (65.1%)	10° S ~ 65° N	North Pacific Gyre
							0~18°C	
	North Pacific (B_1_)	Cluster 12	Northwest Pacific & Japan, Northeast Pacific	Exclusive distribution of Cnismatocentridae, (*Cnismatocentrum*, *Tythothyris*)	43 (24) [1.79]	9 (20.9%)	30° N ~ 65° N	Oyashio current, Alaska current, North Pacific current, California current, California upwelling
							0~18°C	
	West Pacific, Indo-Malayan Archipelago (B_2_)	Clusters 1, 2, 18, 20	Japan & Indo-Malayan Archipelago	**(***Acanthobasiliola*)	101 (43) [2.35]	50 (49.5%)	10° S ~ 45° N	Kuroshio current, Equatorial Current
							4~30°C	
	California peninsula (B_3_)	Cluster 17	California peninsula	(*Dallinella*?	20 (15) [1.33]	2 (10%)	30° N ~ 35° N	California current, California upwelling
				*Terebratalia*?			18~30°C	
				*Glottidia palmeri*)				
West Indian Ocean (C)		Clusters 3, 11, 15	Red Sea, Madagascar, South Africa, East Africa		74 (42) [1.76]	40 (54.1%)	35° S ~ 30° N	Somali current, Agulhas current, Equatorial current
							18~32°C	
	West Indian Ocean (C_1_)	Clusters 3, 15	Madagascar, South Africa, East Africa	**(***Agulhasia*,	67 (37) [1.81]	33 (49.3%)	35° S ~ 0°	Somali current, Agulhas current, Equatorial current
				*Kraussina*,				
				*Megerlina*?				
				*Megerella hilleri*)			18~30°C	
	Red Sea (C_2_)	Cluster 11	Red Sea	No Craniidae and Terebratulidae, (*Hillerella*, *Argyrotheca cooperi*)	12 (10) [1.2]	4 (33.3%)	13° N ~ 30° N	
							18~32°C	
Southwest Pacific (D)		Clusters 1, 2, 13, 19	Southeast Australia, New Caledonia, Fiji, Tonga Islands		78 (47) [1.66]	34 (43.6%)	45° S ~ 10° S	East Australian current
							10~30°C	
	Southeast Australia (D_1_)	Clusters 1, 2, 13	Southeast Australia	**(***Anakinetica*,	33 (25) [1.32]	12 (36.4%)	45° S ~ 20° S	East Australian current
							10~28°C	
				*Parakinetica*,				
				*Murravia exarata*				
				*Aulites brazieri*?**)**				
	New Caledonia & Fiji (D_2_)	Cluster 19	New Caledonia, Fiji, Tonga Islands	**(***Kanakythyris*,	55 (37) [1.49]	18 (32.7%)	30° S ~ 10° S	East Australian current
				*Basiliollela*,			22~30°C	
				*Stenosarina*?				
				*Leptothyrella fijiensis*, *Xenobrochus rotundus*, *Campages ovalis***)**				
Circumpolar Antarctic (E)		Clusters 4, 5, 6, 10, 14	Antarctica, South America, New Zealand	No Lingulidae, (*Acrobrochus*, *Liothyrella*)	85 (51) [1.67]	45 (52.9%)	75° S ~ 30° S	West wind drift, East wind drift
							0~22°C	
	Antarctica (E_1_)	Cluster 6	Antarctica	**(** *Compsothyris* **)**	28 (21) [1.33]	5 (17.9%)	75° S ~ 65° S	East wind drift
							0~6°C	
	Southern America (E_2_)	Cluster 10	South America	**(***Terebratella*,	28 (22) [1.27]	5 (17.9%)	65° S ~ 30° S 0~20°C	West wind drift, Peru current, Humboldt upwelling
				*Magellania*,				
				*Magellania venosa* **)**				
	New Zealand (E_3_)	Cluster 14	New Zealand	Exclusive distribution of Notosariidae, (*Calloria*, *Gyrothyris*, *Neothyris*, *Notosaria*, *Pumilus antiquatus*)	55 (38) [1.45]	17 (30.9%)	55° S ~ 30° S	West wind drift, East Australian current
							8~22°C	
	South Indian Ocean (E_4_)	Clusters 4, 5	South Indian Ocean	**(***Aerothyris*,	19 (13) [1.46]	3 (15.8%)	55° S ~ 35° S	West wind drift
							0~18°C	
				*Pemphyxina* **)**				
Independent provinces								
	Galapagos	Cluster 5	Galapagos	**(** *Gryphus clarkeana* **)**	10 (9) [1.11]	2 (20%)	5° S ~ 5° N	Equatorial current
							20~28°C	
	Hawaii	Cluster 1	Hawaii	**(** *Basiliola beecheri* **)**	11 (9) [1.22]	2 (18.2%)	20° N ~ 25° N	
							24~28°C	
	Amsterdam-St Paul	Cluster 5	Amsterdam and Saint-Paul Islands	**(***Striarina*,	7 (7) [1]	2 (28.6%)	35° S ~ 40° S	West wind drift
							14~20°C	
				*Pemphyxina*,				
				*Megerlina davidsoni* **)**				

**Table 3:** A summary of comparisons between the biogeographical scheme proposed here and several classic existing schemes.

Bioregions	Bioprovinces	Corresponding biogeographical units as proposed by Zezina [15]	Difference comparing between Bioprovince proposed in this study and recognised by Zezina [15]	Corresponding biogeographical units as proposed by Spalding et al. [31]	Difference comparing between Bioprovince proposed in this study and recognised by Spalding et al. [31]
North Atlantic (A)					
	Northeast Atlantic & Mediterranean (A_1_)	North Atlantic, Lusitanian-Mauritanian-Mediterranean	Boundary of A_1_ Extends further North to northern Norway and further south to Cape Verde Islands	Northern European Seas, Lusitanian, Mediterranean Sea, West African Transition	Corresponding to four provinces
	Caribbean & Gulf of Mexico (A_2_)	West Atlantic, Caribbean	Not included Bermuda Islands	Warm Temperate Northwest Atlantic, Tropical Northwestern Atlantic	Corresponding to two provinces
North and West Pacific (B)					
	North Pacific (B_1_)	North Pacific, Californian	Southern border of B_1_ may overlap with B_2_ and B_3_	Cold Temperate Northwest Pacific, Cold Temperate Northeast Pacific	Corresponding to two provinces, southern border of B_1_ locates further north.
	West Pacific, Indo-Malayan Archipelago (B_2_)	North Pacific, West pacific, Indo–West Pacific, Japanese	Included all Japanese, but only part of North Pacific, West pacific, Indo–West Pacific	Cold Temperate Northwest Pacific, Warm Temperate Northwest Pacific, South China Sea, South Kuroshio, Western Coral Triangle	Corresponding to five provinces, northern border of B_2_ locates further north.
	California peninsula (B_3_)	Californian	Consistent with Californian	Warm Temperate Northeast Pacific	Consistent with Warm Temperate Northeast Pacific
West Indian Ocean (C)					
	West Indian Ocean (C_1_)	South African	Added Madagascar and the East Coast of Africa, not included Prince Edward Islands	Somali/Arabian, Western Indian Ocean, Benguela, Agulhas	Corresponding to three provinces, not included Arabian Gulf
	Red Sea (C_2_)			Red Sea and Gulf of Aden	Consistent with Red Sea and Gulf of Aden
Southwest Pacific (D)					
	Southeast Australia (D_1_)	South Australian	Consistent with South Australian	East Central Australian Shelf, Southeast Australian Shelf, Southwest Australian Shelf	Corresponding to three provinces
	New Caledonia & Fiji (D_2_)	West Pacific, Indo–West Pacific	Part of West Pacific, Indo–West Pacific	Tropical Southwestern Pacific, Lord Howe and Norfolk Islands	Not included Lord Howe Islands
Circumpolar Antarctic (E)					
	Antarctica (E_1_)	Antarctic	Not included the part of South America	Scotia Sea, Continental High Antarctic	Corresponding to two provinces
	Southern America (E_2_)	South American	Consistent with South American	Warm Temperate Southeastern Pacific, Warm Temperate Southwestern Atlantic, Magellanic, Scotia Sea	Mainly corresponding to Magellanic, included part of other three provinces
	New Zealand (E_3_)	New Zealand	Consistent with New Zealand	Northern New Zealand, Southern New Zealand, Subantarctic New Zealand	Corresponding to three provinces
	South Indian Ocean (E_4_)	Kerguelen, Crozet, Prince Edward Islands	Consistent with Kerguelen, Crozet, Prince Edward Islands	Subantarctic Islands	Consistent with Subantarctic Islands
Independent provinces					
	Galapagos				
	Hawaii	West Pacific	Corresponds to Hawaiian Islands of West Pacific	Hawaii	Consistent with Hawaii
	Amsterdam-St Paul	Nouvelle Amsterdam	Consistent with Nouvelle Amsterdam	Amsterdam–St Paul	Consistent with Amsterdam–St Paul

## Results

**Fig 2:**
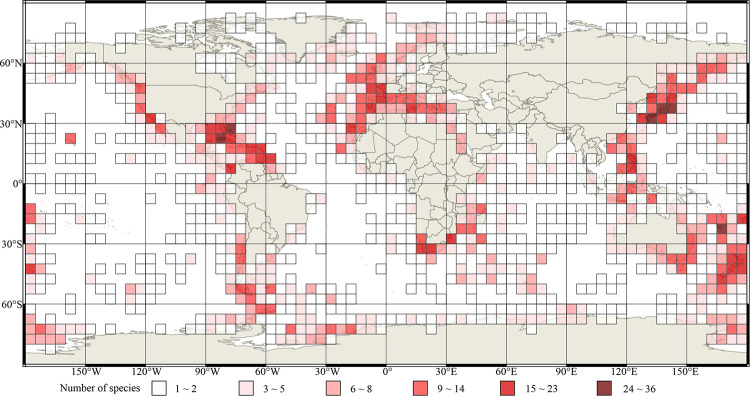
5° grid cell map of species richness distribution of living brachiopods. Different shades of red colour represent the gradient of species richness as shown in the legend boxes. Source: global basic map was downloaded from ArcWorld Supplement via ESRI and [52]), then adapted for visualization here by using open source Geographic Information System QGIS (http://qgis.osgeo.org).

**Fig 3:**
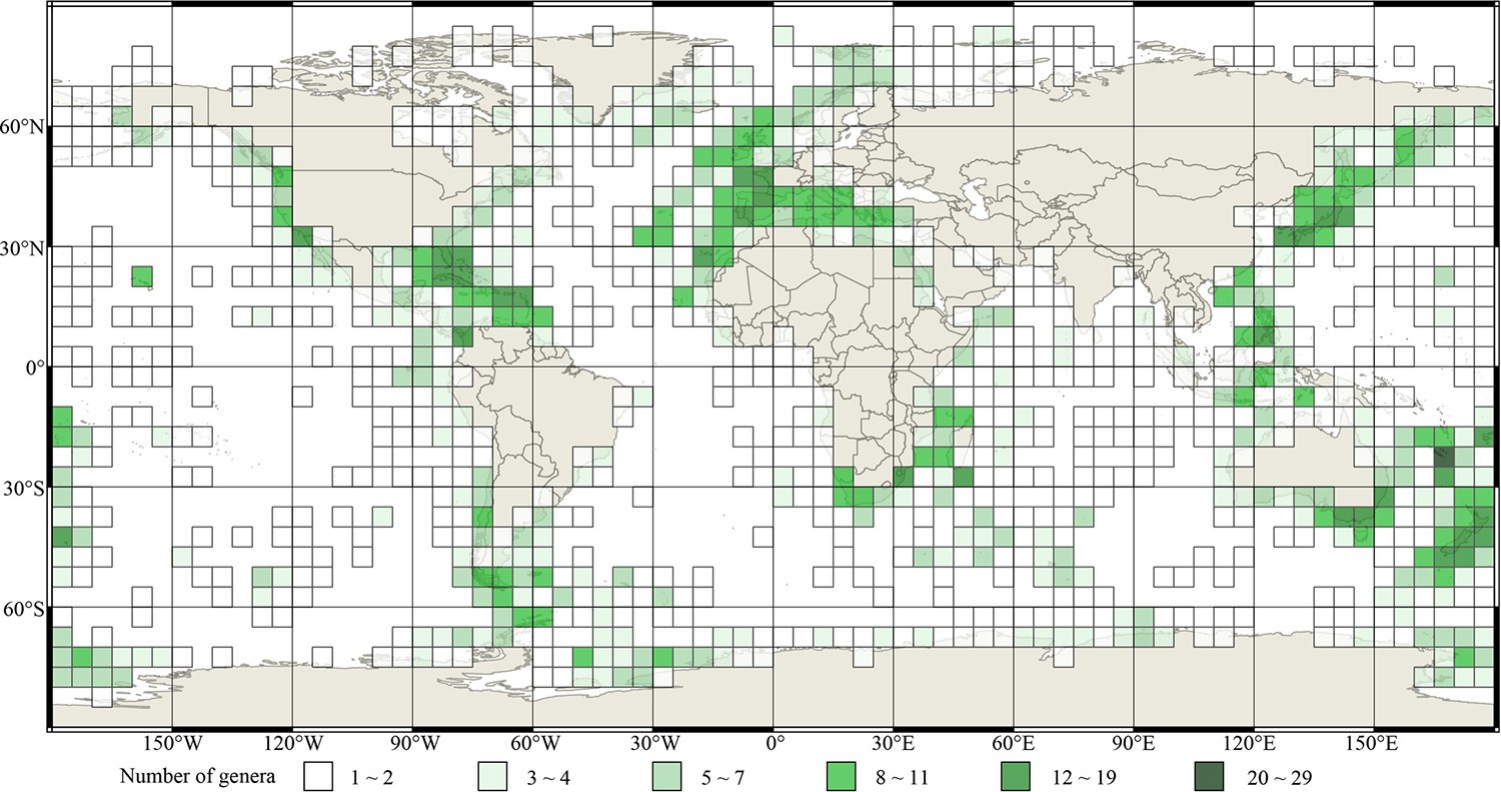
5° grid cell map of genus richness distribution of living brachiopods. Different shades of green colour represent the gradient of genera richness as shown in the legend boxes. Source: same as in Fig 2.

**Fig 4:**
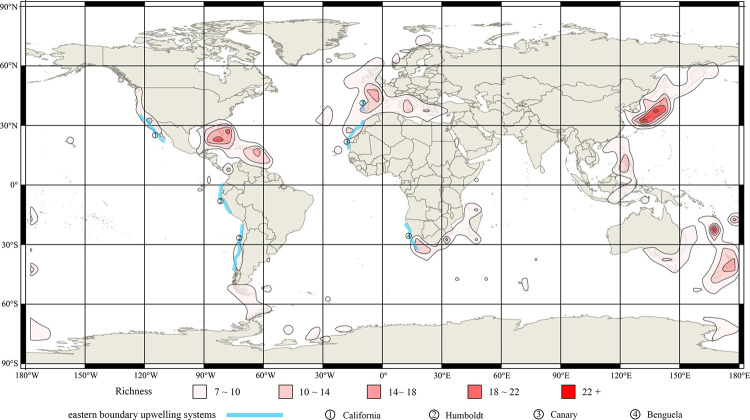
Contour map of living brachiopod species richness based on 5° grid cells. Coastal upwelling regions are adjusted from [53] and NOAA (https://www.noaa.gov/). Source: same as in Fig 2.

The global distribution patterns of living brachiopod diversity are shown in 5° grid cells (species and genus, Figs 2 and 3 respectively). Overwhelmingly, most records and the vast majority of brachiopod species (Fig 2) and genera (Fig 3) are located in shallow water environments < 200 m (including the continental shelf, shallow waters surrounding archipelagos, and shallow seamounts). Spatial contour mapping of species richness distribution exhibits a number of well-defined areas of relative high species richness (or biodiversity hotspots) (Fig 4): including Northwest Pacific-Japan, Northeast Pacific, Indo-Malayan Archipelago, Caribbean-Gulf of Mexico, Southeast Australia-New Zealand-New Caledonia, Northeast Atlantic-Mediterranean Sea, Southern Africa-Madagascar, and Southern America. Three 5°grid cells each with >30 species were identified, two located around Japanese islands and adjacent sea waters (two cells: “35°N130°E-30°N135°E”, “40°N135°E-35°S140°E”), and one in the vicinity of New Caledonia (one cell: “20°S165°E-25°S170°E”); these are the world’s richest living brachiopod species centres.

**Fig 5:**
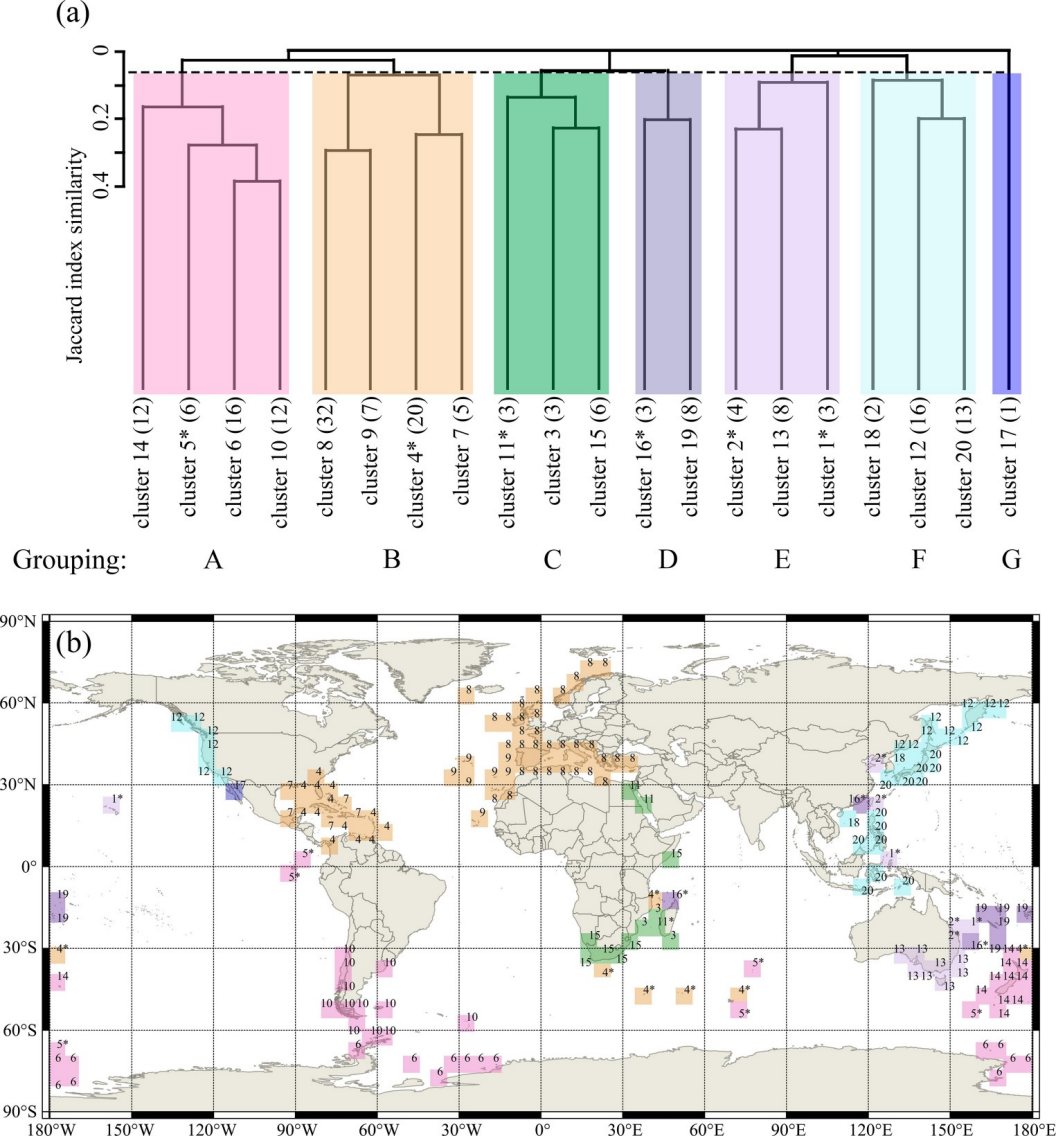
Cluster analysis result and mapping. (a) dendrogram of cluster analysis showing the seven major groups (A, B, C, D, E, F, G), the numbers in brackets are the number of 5° grid cells included in each cluster; (b) global distribution of the seven major groupings recognized in (a) and their constituent clusters. The variety of colours corresponds between the two subfigures and represent the seven different group (A through G); the labelled number on each 5° grid cell corresponds to the cluster number in (a). Asterisk denotes clusters whose group memberships are equivocal due to possible under-sampling or other reasons and require careful consideration; these are usually clusters that contain widely scattered grid cells of relatively small sizes. Source: same as in Fig 2.

Seven core groupings were delineated by the cluster analyses and are named Grouping A through to Grouping G in Fig 5A. The grid cells corresponding to the different clusters are visualized in different colours on both the dendrogram (Fig 5A) and the global distribution map (Fig 5B). In most cases, grid cells assigned to the same cluster are close geographically (Fig 5B). In the secondary cluster analysis of the 20 initially recognized clusters, seven major groupings were demarcated at a Jaccard similarity cut-off score of 0.065 (i.e., a dissimilarity of 0.935) (Fig 5A) [54]. Though all clusters were aggregated to form one of the seven major groupings, the coherence of an individual cluster to a particular grouping somewhat varies. In particular, there are four clusters (clusters 1, 2, 5, 11, 16) whose grouping membership appears problematic and requires verification through the ensuing network analysis (see Discussion section). Typically, these clusters have a smaller number of grid cells of scattered distribution.

**Fig 6:**
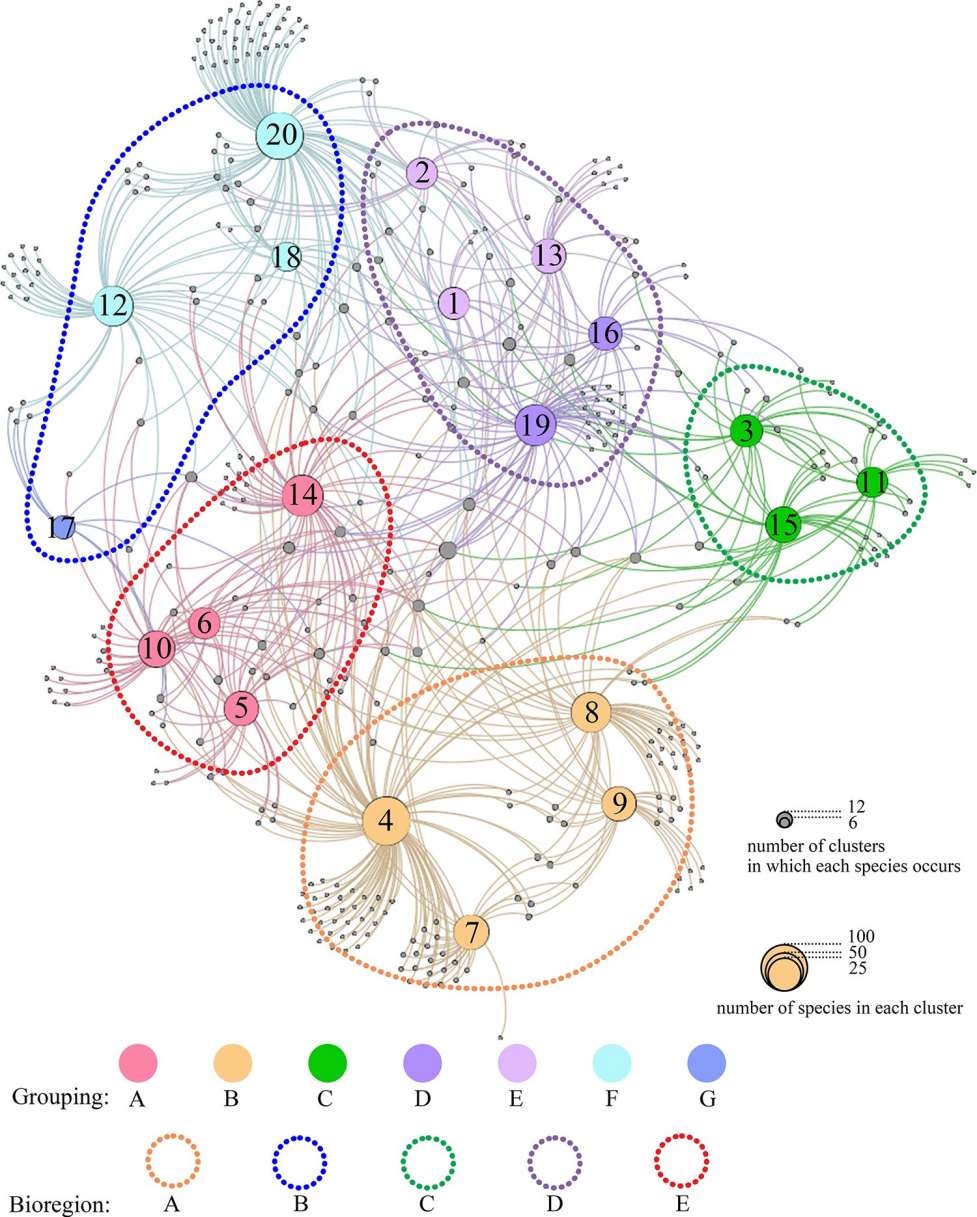
Output of network analysis depicting the degree of major groupings and connectivity among the 20 clusters and the brachiopod species. The variety of node colours and labelled numbers are the same as in Fig 5; the diameter of the nodes represents the number of species in each cluster (nodes with cluster number and colours) and number of clusters in which each species occurs (grey nodes) respectively.

**Fig 7:**
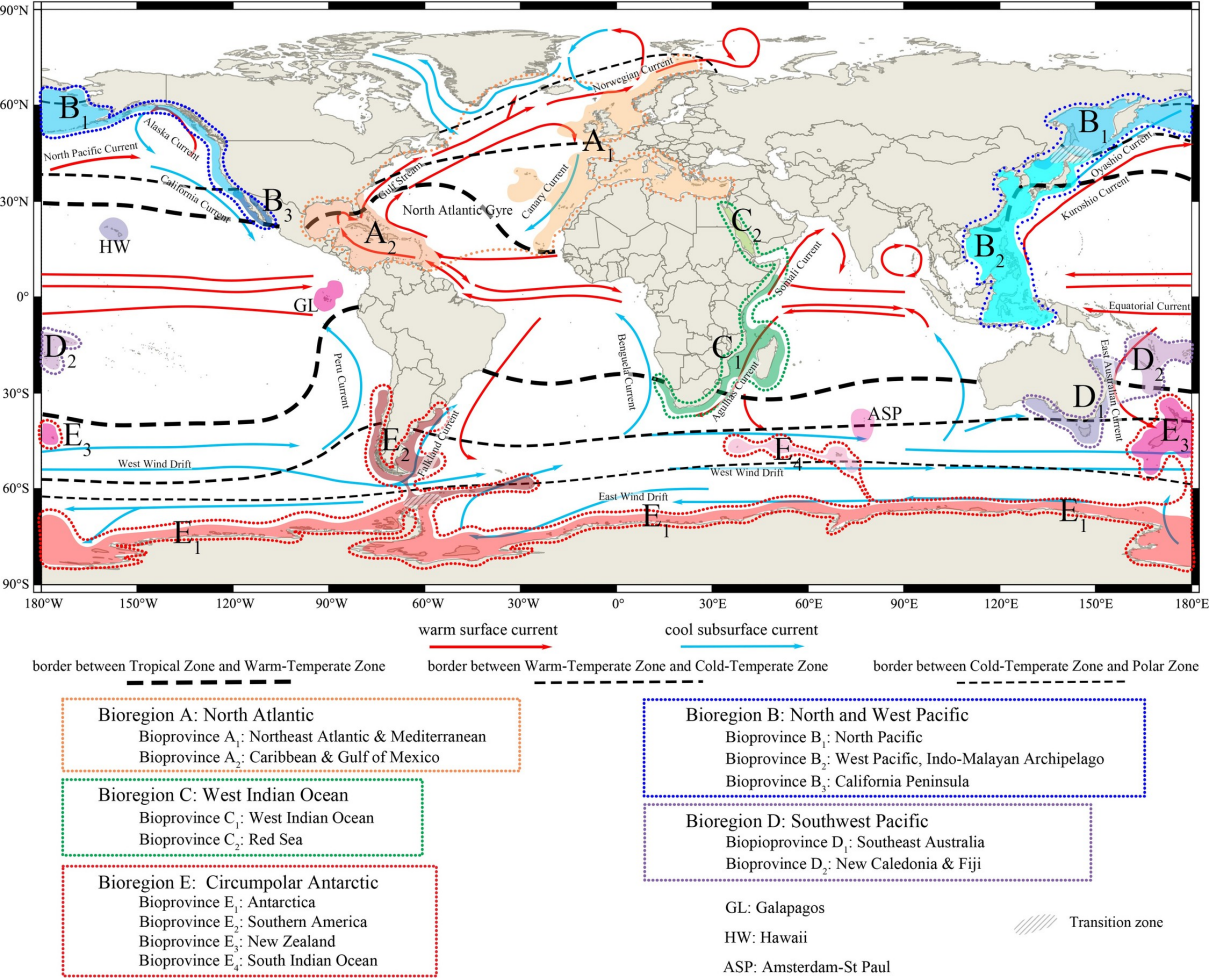
World map showing the global biogeographical regions and provinces proposed in the present study. Global ocean currents are adopted from NOAA (https://www.noaa.gov/), climatic zones are adjusted from Briggs [55, 56]. Source: same as in Fig 2.

The same seven major groupings recognized by the cluster analysis were confirmed by the network analysis (Fig 6), which additionally also displays the spatial disposition of the clusters with respect to their relative closeness (i.e., biogeographical similarity) to one another. On the other hand, when the graphical outputs of the cluster and network analyses are compared, there are some minor differences in the grouping structure displayed by these two different methods, mainly reflected in the grouping of three clusters (clusters 16, 17 and 19). Cluster 17, whose grouping membership is equivocal according to the cluster analysis, actually falls between Groupings A and F in the network analysis (Fig 6) and thus seems assignable to either of these groupings. As will be discussed further below, notwithstanding the possible impact of sampling bias on its biogeographical identity and affinity, it seems more appropriate to associate this cluster to Grouping B (Fig 7) in respect of its geographical location and some peculiar characteristics of its brachiopod fauna. In a similar way of reasoning, clusters 16 and 19 (Grouping D), which were shown seemingly more closely related to Grouping C by cluster analysis, however, exhibit closer ties to Grouping E by network analysis (Fig 6). Nevertheless, considering the geographical proximity of clusters 16 and 19 to Eastern Australia, we have grouped these two clusters together with cluster 13 and part of clusters 1, 2, all within Grouping D (Fig 7).

In summary, combining the results of cluster and network analyses with a consideration of species composition and endemism, as well as a reference to some existing global benthic marine biogeographical schemes [e.g., 15, 31], a new global bioregionalization scheme for living brachiopods can be recognized, consisting of thirteen bioprovinces in five bioregions (Table 2, Fig 7). In this scheme, the bioregions mostly correspond to the major groupings recognized by the cluster analysis, verified by network analysis, while the 13 bioprovinces are most represented by a single cluster or a combination of clusters. The characteristics and origin of these biogeographical entities are summarized in Table 2 and further discussed in the following section.

## Discussion

### Characterization and formation of brachiopod biogeographical bioregions and bioprovinces

#### Bioregion A (The North Atlantic Bioregion)

Bioregion A is here named the North Atlantic Bioregion for living brachiopods. This bioregion has the highest endemism (75.2%) and second-highest number of species (117) and the second-highest species diversification rate (2.34) at the bioregion level (Table 2). The spatial extent of this bioregion coincides with the spatial limits of the North Atlantic Gyre (Fig 7), suggesting that sea surface ocean currents with their confined water temperature ranges must be one of the critical factors controlling the formation and range of the bioregion.

The bioregion is readily divisible into two distinct bioprovinces: the Northeast Atlantic−Mediterranean Bioprovince (A_1_) and the Caribbean−Gulf of Mexico Bioprovince (A_2_). A_1_ extends from the offshore waters of northern Norway in the north to the Canary Islands in the south, including the British Isles, Azores Islands, Madeira Island, as well as the Mediterranean Sea. South of the Canary Islands, the coastal areas of Northwestern Africa including the Cape Verde Islands are also included in this bioprovince, as indicated by the cluster analysis (Fig 5B). This southward extension and connection of A_1_ can be explained by the occurrence of the Canary Current which has been active since the Pliocene [57], facilitating and maintaining planktotrophic larvae and faunal connection across these shelf waters. The southern limit for A_1_, as defined here, is different from the southern boundary of Zezina’s [15] ‘North Atlantic type’ of biogeographical division, which was considered to extend southward only to the Canary Islands (Table 3). Our consideration of this southern boundary extending beyond south of the Canary Islands is supported by the continued presence of *Novocrania anomala*, *Argyrotheca cuneata*, *Megathiris detruncata*, *Leptothyrella incerta* in Canary Islands and Cape Verde Islands [58].

Similarly, cluster analysis does not permit us to separate the Mediterranean Sea as an independent bioprovince, with only two endemics *Tethyrhynchia mediterranea* and *Lacazella mediterranea* [59]. This is largely because the Mediterranean Sea and East Atlantic Coast share many brachiopod species that are thought to have either entered or re-entered the Mediterranean Sea from the Atlantic Ocean since the Pliocene after the Messinian salinity crisis [59], such as genera *Gryphus*, *Argyrotheca*, *Megathiris*, *Megerlia*, and *Novocrania*.

Thus, it is evident that the Northeast Atlantic−Mediterranean Sea Bioprovince spans both the Warm- and Cold-temperate waters of the entire northeastern Atlantic shelf, as well as the Mediterranean Sea. The large latitudinal range of this bioprovince, from 10° N to 70° N, can be explained by the clockwise movement of the North Atlantic Gyre, which brings relatively warm water masses from the Caribbean Sea to the Northeast Atlantic. Thus this warm current is not only warming up the otherwise would-be cool waters of the northeastern Atlantic coast, but also promoting the dispersal capacity of brachiopod larvae across the Atlantic and allowing some temperate species to colonize habitats as far north as Arctic Norway [60].

In contrast, the second bioprovince of the North Atlantic Bioregion, the Caribbean−Gulf of Mexico Biorovince (A_2_), is spatially restricted, being largely confined to the Caribbean Sea and the Gulf of Mexico, including the shelves of the Greater Antilles and the Lesser Antilles. A_2_ is composed of two clusters, cluster 4 and cluster 7 (Fig 5B). Whilst cluster 7 is confined to this province, the distribution of cluster 4 is highly heterogeneous in our initial cluster analysis when the K value was chosen at 20, manifested by a concentration in the Caribbean Sea (A_2_) and subordinate presence in the southern Indian Ocean (E_4_), New Zealand (E_3_) as well as in southern and eastern Africa (C_1_) (Fig 5B). Whilst the wide scatter of cluster 4 may reflect an underlying under-sampling issue or a complication caused by the cluster possessing some authentic disjunction distributions, appoint that will be further discussed below. However, its strong relationship with cluster 7 is confirmed when we selected K equal to 23, 26 or 29 (S4–S6 Figs). For this reason, we have treated cluster 7 and grid cells of cluster 4 in the Caribbean−Gulf of Mexico region as one coherent province. This bioprovince is consistent with what Zezina [15] called the ‘West Atlantic type” of distribution, accompanied with most records of living *Tichosina*, which are absent in A_1_. Meanwhile, A_2_ also has the highest endemism (63.9%) and the second highest diversification rate (2.32) in bioprovince level (Table 2). Climatically, this bioprovince is nested entirely within the reaches of the Tropical to Subtropical zones and influenced by the Gulf Stream in the western side of the North Atlantic Gyre (Fig 7).

Though recognized as two distinct bioprovinces, there is a strong similarity in species composition between A_1_ and A_2_ bioprovinces, as already noted previously by Cooper [61]. In the present study, 20 species, or 33% species of A_1_ and 27% species of A_2_, were identified to occur in both bioprovinces, suggesting frequent and sustained faunal exchanges between them. This strong biogeographical link can be readily explained by the operation of the North Atlantic Gyre (Fig 7). Potentially, the passage of this gyre would facilitate the dispersal of brachiopod larvae from the Tropical-Subtropical Caribbean Sea, northeastward across the North Atlantic Ocean, to the cold-temperate waters of Northeast Atlantic. Evidence for this long-distance, trans-oceanic faunal communication has been found in a number of living brachiopod species. One of these examples is demonstrated by the distribution of *Terebratulina retusa*, whose larval dispersal is known to closely follow the path of the North Atlantic Current [60]. However, the degree of faunal exchange between the two bioprovinces in the southern part of Bioregion A appears less frequent or weaker compared to the North Atlantic region, as exemplified by the distribution of *Thecidellina barretti*. Initially, Logan [62] thought that this species was distributed across the Atlantic Ocean from the Caribbean to Eastern Atlantic (Cape Verde islands), but a recent investigation [63] confirmed that *T*. *barretti* originally found in Cape Verde by Logan [62] actually belongs to a new species. This indicates that, despite the potential biogeographical connection that may arise from the North Atlantic Equatorial Current, the wide oceanic separation between the Caribbean Sea and the Eastern Atlantic coast seems to have acted as an effective barrier hindering the biogeographical exchange in the southern part of the North Atlantic Bioregion, at least for some living brachiopods.

Recently, some new living brachiopod materials from the Northern Mid-Atlantic Ridge have been reported, which has provided evidence for a previously unknown biogeographical dispersal mechanism and pathway between the low-latitude of A_1_ and the middle−high latitude of A_2_, evidently through the deepsea mid-ocean ridge systems [64]. In this context, it is also worth noting the geohistorical development of the A_1_ and A_2_ bioprovinces with respect to their respective geological settings and relationships with their nearest other biogeographical bioprovinces. According to our cluster analysis, the North Atlantic−Mediterranean Sea Bioprovince (A_1_) is clearly separable from the Red Sea Bioprovince (C_2_) (Fig 5), but this biogeographical separation appears to have become apparent only since the Miocene when the ancient Tethyan seaway became finally closed off [65]. Before the Miocene when this seaway remained open connecting the Mediterranean Sea with the Indo-Pacific oceans, there were widespread and free faunal exchanges across these ocean basins leading to strong mutual faunal affinities [66–69]. The Miocene closure of the Tethyan seaway had proved to be an important biogeographical vicariance event in not only separating the Northeast Atlantic−Mediterranean Sea Bioprovince from the Red Sea Bioprovince, but also providing an effective mechanism for allopatric speciation, as demonstrated by the phylogeographic relationships of post-Miocene *Lingula* species in the Mediterranean Basin [10, 70–74].

A comparable biogeographical vicariance event is believed to have also occurred across the Central American Isthmus. Evidence in support of this scenario comes from the biodistribution of the genus *Glottidia*, which only occurs along both the western and eastern shelf water environments of central America and the coast of Baja California. Previous prevailing view believed that the ancestral *Glottidia* first appeared on the western shelf of North/Central America, and then migrated to Atlantic Ocean from west to east in the Palaeogene when Central America existed as an open seaway (the Panama Seaway) [10]. More recently, however, new evidences from chemico-structural and molecular genetic data favoured a dispersal route from west Tethyan (England) to the Atlantic Ocean via the North Equatorial Current [75]. Notwithstanding the validity of either hypothesis, both accepted that there was open faunal connection between the Caribbean−Gulf of Mexico Bioprovince and eastern Pacific before the Panama seaway was closed. After the seaway was completely closed off in the Pliocene, forming the Central American Isthmus [76, 77], faunal exchange was stopped across the isthmus, which led to divergence and allopatric speciation of *Glottidia* on each side of Central America; for example, *Glottidia audebarti*, *Glottidia albida*, *Glottidia palmeri* and *Glottidia semen* are known only from the Pacific side of Central America where their distributions may overlap, whereas *Glottidia pyramidata* is found only on the Atlantic side of Central America [78]. Also of note in this context is the relative young age for many of these species (*G*. *albida*: Late Eocene; *G*. *palmeri*: Holocene; *G*. *pyramidata*: Holocene; [75, 79], further supporting the recent evolutionary divergence of these species as a result of the emergence of the Central American Isthmus.

#### Bioregion B (The North Pacific Bioregion)

This bioregion is distributed in the northern, western and northeastern shelf regions of the Pacific Ocean; as such, its spatial extent largely overlaps with the distribution of the North Pacific Gyre (Fig 7). This bioregion has the largest species richness in this study (with 126 species), the second highest species endemism at 65.1% and the highest species diversification rate (2.42) (Table 2). These metrics may indicate that the bioregion has long been established and in isolation from other bioregions, leading to a high rate of both speciation and species accumulation.

The bioregion encompasses three bioprovinces: the North Pacific (B_1_), West Pacific−Indo-Malayan Archipelago (B_2_) and the Californian Peninsula (B_3_). B_1_ is situated in the cold temperate coastal zone of the North Pacific Ocean, extending from Hokkaido Island in the west, then north- and eastward through Alaska to California in the east. To the north, the narrow Bering Strait and the high Arctic cold polar waters seem to have provided an effective barrier separating this bioprovince from the Arctic, despite the existence of limited joint occurrences of some circumpolar Arctic elements (e.g., *Hemithiris psittacea* and *Glaciarcula spitzbergensis*) in both the northern part of A_1_ and B_1_ [15]. We believe that free migration between the North Pacific, Arctic, and North Atlantic should have occurred no earlier than the opening of the Bering Strait in the Miocene [80, 81].

The southern boundary of B_1_ seems generally coinciding with the boundary between the Warm-temperate and Cold-temperate climatic zones on either side of the North Pacific (Fig 7). However, the details of the transition between B_1_ and its southern neighbouring bioprovinces are in fact more complicated and suggest the appearance of biogeographical ecotones (biogeographical mixing zones). On the one hand, in both sides of the North Pacific, the southern boundary of B_1_ may extend further south beyond the Warm-temperate/Cold-temperate climatic boundary and overlaps with B_2_ in the west and B_3_ in the east, as the distribution of some brachiopods such as *Frieleia halli*, *Diestothyris frontalis*, *Terebratulina kiiensis* and *Terebratulina crossei* actually can reach to the oceanic shores of Japan in the West Pacific and near the Baja California Peninsula in the East Pacific [9, 15]. On the other hand, at least 23 living brachiopod species from the Tropical and Warm-temperate B_2_ are known to also occur in B_1_, resulting in a distinctive biogeographical ecotone within the northern part of the Japan Sea and adjacent outer shelf areas east of Japan (Fig 7). The formation of this biogeographical transitional zone is not unique to living brachiopods; it has also been recognized in a number of other marine taxa [e.g., 82, 83] and has generally been attributed to the convergence of both the warm-water Kuroshio and the cold-water Oyashio currents in the vicinity of the Japanese islands, enabling the intermingling of both warm-water and cold-water species in these areas [84, 85]. The strength of this mixed marine fauna in terms of the proportion of cold-water elements relative to warm-water elements has varied over time, depending on the strength of the two major ocean currents relative to one another at any time. The latter, in turn, is very much controlled by the dynamics of sea levels affected by the glacial-interglacial cycles [86, 87].

B_2_ is located largely in the central-west coast of the Pacific Ocean, extending from Hokkaido Island south to the Banda Sea in Southeast Asia and spanning both the Tropical and Warm-temperate climatic zones. Among all the bioprovinces recognized in this study, B_2_ attains the largest species pool, with 101 species, and it is accompanied by an equally high level of species endemism at 49.5%. Both of these metrics suggest that B_2_ likely represents a centre of speciation. A significant number of genera (e.g., *Acanthobasiliola*, *Shimodaia*) and species (e.g., *Zygonaria davidsoni*) are exclusively found in this bioprovince [88].

As already noted, the northern part of B_2_ overlaps with the southern part of B_1_, but as indicated by our cluster analysis (Fig 5), its southern boundary appears to be more sharply defined by the Banda Sea. The seemingly abrupt separation of B_2_ from the Southwest Pacific bioregions (bioregion D in Fig 7) is somewhat difficult to explain using the present-day geography of the Indo−West Pacific region where the Equatorial Current of the Pacific today partly passes through into the Indian Ocean and is also partly deflected southward toward eastern Australia. Even though the distribution of living *Lingula* can extend from the Japan Sea in the North until New South Wales coast, Australia in the south [10, 68], the exclusive distribution of genera *Acanthobasiliola* and *Zygonaria* in B_2_, coupled by the absence of *Basiliolella*, which is otherwise common in the bioregion D, would suggest that the southern limit of the Banda Sea constituted an effective biogeographical boundary separating bioregions B and D in recent geological past. The origin of this boundary cannot be readily explained using present-day geography or oceanography, but can be understood if it is linked to the recent history of eustatic changes in this region. Previous studies have established that south of the Band Sea, there exited a land bridge between New Guinea and Australia in the Pleistocene [89–91]. Accepting this scenario, the land bridge would have severely limited the marine biogeographical connections between the North and West Pacific Bioregion (Bioregion B) and the South Pacific Bioregion (Bioregion D) (Fig 7).

Compared to B_1_ and B_2_, B_3_ (California Peninsula Bioprovince) is much smaller in geographical extent, being represented by only one cluster (cluster 17 in Fig 5A) located in the surrounding waters around the California Peninsula. It is situated entirely within the Warm-temperate climatic zone of the eastern Pacific coast, where both the Californian cold-water current and the Californian coastal upwelling system occur. The brachiopod fauna here is characterized by a relatively low diversity (20 species), high dominance (40% of the species are Lingulida, Discinida or Craniida) and low endemism (10%) (especially abundant *G*. *palmeri* and *G*. *audebarti*). The combination of these characteristics makes this bioprovince unique and may explain why it stands out seemingly as a statistical outlier in the cluster analysis (Fig 5A). The low endemism may be explained by the shared wide distribution of *Discinisca* and *Lingula* within the Pacific, as already noted by Emig [10]. On the other hand, the widely distributed Pacific *Discinisca* and *Lingula* have not been reported in the Caribbean Sea (A_2_), even though A_2_ and B_3_ are geographically close and located in similar latitudinal positions. Very likely, this distinction has arisen because of the existence of the Central American Isthmus acting as a major agent for biogeographical vicariance between the Pacific and Atlantic ocean basins, apparently since the Miocene [92].

#### Bioregion C (The West Indian Ocean Bioregion)

Bioregion C is located almost entirely within the Tropical Zone of the West Indian Ocean, and includes the Red Sea, the shelf waters of Madagascar, and other offshore areas of eastern South Africa (Fig 7). Of all the brachiopod bioregions recognized in this paper, Bioregion C has the lowest species richness (74), but still with a strong species endemism (54.1%) (Table 2). Presumably, due to its relative faunal paucity, the provincial division of the Indian Ocean has rarely been discussed [15]. Similarly, little is known about its Cenozoic brachiopod fossil record, rendering the tracing of the origin and distribution history of the brachiopod faunas throughout the ocean basin difficult [93]. Nevertheless, our cluster analysis clearly suggests the delineation of two possible bioprovinces: East Africa (C_1_) and Red Sea (C_2_). C_1_ (West Indian Ocean Bioprovince) extends from the offshore area of Somalia in the north, southward along the east coast of Africa, the seawaters around Madagascar, then turning west across the Cape of Good Hope (near Cape Town in South Africa) and then turning north until the coast of Namibia in southwest Africa. Despite this relative large coastal distance spread, the brachiopod faunas in this bioprovince seem to be closely related to each other, as indicated by the cluster analysis, and this biogeographical homogeneity may be explained by the transportation of Agulhas Current, which flows along the east and south coast of Africa (Fig 7; [93–95]).

Cluster analysis (Fig 5) also indicates that there is some faunal mixing (7 species, or 10% of C_1_ species and 37% of E_4_ species) between Southeast Africa and some South Indian Ocean islands (E_4_ in Fig 7) including Kerguelen, Crozet, and Prince Edward Islands. Nevertheless, this may well have risen as a result of the Agulhas Current passing through these islands especially since the Pliocene [96]. Moreover, due to the wide oceanic and climatic separation between the Southeast Africa coast and the South Indian Ocean islands, and also considering the biogeographical classification scheme of Spalding et al. [31], as will be discussed further below, we here tend to group the South Indian Ocean islands as an independent bioprovince within the Circumpolar Antarctic Bioregion (Bioregion E) (Table 3).

In comparison with C_1,_ C_2_ (Red Sea Bioprovince) is well defined and limited to the Red Sea. This bioprovince is thought to have become established after the Tethyan seaway was finally closed in the Miocene, blocking off faunal connections that had existed between the Mediterranean Sea and the Indo-Pacific oceans [66, 69, 97]. Currently, although seawater can exchange freely between the Red Sea and the Indian Ocean via a narrow and shallow strait (the Bab al-Mandab Strait) and the Gulf of Aden. Low diversity (12 species) and relatively high-endemism (33.3%) brachiopod fauna in the Red Sea, favouring its intermittent isolation in recent geological time [98–100].

#### Bioregion D (The Southwest Pacific Bioregion)

This bioregion is found in the south and eastern coast areas of Australia as well as around many of the islands in the southwest Pacific between New Zealand and New Guinea−Solomon Islands, straddling the Tropical, Warm-temperate and Cold-temperate climatic zones and largely corresponding to the areas influenced by the Eastern Australian Current (Fig 7). It is geographically close to and has a free seaway connection with Bioregions B and E, but according to the cluster analysis and the species endemism of this bioregion (43.6%, Table 2), the brachiopod faunal composition of D is distinct enough to be separate from these latter two regions (Fig 5).

Bioregion D comprises two distinct bioprovinces: the Southeast Australian Bioprovince (D_1_) and the New Caledonia−Fiji Bioprovince (D_2_). D_1_ extends from the coast of Queensland around 20°S to the coastal areas of South Australia, inclusive of the shelf waters of Tasmania. 33 living brachiopods are identified in this bioprovince, 12 of which (or 36.4%) are unique to this area. Common and characteristic taxa include genera *Anakinetica*, *Parakinetica*, and species *Murravia exarata*.

D_2_ (New Caledonia−Fiji Bioprovince) includes the shelf waters around New Caledonia, Fiji, Norfolk Island and Tonga Islands, and 55 living brachiopod species are reported, one-third of which (32.7%) are endemic to this bioprovince. Within this bioprovince, the greatest faunal affinity is identified among New Caledonia, Fiji, Norfolk Island and Tonga Islands (together they form one single cluster (cluster 19), but this may be a reflection of strong sampling and a positive study bias because the living brachiopod faunas of these islands have been the subject of a series of recent intensive systematic studies [101–107]. On the other hand, there is one grid cell from cluster 16 locating in this bioregion, but considering our criteria of bioregionalization mentioned before, it seems preferable to place this cell into D_1_, although in reality this ‘outlying’ grid cell may represent a form of an authentic disjunct distribution (as further discussed below).

In this study, the biogeographical identity of D_1_ is less well defined compared to D_2_. This is because, as shown by the cluster analysis and network analysis (Figs 5 and 6), D_1_ partially overlaps with several neighbouring bioprovinces; for example, it shares 10 species with D_2_, 10 species with B_2_, and 9 species with E_3_ (New Zealand). These overlaps suggest that the brachiopod fauna of eastern and southern Australia may, on the one hand, occupy an intermediate transition between the West Pacific (B_2_), the Southwest Pacific (D_2_) and New Zealand (E_3_) bioprovinces and, on the other, the challenge in realistically separating these bioprovinces. The close biogeographical similarity between southeast Australia and New Zealand has already been noted by Zezina [15] who grouped them under the same distributional type while classifying South Australia as belonging to a different type of distribution. However, considering the continuous distribution of Lingulidae, *Aulites* and *Basiliolella* across eastern Australia, New Caledonia and Fiji, concomitant with the extent of the strong and stable Eastern Australian Current connecting these areas, we suggest that eastern and southern Australia be better aligned with D_2_ to form the same bioregion, as indeed demonstrated by the cluster and network analyses (Figs 6 and 7). Although terebratellides are the dominant faunas in both Australia’s offshore waters and New Zealand, they are, however, represented by different subfamilies and in particular very different species assemblages, warranting them to be separated as two distinct bioregions. For example, the subfamily Anakineticinae are today common in Australian waters, but it had become extinct in New Zealand before the Miocene, where the Terebartellinae are instead dominating [11]. In addition, the lack of lingulide brachiopods in New Zealand also supports its distinction as a separate bioregion.

#### Bioregion E (The Circumpolar Antarctic Bioregion)

Geographically, this is a large and widely dispersed grouping, not only encompassing the coastal waters of Antarctica but also the southern part of South America, New Zealand as well as some other smaller islands in the Southern Indian Ocean (Fig 7). Climatically, this bioregion is limited to the southern Polar and Cold-temperate zones. Despite its high latitudinal position and generally cold-water conditions, this bioregion contains a large number of species (85) marked by a strong degree of species endemism (52.9%). However, it should be noted that the spatial distribution of its species richness is highly variable, with most of the species being concentrated in the New Zealand bioprovince (Table 2).

Both cluster and network analyses indicate the presence of four distinct bioprovinces within the Circumpolar Antarctic Bioregion; they are here named Antarctica (E_1_), Southern America (E_2_), New Zealand (E_3_) and South Indian Ocean (E_4_). The sea area around the Antarctic continent is grouped into E_1_. This is a robust bioprovince represented by one highly cohesive cluster (cluster 6, Fig 5B), and is the only bioprovince located in the Southern Polar Zone in the Circumpolar Antarctic Bioregion and characterized by the genus *Compsothyris* and a relatively low level of species endemism (17.9%).

E_2_ (Southern American Bioprovince) comprises the coastal waters on both sides of southern South America, including Peru in the west and Argentina in the east; it also encompasses the seas surrounding the South Shetland Island (near the Antarctic Peninsula), South Sandwich Islands and Falkland Islands. As such, this bioprovince is probably the same as the ‘South American type’ of distribution noted by Zezina [15] (Table 3). Climatically, the bioprovince reaches into both the Warm- and Cold-temperate zones on both sides of southern South America, with its northern boundary set at approximately 30°~35° S where the Peru Current in the west and Falkland Current in the east are located. In the south of E_2_, there exists some faunal overlapping with E_1_ around the Scotia Sea. Based on our cluster analysis (Fig 5A), Cluster 6 (corresponding to E_1_) and Cluster 10 (corresponding to E_2_) share 15 of the 28 species present in both of the E_1_ and E_2_ bioprovinces (Table 2), pointing to a strong degree of biogeographical connection between southern South America and the Antarctic Peninsula. Indeed, common taxa living in E_1_ and E_2_ have broad bathymetric ranges, suggesting that shallow water connection is not necessarily a requirement for biogeographical communication between bioprovinces or bioregions [101]. In fact, the biogeographical connection between southern South America and the Antarctic Peninsula can be traced back to at least the Oligocene or Miocene [108] (e.g., through the distribution of the fossil species *Terebratella dorsata*), and the sustained connections are believed to have been facilitated and maintained by the existence of the Scotia Arc, a series of continental fragments and separated islands [109]. This arc would have and continue to serve as a corridor of marine biogeographical dispersal stepping stones between southern South America and the Antarctica [110, 111].

E_3_ (New Zealand Bioprovince) encircles the North and South Islands of New Zealand, as well as the Chatham Island and Auckland Island areas. Though located geographically close to D_1_ (east and Southeast Australia) and D_2_ (Southwest Pacific islands), cluster analysis and comparison of faunal composition indicate that there are more common species shared between E_3_ and E_1_ (13 common species) than it is between E_3_ and D_1_ (9 common species), suggesting that the New Zealand brachiopod fauna as a whole is biogeographically closer to those of Antarctica. This link, we believe, can be explained by the combination of (1) the more southerly position of New Zealand (mostly within the Cold-temperate zone) when compared to Australia and the Southwest Pacific, (2) strong and sustained influence of the Antarctic Circumpolar Current, and (3) the intermediate depth (mostly <1000 m deep) of the Campbell Plateau between New Zealand and Antarctica—thus providing a possible faunal migration pathway between E_1_ and E_3_. The isolation of New Zealand from its neighbouring bioprovinces (D_1_, D_2_ and E_1_), by both the Tasman Sea reaching >2000 km wide, South Fiji Basin (>1000 km) and the Sub-Antarctic Front [112], can also add to the explanation of why the New Zealand bioprovince has exhibiting a relatively high level of species endemism (30.9%) in bioprovince level (Table 2).

E_4_ (South Indian Ocean Bioprovince) consists of the areas around the Prince Edward Islands, the Crozet Islands and the Kerguelen Islands, all located in the Southern Indian Ocean between Antarctica and southern Africa. The range of the E_4_ bioprovince is consistent with the ‘Kerguelen type’ of distribution as proposed by Zezina [15] (Table 3). Notably, this provinces includes grid cells that also demonstrate some compositional similarities with A_2_ and E_3_ (Fig 5B) in our initial cluster analysis when the K value was set at 20, but when we selected K = 29, the two outlying grid cells in New Zealand can be separated (S6 Fig). E_4_ has the lowest endemism (15.8%) among all the bioprovinces concerned in this study, despite its relatively high family richness (12) when compared to E_1_ and E_2_. This disproportionately low species endemism is perhaps not surprising given the province’s oceanic setting and remoteness. Instead of acting as centres of speciation, the oceanic islands of E_4_ are more likely to have acted as centres of species accumulation or biogeographical stepping stones where species originated elsewhere descend to and blend, thus giving a low endemism appearance characterized by mixing of the elements from distinct neighbouring bioprovinces (e.g., [113]). Therefore, in the case of E_4_, even though cluster analysis indicates its close relationship to A_2_, its geographical proximity with the Antarctica, its location with the southern Cool-temperate zone and, especially, its possession of several well-known Antarctic terebratellide elements (e.g., *Magellania* and *Aerothyris*, see [114]) would support its stronger biogeographical affinity with the Circumpolar Antarctic Bioregion.

To summarize, the Circumpolar Antarctic Bioregion (Bioregion E) can be characterized as a robust and cohesive biogeographical entity. Although distinct bioprovinces are recognized within the bioregion as described above, there are no land barriers between these bioprovinces, as attested by the free and continuous distribution of *Liothyrella* in all four bioprovinces, *Aerothyris* in E_3_ and E_4_, and *Terebratella* in E_2_ and E_4_. The shared presence of these genera likely reflects the significance of the Antarctic Circumpolar Current acting as a major biogeographical dispersal agent connecting and facilitating the faunal exchanges between these bioprovinces. Another potentially significant, but yet poorly documented, biogeographical dispersal mechanism for the Circumpolar Antarctica Bioregion is via scattered islands and moderately deep seamounts located along the oceanic ridges/rises, with them acting as biogeographical stepping stones for the dispersal of brachiopods [115]. Still another possible dispersal agent and mechanism, as far as living Antarctic brachiopods are concerned, is the West Wind Drift zone (Antarctic Circumpolar Current). It is likely that the common distribution and exchange of such brachiopod genera as *Aerothyris* and *Liothyrella* among the E_1_, E_3_ and E_4_ bioprovinces have been made possible by this drift, as already suggested by Foster [108], since these bioprovinces are separated by deeper water barriers.

#### Other possible independent bioprovinces

Hawaii (Hawaiian chain of islands emerged 29~23 Ma, [116]), Galápagos (oceanic crust built <10 Ma, [117]) and Amsterdam-St Paul (flat-topped seamounts formed 8~10 Ma, [118]) are volcanic islands, have relatively smaller areas than other identified bioprovinces, and contain fewer species, lower endemism and some of the lowest diversification rates (Table 2). While their relative young geological ages, compared to continents, may explain much of their peculiar biogeographical features, poor sampling combined with lack of systematic studies may also have played a role. It is therefore important to note that the biogeographical identity of these volcanic islands, though here below temporarily treated as representing independent bioprovinces, is certainly subject to further studies.

Based on the cluster analysis, Hawaii Islands are closely linked to the West Pacific Bioprovince (B_2_) (Fig 5); for example, they share a number of Pacific elements like *Lingula* and *Frenulina* [11, 119]. However, due to its remote location, with >3,000 km water separation from the nearest continent shelf, and no major ocean currents passing nearby, it is difficult to group these islands with B_2_ or any other bioprovinces, we are therefore inclined to consider Hawaii as an independent bioprovince.

In Fig 5A, cluster analysis seems to suggest a grouping membership of Galápagos with E_2_. Whilst this link may be explained by the influence of the cold-water Peru Current acting as a dispersal agent bringing some elements (e.g., *Liothyrella*) of the temperate E_2_ bioprovince to Galápagos species, it is difficult to conceive a single united brachiopod bioprovince stretching three climatic zones (Tropical, Warm- and Cold-temperate) on the western side of South America, especially when little is known about the living brachiopods connection between Galápagos and southern South America.

Regarding the Amsterdam-St Paul islands, it has only yielded seven living brachiopod species in seven genera, suggesting an extremely low species diversification rate, but one that is consistent with its very young geological age. As such, its biogeographical identity is difficult to ascertain. It could be grouped with other nearby South Indian Ocean islands under E_4_ (sharing *Pemphixina pyxidata* and *Liothyrella winteri* in common species), or be treated as an independent biogeographical unit in respect of its remote oceanic location, geographical separation, its warmer temperature setting compared to E_4_ and, more importantly, its relative high species endemism (28.6%, unique brachiopod species include *Striarina valdiviae* and *Megerlina davidsoni*, [114]). Both Zezina [15] and Spalding et al. [31] have regarded Amsterdam-St Paul as a separate biogeographical unit (Table 3).

### Ocean currents and living brachiopod biogeography

Zezina [12, 13, 15] had repeatedly stated that ocean currents play a significant role in the distribution and bioregionalization patterns of living brachiopods. This view is confirmed in the present study and may be expanded further in three different aspects. First, ocean currents initiate and facilitate the exchange of species between different bioregions/bioprovinces. In the case of living brachiopods, irrespective of their larval types (lingulides and discinids attain planktotrophic larvae while rhynchonelliform and craniiform brachiopods usually contain non-feeding lecithotrophic larvae, see [120, 121]), their distribution during the time spent as planktonic organisms is greatly enhanced by the continuous flow of surface ocean currents. The impact of ocean gyres on the formation of brachiopod bioregions appears so pervasive that geographically widely separated locations might be united into the same bioregion, as in the case of the North Atlantic Bioregion. In this example, the influence of the North Atlantic Gyre clearly has surpassed the potential impacts of climate zones, geographical distance and even ocean separation, because under the influence of this gyre, there is a great degree of unity in living brachiopod faunas between the two sides of the Atlantic Ocean despite the great expanse between them. A similar situation also occurs in the North Pacific, where the North Pacific Gyre links three bioprovinces B_1_, B_2_ and B_3_ together with close faunal similarities (Fig 7). Analogously, the role of the West Wind Drift in sourcing and sustaining the Circumpolar Antarctic Bioregion may also be compared, further highlighting the significance of large-scale ocean currents (gyres) in configuring and limiting brachiopod bioregions, the first-order bioregionalization unit at the global scale.

Apart from ocean gyres, it is also evident that some brachiopod bioprovinces may be more closely configured by smaller-scale regional ocean currents as they carry and spread warm or cold waters to more adjacent habitat areas across latitudes. For example, due to the Norwegian Current, a regional warm-water current derived from the North Atlantic Gyre, that conveys warm water to Subarctic Norway, extends the border of the A_1_ bioprovince as far north as to the northern coast of Norway (Fig 7), and is thought to have introduced *Terebratulina retusa* to a wider distribution area and higher latitudes [60]. Likewise, in bioprovince E_2_, the cool-water Peru Current flowing northward off the Chilean coast is believed to have aided the wide distribution of *Magellania* and *Terebratella* to reach around 30°S on the west coast of South America [122].

Another very interesting example of brachiopod bioregionalization at the bioprovince level through the influence of regional ocean currents is the observation of biogeographical transitional zones (biogeographical ecotones) where the brachiopod fauna is characterized by containing a significant mixture of elements from adjacent bioprovinces. One such example has been identified in the coastal areas of Japan, between two other well-defined independent bioprovinces (B_1_ and B_2_ in Fig 7). As already discussed before, the formation of this transitional bioprovince can be evidently linked to the intermingling of both warm-water and cold-water currents in this region.

### Upwelling systems and living brachiopod biogeography

The role of world major coastal upwelling systems in affecting the species richness and distribution patterns of living brachiopods have been mentioned by Zezina [13, 123]. In particular, Zezina observed that coastal upwelling ecosystems do not provide favourable conditions for living brachiopods, especially rhynchonelliformean brachiopods that feed on dissolved organic matters (as lecithotrophic feeders), due to their unusual high load of nutrients (especially phytoplankton) and thus the high risk of eutrophication and hypoxia. Zezina’s conclusion in this regard is supported by our study. As shown in Fig 4, none of the global four major upwelling systems (Peru-Chile, California, Canary and Benguela upwelling systems) have been identified as major centres of species richness; in fact, all but the Canary upwelling system off Northwest Africa and the Californian upwelling system in southwest United States have produced sufficient numbers of living brachiopods for them to be included in the present study (Fig 4). The paucity of brachiopods occurring in upwelling systems is not considered as the product of possible under sampling because species-poor but high-abundance brachiopod assemblages, typically dominated by planktotrophic-feeding linguliformean brachiopods, have been reported from these ecosystems. As noted by Zezina [13, 123], brachiopods from the upwelling zones are characteristic of benthos typically found in the Oxygen Minimum Zones (OMZs) of world oceans; generally they have low species richness, high abundance (high dominance, e.g., high population density of rhynchonelliform brachiopods, [93, 124, 125]), small body-sized, and thin-shelled. Two excellent examples of upwelling-adapted living brachiopod faunas are known: one from the coast off Southeast Brazil [124] where a collection of 12,733 specimens was found but have only yielded four species; and the other from the coast of California [126] where most of the sampling sites reported only one densely populated single species.

The rarity of living brachiopods in the coastal upwelling systems located on the eastern sides of world major oceans stands in stark contrast to brachiopod bioprovinces located in similar latitudes but on the western side of these oceans. This remarkable brachiopod biogeographical phenomenon has been noted by Zezina [13, 15] who had described this feature as a peculiar meridional asymmetry in the global distribution of living brachiopods. This highlights the fact that upwelling ecosystems not only suppress the diversification of most brachiopods in these ecosystems, they also seem to contribute little to the global dispersal of living brachiopods.

### Biological factors and biogeographical comparisons

In addition to the abiotic environmental factors mentioned above that influence geographical distribution of living brachiopods, biotic interactions with other organisms such as predation and competition for resources potentially may also influence the biogeographical patterns of living brachiopods. However, these biological factors usually operate at a much smaller (mostly local) scale, therefore they are unlikely to have significantly influenced the global bioregionalization patterns of living brachiopods recognized in this paper. Besides, the majority of living brachiopod species included in our dataset are from shallow waters (continental shelf, coral reefs, subtidal settings) where the brachiopods tend to dwell in cryptic habitats and rocky substrate (e.g., rocky/reef cavities, caverns, caves), away from potential competitors and/or predators. In these habitats, we do not have enough evidence concerning the direct biotic interactions between living brachiopods and their potential competitors.

The observation we made above that the bioregionalization pattern of living brachiopods has not been substantially influenced by other major marine organism groups is corroborated by the broad similarities in global biogeographical patterns between living brachiopods and other major marine organisms. For example, a number of common biogeographical elements are found between the global bioregionalization framework of living brachiopods revealed in this paper and that of Spalding et al [31], the latter being based on a meta-analysis of existing global knowledge concerning the biogeographical patterns of benthic and pelagic biotas in coastal and shelf waters (see Table 2 for the comparisons). At a more focused taxonomic level, we also see considerable similarities between the bioregionalization pattern of brachiopods here recognized and that for living bivalves [28]. Bivalves are predominant marine benthos in present-day marine ecosystems, and their global species richness far exceeds that of living brachiopods − this is despite the fact that they coexisted and often shared similar habitats with brachiopods throughout the Phanerozoic [23, 127]. In this comparison (see Table 2), although more provinces were identified for bivalves, probably reflecting much better sampling and more extensive studies of this group compared to living brachiopods, it is noteworthy that some bivalve provinces overlap to a certain degree with brachiopod provinces. For example, the Japan Sea and California coast areas, located in similar latitudes, are both subdivisible into smaller bioprovinces for both brachiopods and bivalves ([28] and this study). Despite the similarity, however, some small differences exist between the brachiopod and bivalve provinces, as exemplified by the slightly more northly located boundary of the brachiopod bioprovince in the Japan Sea compared to the boundary of the bivalve bioprovince (boundary between B_1_ and B_2_ in this study; boundary between Warm Japonic and Cool Japonic in [28]). Whether this offset is due to their mutual competition for space and resources requires further studies.

As aforementioned, biological factors such as predation and competition may not have exerted substantial influence on the global biogeographical patterns of living brachiopods, but the well-known latitudinal gradient of marine predation pressure [e.g., 128–130] potentially may bear on one of the outcomes revealed in this study, that is, living brachiopods tend to be most diverse in mid-latitude temperate waters of both hemispheres (Figs 2 and 3), shunning from the warm equatorial areas where predation pressure is believed to be the highest. How likely this scenario can be supported by the living brachiopod data requires careful further investigations as the latitudinal biodiversity gradient is known to be related to an array of diverse environmental, ecological, biological and evolutionary factors, including predation as both an ecological and evolutionary selection pressure [e.g., 131]. This is certainly a related topic but falls outside of the scope of the present study, and will be investigated in a planned future study.

### Challenges of sampling and disjunct distributions

Typical of this kind of global dataset-based study is the unavoidable challenge of sampling bias and data deficiency. As already stated, despite our great and explicit effort to minimise the impacts of biased sampling on the integrity of our data and analysis, it is still inevitable that there are some caveats associated with our data. For example, approximately 30% of the total analysed data are from museums, expeditions or surveyor voyages (downloaded from GBIF and OBIS), where often there was no control of brachiopod taxonomy by specialists. Another challenge is the concentration of available data points in certain geographical areas (e.g., North America, Europe, Australia and New Zealand, [132]), while other regions, particularly tropical and deep-sea areas are characterized by disproportionately under-sampling and thus have poor data representation (rarefaction analyses in Fig 1 shows clearly insufficient sampling in low latitude areas). All these, however, do not obscure the fact that the bulk of our data is from trustworthy literature sources. More notably, we did not take the data at their face value; rather, as we mentioned before, each taxonomic attribution and occurrence were critically evaluated. Such a ‘verification by an expert’ approach is a critical step often adopted for global biogeographical studies involving large datasets from diverse sources, akin to our study [e.g., 31]; this approach indeed provided additional confidence and integrity to the final dataset we used for the analysis.

Nevertheless, it is necessary to note the challenges and caveats associated with the interpretation of a few specific clusters and their biogeographical affinities. As already referred to before, clusters 1, 2, 5, 11, 16 in Fig 5 did not form a cohesive group among themselves nor did they unequivocally constitute members of any other well-defined groups. This was caused by these clusters each containing grid cells that are geographically separated so far apart but somehow weakly linked together due to their sharing of a low number of species. There may be two reasons explaining this peculiarity: these clusters were either under sampled, or they contain some species of genuine biogeographical disjunct distributions; or a combination of both of these factors.

First, there is good evidence to indicate poor sampling for all these five clusters, judging from the average number of species and grid cells contained in these clusters in comparison with the average of all the 20 clusters included in our cluster analysis: the average of species per cluster among these five clusters is 22.2, much smaller than the average of 36.3 species per cluster for all the 20 clusters, and the average number of grid cells per cluster for these five clusters is 3.8, much lower than the same average of 9 for all the 20 clusters. It is well known that unequal sample sizes present a significant challenge for cluster analysis as it could lead to inappropriate clustering outcomes [133]. For example, cluster 1 contains three 5° grid cells (Hawaii; North Maluku, Indonesia; Coral Sea), all located in the Pacific Ocean. Though they were grouped together in one cluster in our initial cluster analysis (S3 Fig), there is only one species (*Lingula rostrum*) shared among the three grid cells, and this species is widely distributed in western Pacific as well as in the Indian Ocean. Therefore, it would be inappropriate to regard this cluster as forming a cohesive independent and spatially continuous bioprovince; instead, as already discussed, the biogeographical affinity of these three cells should be assessed individually and be aligned to an appropriate bioprovince accordingly (Fig 7), with due consideration given to the impact of sampling bias.

Second, the challenge in assigning these five clusters to a particular well defined group may also have been compounded by the fact that some or all of these clusters contain species whose global distributions are genuinely disjunct, meaning that their natural spatial distributions are discontinuous and patchy, especially for the species with lecithotrophic larvae. Therefore, the forceful grouping of samples (i.e., grid cells or clusters in our case) executed by cluster analysis must be interpreted with caution. For instance, two grid cells each located in a high latitude area of each hemisphere could be grouped together by cluster analysis due to their sharing of one or more antitropical or bipolar species (see [134] for definition of antitropical or bipolar distribution). In this case, it would be inappropriate to regard these two cells to form an independent and cohesive bioprovince or together belong to another existing province simply because of their shared possession of some antitropical or bipolar species. As noted in many previous studies [e.g., 134–137], antitropical and bipolar distributions are common in both modern and past ecosystems; they represent one type of genuine disjunct distributions that should be explained as representing separate biogeographical entities that share some taxa because of long-distance dispersal or geographical vicariance, rather than being interpreted as forming a single seemingly cohesive biogeographical entity. In our study, cluster 2 seems to be a good case exemplifying this scenario. This cluster contains four 5° grid cells, all of which share two lingulide species: *Lingula anatina* and *Lingula adamsi*. The former is a cosmopolitan species, with distributions extending from western Pacific westward through the Indian Ocean to eastern Atlantic. By contrast, *Lingula adamsi* seems to be an antitropical (or bitemperate) species with disjunct occurrences in East China Sea and northeastern Australia. As such, it would be inappropriate to regard cluster 2 as constituting a bioprovince of its own. Instead, we argue that the two disjunct grid cells should be, respectively, more closely aligned with their nearest bioprovince, as shown in Fig 7. Similarly, the same scenario can be invoked to explain why the three disparate grid cells of cluster 16 (South China Sea, East Australia, North Madagascar) should not be considered as representing a single spatially continuous bioprovince even though they all contain *Nipponithyris afra* [138]. The global distribution of *N*. *afra* is clearly disjunct and antitropical, and therefore cannot be used alone as an indicator of biogeographical grouping. On the other hand, South China Sea also contains records of *Acanthobasiliola*, a genus restricted to the Western Pacific−Indo-Malayan Archipelago Bioprovince (B_2_), hence supporting a close biogeographical affinity between the South China Sea grid cell and B_2_. Likewise, even though one grid cell each in eastern Australia and Madagascar was included in cluster 16, each of these grid cells contains their own unique species. For example, *Aulites brazieri* is one of the most common species in eastern Australia, pointing to a strong relationship between this grid cell and D_1_. Similarly, the grid cell of cluster 16 in North Madagascar contains four species (*Lacazella mauritiana*, *Megerlina pisum*, *Megerlia acrura*, *Basiliola arnaudi*) that are completely endemic to C_1_, underscoring their strong mutual biogeographical affinities.

Similar accounts can also be made about clusters 5 and 11, but in these two cases, the challenge in interpreting their provinciality based on the cluster analysis is more centered around species with very broad bathymetric ranges or teleplanic distributions that are typically associated with small remote and disparate oceanic islands [139]. For example, cluster 5 contains six 5° widely scattered grid cells even though there are no species uniquely common to any of the grid cells. On the other hand, the cluster possesses three spatially disjunct and bathymetrically wide-ranging species (*Pelagodiscus atlanticus*, 3,000~6,000 m in bathymetric range [120]; *Novocrania lecointei*, extending from continental shelf to bathyal slopes [120]; and *Neorhynchia strebeli* strictly in the abyssal zone [14]), all of which may be considered as teleplanic taxa that are able to and indeed have achieved very wide oceanic distributions, presumably either via long-distance dispersal or by taking advantage of mid-ocean ridges, seamounts and submarine ridges as dispersal stepping stones [140], or for some possibly even via deepsea hydrothermal vents [141]. Regarding cluster 11, it comprises three widely separated grid cells, two from the northern part of the Red Sea [97] and one from southwest Madagascar [58]. Likely, these grid cells were artificially clustered together because of their shared presence of one wide-ranging species (*Thecidellina blochmanni*) and their small sample sizes. Instead, in this study, we have treated the grid cells from the Red Sea as an independent bioprovince (C_2_), and the cell from near Madagascar as being part of the West Indian Ocean Bioprovince (C_1_) (Fig 7).

As a final note, we draw a comparison between the bioregionalization framework of living brachiopods erected in this study with the scheme created by Zezina [15]. Although the two studies were based on independent datasets, analyzed at different spatial scales and by very different approaches (Zenina’s study was based mostly on her own expert knowledge and her own dataset, compared to this study which is based on a globally compiled dataset from literature sources combined with a critical and expert-informed review) and methods (qualitative in Zezina’s work, compared to a quantitative approach used in the present study), but yet they have striking similarities with respect to the number of major brachiopod biogeographical regions and provinces. Some differences exist and these have been explained in detail in the discussion section. This high fidelity of the present study in reproducing (and thus confirming) Zezina’s scheme [15] is an important point to note as it strongly reflects that the dataset we used for this analysis is sufficiently robust to reveal the global patterns of living brachiopod distribution. Additionally, it should also be noted that the bio-distribution of living brachiopods is a three-dimensional dynamic process [18, 22], involving space, time and morphology. In this article, however, the main intention is to elaborate a quantitative and generalized framework to reveal the global bioregionalization of living brachiopod based on all the data accessible to us. Importantly, the dataset we have compiled for this study provides a baseline for future, more in-depth studies that will further elucidate the underlying mechanisms and environmental factors that govern the bio-distribution of living brachiopods.

## Conclusions

Based on a newly compiled dataset of 14918 geo-referenced occurrences from 394 living brachiopod species worldwide, we first mapped the global distribution of living brachiopod species and genera in 5° latitude-longitude grid cells, which enabled the visualization and identification of distinct biogeographical entities and biodiversity hotspot areas. Further investigation of the dataset using cluster and network analyses allowed us to propose the first systematically and quantitatively recognized global bioregionalization framework for living brachiopods, consisting of five bioregions and thirteen bioprovinces, plus three small independent bioprovinces.

No single environmental or ecological variable could be recognized to explain the newly proposed global bioregionalization patterns of living brachiopods. Instead, the combined effects of large-scale ocean gyres, climatic zonation as well as some geohistorical factors (e.g., formation of land bridges and geologically recent closure of ancient seaways, both leading to the formation or demise of major geographical barriers) are considered as the main drivers at the global scale. At the regional scale, however, the faunal composition, diversity and biogeographical differentiation appear to be mainly controlled by seawater temperature variation, regional ocean currents and coastal upwelling systems.

Insufficient sampling and genuine disjunct biogeographical distributions (e.g. antitropical, bipolar or the general teleplanic distributions) may be responsible for the difficulty in interpreting the biogeographical affinities of a few clusters, highlighting the need to take caution in analysing and interpreting large-scale heterogeneous datasets involving certain multivariate techniques such as cluster analysis.

## Supporting information

S1 Fig Relationship between species and continental shelf area along latitudes in global scale and in different ocean coasts. (TIF)

S2 Fig Methods of determining the number of clusters. (a) “Elbow method”, plot of total within sum of squares against the number of clusters to determine the optimal number of clusters; (b) “Silhouette Method” plot of average of silhouette with against the number of clusters to determine the optimal number of clusters, it seems both k = 20, 23, 26, 29 are rational values for cluster analyses.
(TIF)

S3 Fig Dendrogram of cluster analysis showing twenty clusters when we selected K = 20. (TIF)

S4 Fig Dendrogram of cluster analysis and map showing twenty clusters when we selected K = 23. (a) Dendrogram of cluster analysis when K = 23; (b) the numbers of each cell are corresponding to the above dendrogram figure, the colors in Global Map are corresponding to the bioregions/bioprovinces in Fig 7. Asterisk indicates the outliers, means that even the cells are distributing very far from other cells from the same cluster during the cluster analysis. Source: global basic map was downloaded from ArcWorld Supplement via ESRI and [52]), then adapted for visualization here by using open source Geographic Information System QGIS (http://qgis.osgeo.org).
(TIF)

S5 Fig Dendrogram of cluster analysis and map showing twenty clusters when we selected K = 26. (a) Dendrogram of cluster analysis when K = 26; (b) the numbers of each cell are corresponding to the above dendrogram figure, the colors in Global Map are corresponding to the bioregions/bioprovinces in Fig 7. Asterisk indicates the outliers, means that even the cells are distributing very far from other cells from the same cluster during the cluster analysis. Source: global basic map was downloaded from ArcWorld Supplement via ESRI and [52]), then adapted for visualization here by using open source Geographic Information System QGIS (http://qgis.osgeo.org).
(TIF)

S6 Fig Dendrogram of cluster analysis and map showing twenty clusters when we selected K = 29. (a) Dendrogram of cluster analysis when K = 29; (b) the numbers of each cell are corresponding to the above dendrogram figure, the colors in Global Map are corresponding to the bioregions/bioprovinces in Fig 7. Asterisk indicates the outliers, means that even the cells are distributing very far from other cells from the same cluster during the cluster analysis. Source: global basic map was downloaded from ArcWorld Supplement via ESRI and [52]), then adapted for visualization here by using open source Geographic Information System QGIS (http://qgis.osgeo.org).
(TIF)

S1 Table List of living brachiopod species, only geo-referenced occurrences and analysed species included. (DOCX)

S2 Table All available literature included in this research. (DOCX)

S3 Table Jaccard similarity coefficient matrix comparing 20 different clusters. (DOCX)

S4 Table Jaccard similarity coefficient matrix comparing seven different groupings. (DOCX)
