# Supplementary figures and images for: Global biogeography of living brachiopods: Bioregionalization patterns and possible controls
Source: PLoS One. 2021 Nov 8;16(11):e0259004. doi: 10.1371/journal.pone.0259004 (PMC8575269; doi:10.1371/journal.pone.0259004)

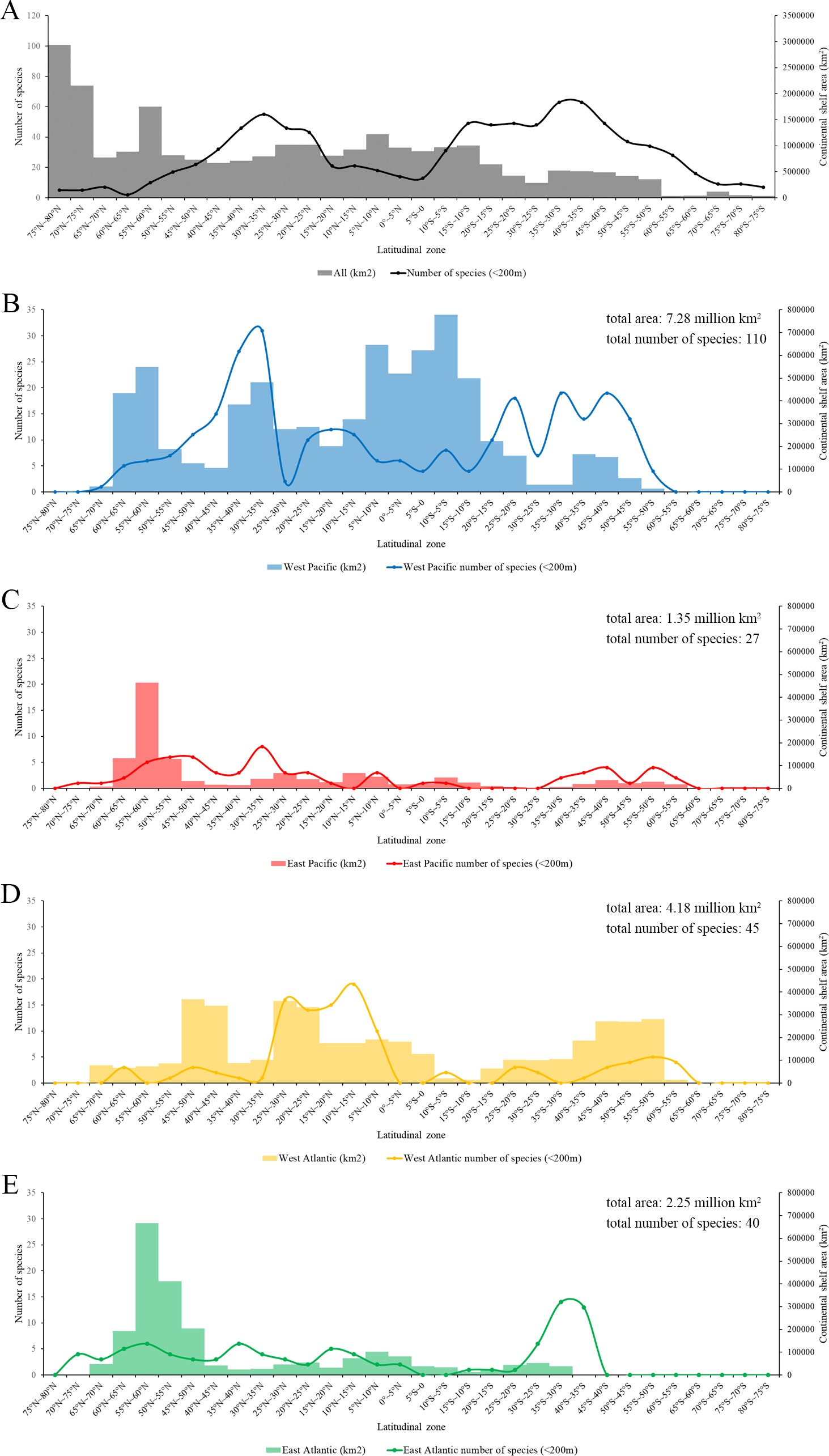

Supplement: S1 Fig — (TIF) [file pone.0259004.s001.tif]

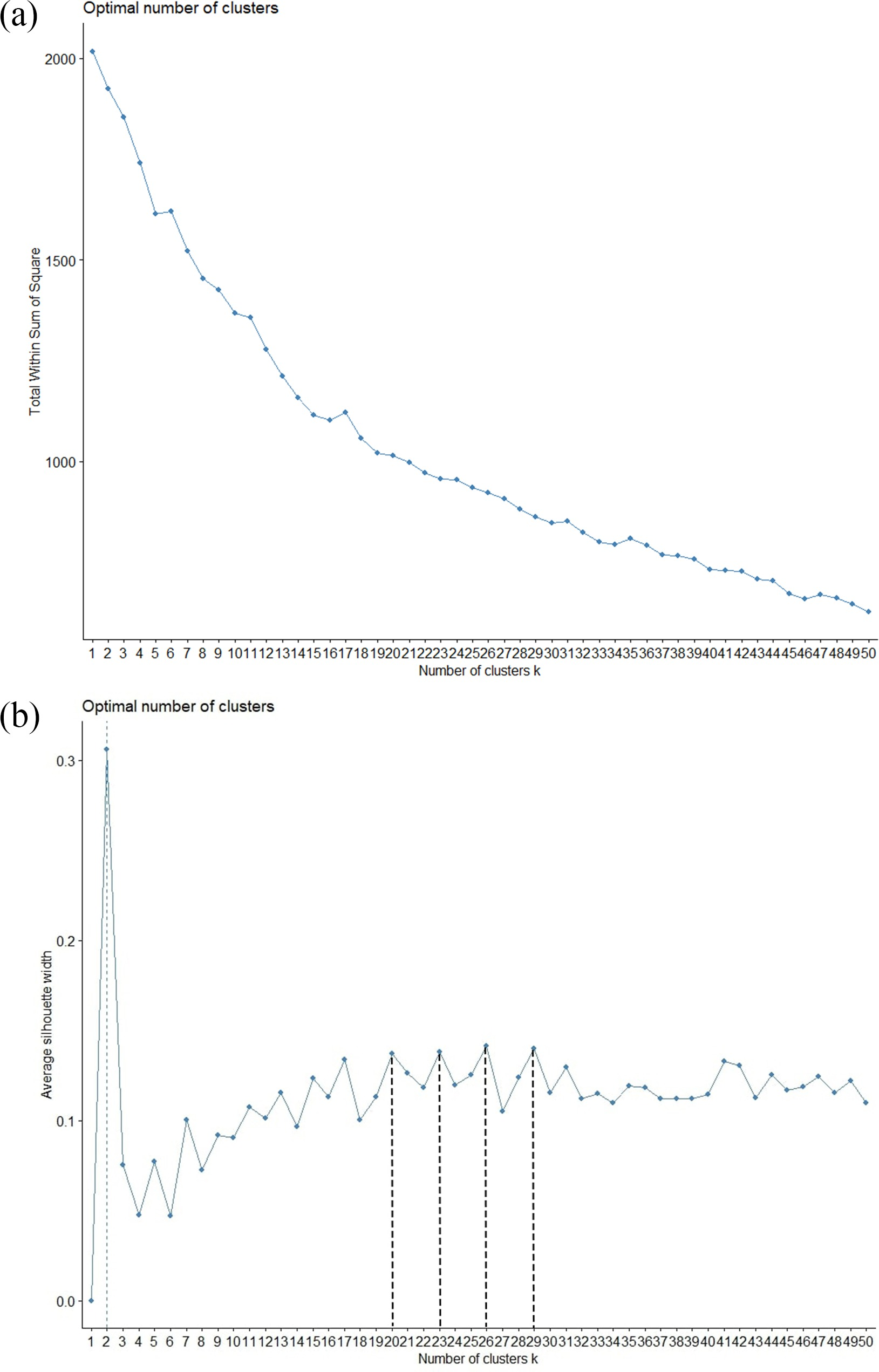

Supplement: S2 Fig — (a) “Elbow method”, plot of total within sum of squares against the number of clusters to determine the optimal number of clusters; (b) “Silhouette Method” plot of average of silhouette with against the number of clusters to determine the optimal number of clusters, it seems both k = 20, 23, 26, 29 are rational values for cluster analyses. (TIF) [file pone.0259004.s002.tif]

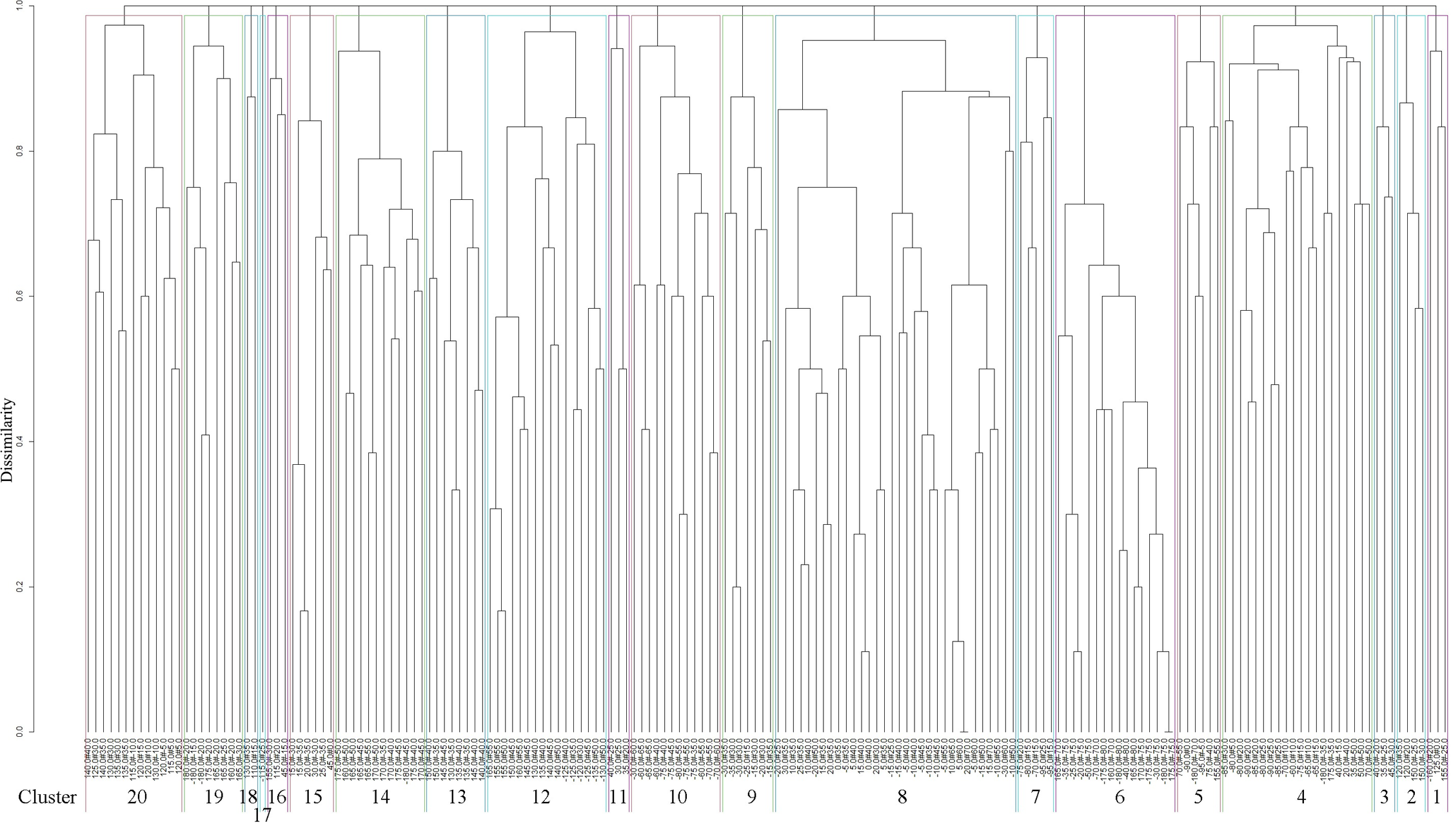

Supplement: S3 Fig — (TIF) [file pone.0259004.s003.tif]

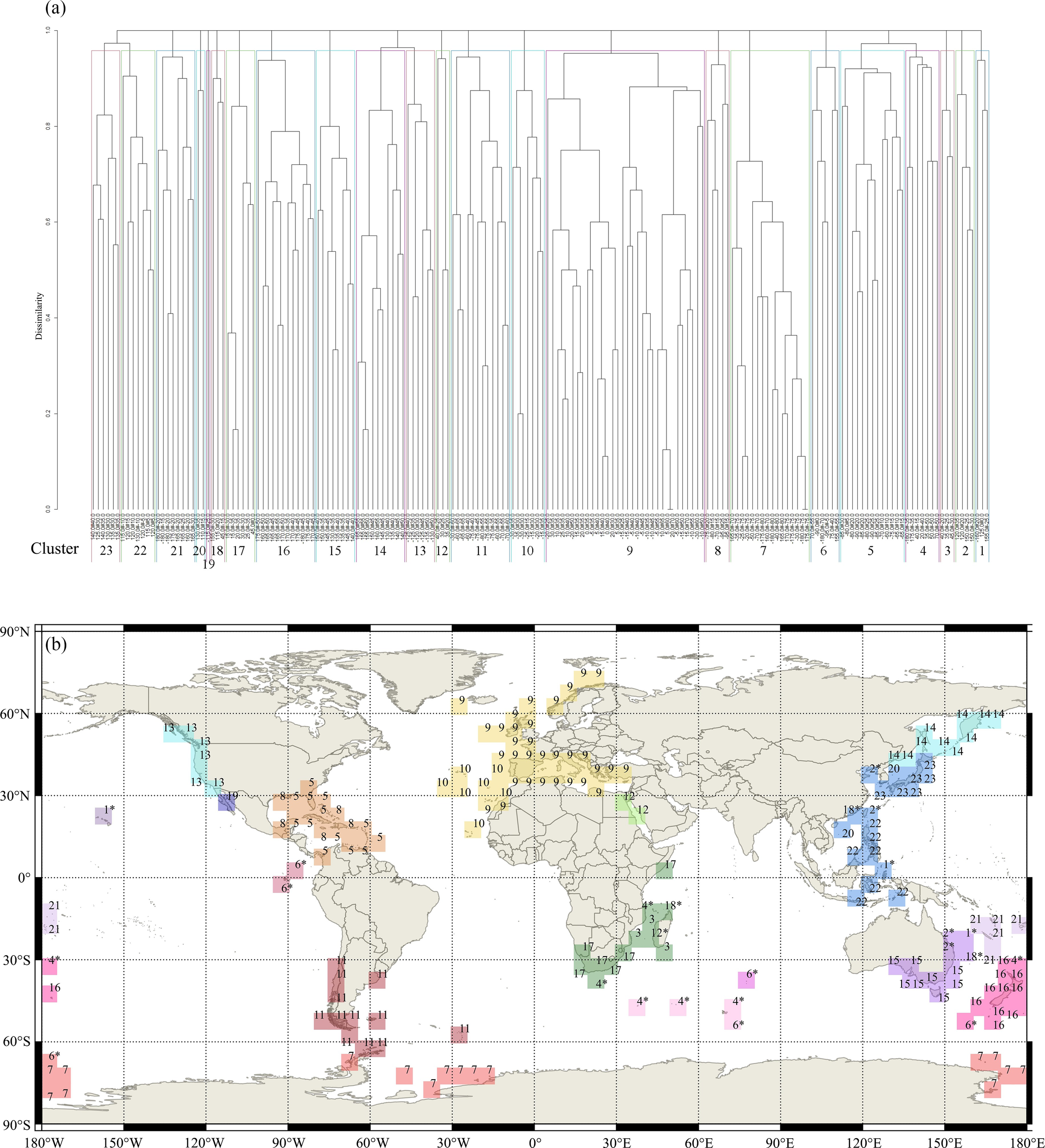

Supplement: S4 Fig — (a) Dendrogram of cluster analysis when K = 23; (b) the numbers of each cell are corresponding to the above dendrogram figure, the colors in Global Map are corresponding to the bioregions/bioprovinces in Fig 7. Asterisk indicates the outliers, means that even the cells are distributing very far from other cells from the same cluster during the cluster analysis. Source: global basic map was downloaded from ArcWorld Supplement via ESRI and [52]), then adapted for visualization here by using open source Geographic Information System QGIS (http://qgis.osgeo.org). (TIF) [file pone.0259004.s004.tif]

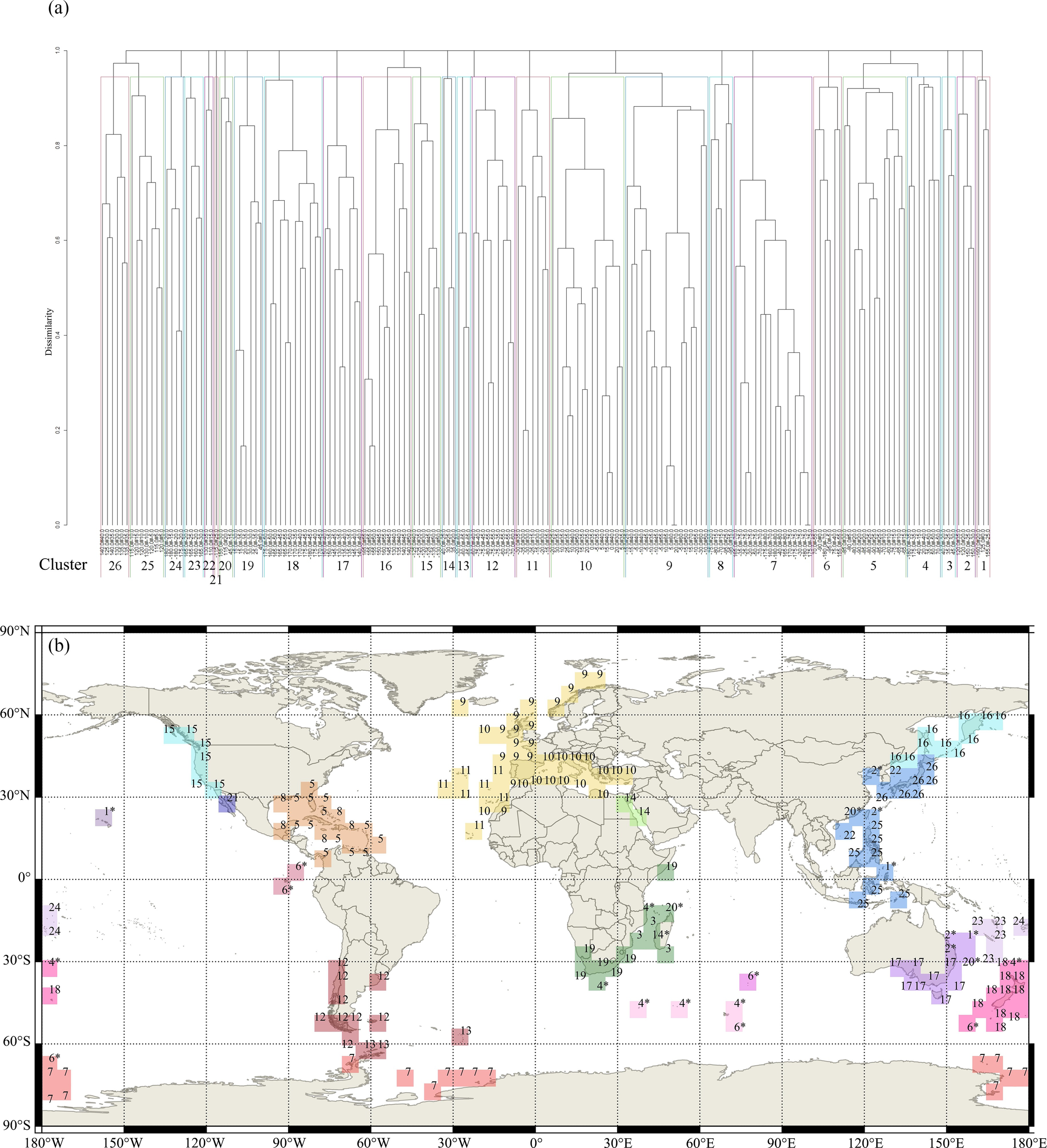

Supplement: S5 Fig — (a) Dendrogram of cluster analysis when K = 26; (b) the numbers of each cell are corresponding to the above dendrogram figure, the colors in Global Map are corresponding to the bioregions/bioprovinces in Fig 7. Asterisk indicates the outliers, means that even the cells are distributing very far from other cells from the same cluster during the cluster analysis. Source: global basic map was downloaded from ArcWorld Supplement via ESRI and [52]), then adapted for visualization here by using open source Geographic Information System QGIS (http://qgis.osgeo.org). (TIF) [file pone.0259004.s005.tif]

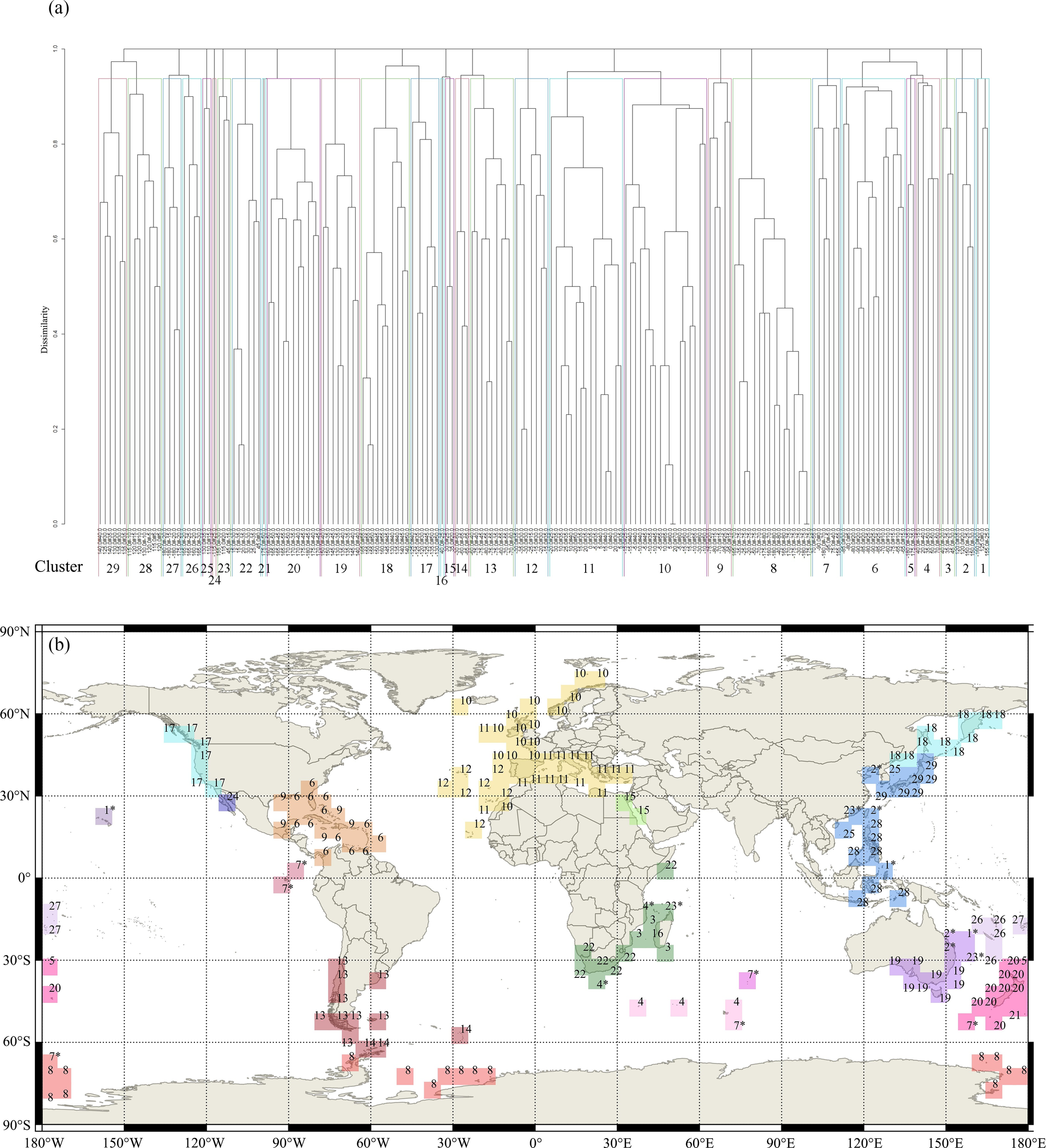

Supplement: S6 Fig — (a) Dendrogram of cluster analysis when K = 29; (b) the numbers of each cell are corresponding to the above dendrogram figure, the colors in Global Map are corresponding to the bioregions/bioprovinces in Fig 7. Asterisk indicates the outliers, means that even the cells are distributing very far from other cells from the same cluster during the cluster analysis. Source: global basic map was downloaded from ArcWorld Supplement via ESRI and [52]), then adapted for visualization here by using open source Geographic Information System QGIS (http://qgis.osgeo.org). (TIF) [file pone.0259004.s006.tif]
