# Supplementary material for: Global biogeography of living brachiopods: Bioregionalization patterns and possible controls
Source: PLoS One. 2021 Nov 8;16(11):e0259004. doi: 10.1371/journal.pone.0259004 (PMC8575269; doi:10.1371/journal.pone.0259004)
Supplement: S1 Table — (DOCX) [file pone.0259004.s007.docx]

Supplementary Table 1: list of living brachiopod species, only geo-referenced occurrences and analysed species included.

| **No.** | **Species name on Brachnet** | **Synonyms (Synonyms names are mainly referenced from WoRMS World Register of Marine Species) (paper sources are showing in parentheses)** |
| --- | --- | --- |
|  | *Abyssorhynchia craneana* (Dall, 1895) | *Hemithyris craneana* Dall (Jackson, 1918, Dall, 1920); *Hispanirhynchia craneana* (Zezina, 1976a, Zezina, 1985); *Hispanirhynchia? Chiliensis* Foster, 1974 (Peck and Harper (2010)) |
|  | *Abyssothyris atlantica* (Cooper, 1977) |  |
|  | *Abyssothyris wyvillei*(Davidson, 1878) | *Gryphus wyvilli* Davidson (Dall, 1920); *Abyssothyris elongata* (Cooper, 1982, 1983, Harper and Peck (2016), Logan, 2007); *Abyssothyris Parva* (Cooper, 1977) (Cooper, 1977, Zezina, 2010, Peck and Harper (2010), Emig, 2017) |
|  | *Acanthobasiliola doederleini* (Davidson, 1886) | *Hemithyris doderleini* Davidson (Dall, 1920); *Rhynchonella doederleini* (Zezina, 1990);  *Tegulorhynchia doederleini* (Hatai, 1941, Cooper, 1959, 1986; Jackson and Stiasny, 1937, Zezina, 1981) |
|  | *Acrobelesia cooperi* (d'Hondt, 1976) | *Gryphus cooperi* Hondt, 1976 (Zezina, 1985, BIOCEAN database) |
|  | *Acrobrochus blochmanni* (Jackson, 1912) | *Liothyrella blochmanni* (Zezina, 1970 (1965?), 1981, 1985, 1994, Foster, 1974, Peck and Harper (2010), Harper and Peck (2016)); *Liothyrella multiporosa* Foster, 1974 (Zezina, 1985, 1994, 2010, Zezina, 2010, Foster, 1974, Peck and Harper (2010)); *Liothyrella scotti* (Foster, 1974) (Foster, 1974, Zezina, 1985, 2010); *Liothyrina blochmani* Jackson (Jackson, 1918); *Liothyris blochmanni* (Zezina, 1976a); *Liothyrella blochmanni* (Foster, 1974) |
|  | *Acrobrochus hendleri* (Cooper, 1982) |  |
|  | *Acrobrochus marotiriensis* Bitner, 2007 |  |
|  | *Acrobrochus vema* (Cooper, 1973) | *Liothyrella hendleri* Cooper, 1982 (Cooper, 1982, Peck and Harper (2010), Harper and Peck (2016)); *Liothyrella vema* Cooper, 1973 (Cooper, 1973d, 1982, Zezina, 1985, Peck and Harper (2010)) |
|  | *Aerothyris kerguelensis* (Davidson, 1878) | *Aerothyris eichleri* Allen, 1939 (Zezina, 1981); *Aneboconcha eichleri* (Zezina, 1985, 2010); *Magellania kerguelenensis* Davidson (Jackson, 1918, Dall, 1920, Zezina, 1980, Cooper, 1973b, Zezina, 1985, 2010, Peck and Harper (2010), Foster, 1974); *Waldheimia kerguelensis* Davidson, 1886 (Peck and Harper (2010)) |
|  | *Aerothyris macquariensis* (Thomson, 1918) | *Magellania macquariensis* (Foster, 1974, Cooper, 1981a, Lee, 1991, Peck and Harper (2010), Harper and Peck (2016), Laperousaz, 2017) |
|  | *Agulhasia davidsoni* King, 1871 |  |
|  | *Amphithyris cavernicola* Nauendorf, Wörheide & Lüter, 2014 |  |
|  | *Amphithyris comitodensis* Nauendorf, Wörheide & Lüter, 2014 |  |
|  | *Amphithyris hallenttensis* Foster, 1974 | *Amphithyris cavernicola* (Emig, 2017); *Amphithyris comitodentis* (Nauendorf et al., 2014, Emig, 2017); *Amphithyris parva* (Emig, 2017); *Amphithyris richardsonae* (Emig, 2017); *Amphithyris seminula* (Emig, 2017) |
|  | *Amphithyris parva* MacKinnon, Hiller, Long et Marshall, 2008 |  |
|  | *Amphithyris richardsonae* Campbell et Fleming, 1981 |  |
|  | *Amphithyris seminula* (Philippi, 1836) | *Platidia seminula* Philippl (Dall, 1920); *Terebratula seminula* Philippi, 1843 (Zezina, 1990) |
|  | *Amphithyris buckmani* Thomson, 1918 |  |
|  | *Anakinetica cumingii* (Davidson, 1852) | *Magadina cumingi* (Davidson, 1852) (Dall, 1920, Cooper, 1973d, Richardson, 1981, Zezina, 1985, 2010); *Magasella cumingi* Davidson, sp. (Davidson, 1887) |
|  | *Aneboconcha smithii* (Pfeffer, 1886) |  |
|  | *Aneboconcha obscura* Cooper, 1973 |  |
|  | *Annuloplatidia annulata* (Atkins, 1959) | *Platidia annulata* Atkins, 1959 (Peck and Harper (2010)) |
|  | *Annuloplatidia curiosa* Bitner, 2014 |  |
|  | *Annuloplatidia horni* (Gabb, 1861) | *Morrisia hornii* Gabb (Zezina, 1970) |
|  | *Annuloplatidia richeri* Bitner, 2009 |  |
|  | *Annuloplatidia indopacifica* Zezina, 1981 |  |
|  | *Arctosia arctica* (Friele, 1877) | *Gryphus arctica* Friele (Dall, 1920); *Liothyrella arctica* (Friele, 1877) (Zezina, 1997 a, b, c, d) |
|  | *Argyrotheca angulata* Zezina, 1987 |  |
|  | *Argyrotheca australis* (Blochmann, 1910) | *Cistella australis* Blochmann , 1910 (Peck and Harper (2010)) |
|  | *Argyrotheca barrettiana* (Davidson, 1866) | *Cistella barrettiana* (Davidson, 1866) (Peck and Harper (2010)) |
|  | *Argyrotheca bermudana* Dall, 1911 |  |
|  | *Argyrotheca cistellula* (Wood, 1841) | *Cistella cistellula* Searles Wood (Peck and Harper (2010)) |
|  | *Argyrotheca cooperi* Bitner et Logan, 2013 |  |
|  | *Argyrotheca crassa* Cooper, 1977 |  |
|  | *Argyrotheca furtiva* Simon, 2010 |  |
|  | *Argyrotheca grandicostata* Logan, 1983 |  |
|  | *Argyrotheca hewatti*Cooper, 1977 |  |
|  | *Argyrotheca jacksoni*Cooper, 1973 |  |
|  | *Argyrotheca johnsoni* Cooper, 1934 |  |
|  | *Argyrotheca lowei* Hertlein et Grant, 1944 |  |
|  | *Argyrotheca lutea* (Dall, 1871) | *Cistella lutea* Dall, 1870 (Peck and Harper (2010)) |
|  | *Argyrotheca mayi* (Blochmann, 1914) |  |
|  | *Argyrotheca neocaledonensis* Bitner, 2010 |  |
|  | *Argyrotheca rubrocostata* Cooper, 1977 |  |
|  | *Argyrotheca rubrotincta* (Dall, 1871) |  |
|  | *Argyrotheca schrammi* (Crosse et Fischer, 1866) |  |
|  | *Argyrotheca somaliensis* Cooper, 1973 |  |
|  | *Argyrotheca thurmanni* Cooper, 1973 |  |
|  | *Argyrotheca woodwardiana* (Davidson, 1866) | *Cistella woodwardiana* Davidson 1866 (Peck and Harper (2010)) |
|  | *Argyrotheca cuneata* (Risso, 1826) | *Cistella cuneata* Risso (Peck and Harper (2010)); *Megathiris barroisi*(Schulgin, 1884) (Zezina, 1981, 2010) |
|  | *Aulites crosnieri* Bitner, 2009 |  |
|  | *Aulites brazieri* (Crane, 1886) | *Cryptopora brazieri* (Crane, 1866) (Zezina, 1980, 1985, 2010; *Atretia brazieri* Crane, 1866 (Richardson, 1987)) |
|  | *Basiliola arnaudi* Cooper, 1981 |  |
|  | *Basiliola elongata* Cooper, 1959 |  |
|  | *Basiliola lucida* (Gould, 1862) | *Hemithyris lucida* Gould (Dall, 1920); *Neohyemithyris lucida* (Gould) (Hatai, 1936a); *Rhynchonella lucida* Gould, 1871 (Peck and Harper (2010)) |
|  | *Basiliola pompholyx* Dall, 1920 |  |
|  | *Basiliola beecheri* (Dall, 1895) | *Hemithyris beecheri* Dall, 1895 (Dall, 1895) |
|  | *Basiliolella colurnus* (Hedley, 1905) | *Hemithyris colurnus* Hedley (Dall, 1920, Peck and Harper (2010)); *Eohermithyris colurnis* (Harper and Peck (2016), Peck and Harper (2010), Richardson, 1986, Zezina, 1981, Zezina, 1985, Zezina, 2009, 2000, Museums Victoria Marine Invertebrates Collection) |
|  | *Basiliolella grayi* (Woodward, 1855) | *Basiliolella ferox* d'Hondt, 1987 (Peck and Harper (2010)); *Eohemithyris grayi* (Woodward, 1885) (Laurin 1997, Bitner, 2009); *Rhynchonella grayi*, Woodward, 1855 (Peck and Harper (2010)) |
|  | *Bathynanus inversus* Zezina, 1981 |  |
|  | *Bathynanus rhizopodus* Zezina, 1981 |  |
|  | *Bathynanus tenuicostatus* Foster, 1974 |  |
|  | *Bouchardia rosea* (Mawe, 1823) |  |
|  | *Calloria variegata* Cooper et Doherty, 1993 |  |
|  | *Calloria inconspicua* (Sowerby, 1846) | *Terebratella inconspicua* (Richardson, 1981, Dall, 1920, Zezina, 2010, Campbell and Fleming, 1981); *Waltonia inconspicua* (Sowerby, 1846) (Bowen, 1968, Lee, 1991, Cooper, 1982, Zezina, 1985) |
|  | *Campages asthenia* (Dall, 1920) |  |
|  | *Campages dubius* Hatai, 1940 | *Japanithyris dubias* Hatai, 1940 (Zezina, 1981, 1985, 2010); *Campages japonica* Hatai, 1940 (Zezina, 1981, 1985, 2010) |
|  | *Campages furcifera* (Hedley, 1905) |  |
|  | *Campages japonica* (Hatai, 1940) |  |
|  | *Campages nipponensis* Yabe et Hatai, 1935 |  |
|  | *Campages ovalis* Bitner, 2008 |  |
|  | *Campages pacifica* (Hatai, 1940) |  |
|  | *Campages mariae*(Adams, 1860) | *Campages basilanica* (Dall, 1920, Hatai, 1936a, Logan, 2007, Zezina, 1985, 2010, Peck and Harper (2010)); *Japanithyris mariae* (Adams, 1860) (Hatai, 1936a, Zezina, 1985, 2010); *Terebratalia mariae* A. Adams (Dall, 1920) |
|  | *Cancellothyris hedleyi* (Finlay, 1927) | *Terebratulina cancellata* (Dall, 1920) |
|  | *Chlidonophora chuni* Blochmann, 1903 |  |
|  | *Chlidonophora incerta* (Davidson, 1878) | *Chlidonophora* (described as *Megerlia) incerta* (Davidson, 1878) (Peck and Harper (2010)); *Chlidonophoridae incerta* (Davidson, 1878) (Zezina, 2010) |
|  | *Cnismatocentrum parvum* Zezina, 1970 |  |
|  | *Cnismatocentrum sakhalinensis* (Dall, 1908) | *Gryphus sakhalinensis* Dall (Dall, 1920) |
|  | *Compsothyris ballenyi*Foster, 1974 |  |
|  | *Compsothyris racovitzae* (Joubin, 1901) | *Rhynchonella recovitzae* Joubin, 1901 (Joubin, 1901) (Peck and Harper (2010)); *Hispanirhynchia antartica* Hatai, 1959 (Zezina, 1970 (1965?)); *Compsothyris antarctica* (Hatai, 1959) (Zezina, 1990) |
|  | *Coptothyris grayii* (Davidson, 1852) | *Coptothyris grayi* (Davidson) (Hatai, 1936a, Logan, 2007, Álvarez et al., 2017); *Coptothyris adamsi* (Davidson, 1871) (Zezina, 1997 (a, b, c, d), 1981, 1985, 2010); *Magasella gouldi* Dall (Peck and Harper (2010)); *Pereudesia grayi* Davidson (Dall, 1920) |
|  | *Cryptopora boettgeri* Helmcke, 1940 |  |
|  | *Cryptopora curiosa* Cooper, 1973 |  |
|  | *Cryptopora hesperis* Cooper, 1982 |  |
|  | *Cryptopora maldiviensis* Muir-Wood, 1959 |  |
|  | *Cryptopora norfolkensis*Bitner, 2009 |  |
|  | *Cryptopora rectimarginata* Cooper, 1959 |  |
|  | *Cryptopora gnomon* Jeffreys, 1869 | *Atretia gnomon* Jeffreys (Dall, 1920) |
|  | *Dallina elongata* Hatai, 1940 |  |
|  | *Dallina eltanini* Foster, 1974 |  |
|  | *Dallina floridana* (Pourtalès, 1867) |  |
|  | *Dallina obessa* Yabe et Hatai, 1934 |  |
|  | *Dallina parva*Cooper, 1981 |  |
|  | *Dallina raphaelis* (Dall, 1870) |  |
|  | *Dallina triangularis*Yabe et Hatai, 1934 |  |
|  | *Dallina septigera* (Lovén, 1845) | *Magellania septigera* (BIOCEAN database) |
|  | *Dallinella occidentalis* (Dall, 1871) | *Terebratalia occidentalis* Dall (Dall, 1920, Peck and Harper (2010)) |
|  | *Dallinella obsoleta* (Dall, 1891) | *Terebratalia obsoleta* Dall (Du Bois, 1916, Dall, 1920) |
|  | *Dallithyris dubia* Cooper, 1981 |  |
|  | *Dallithyris elongata* Cooper 1977 |  |
|  | *Dallithyris murrayi* Muir-Wood, 1959 |  |
|  | *Dallithyris pacifica* Bitner, 2006 |  |
|  | *Dallithyris tahitiensis* Bitner, 2014 |  |
|  | *Dallithyris fulva* (Blochmann, 1906) | *Epacrosina fulva* (Blochmann) (Cooper, 1983, Laperousaz, 2017, Western Australian Museum); *Gryphus fulva* Blochmann (Dall, 1920); *Liothyrella fulva* (Blochmann, 1906) (Zezina, 1981, 1985); *Dallithyris (Epacrosina) fulva* (Blochmann, 1906) (Zezina, 2010) |
|  | *Diestothyris tisimana* (Nomura et Hatai, 1936) | *Terebratalia (Tisimania) tisimana* (Nomura & Hatai, 1936) (Zezina, 1997 a, b, c, d, 1981, 2010) |
|  | *Diestothyris frontalis* (Middendorff, 1849) | *Terebratalia frontalis* Middendorff (Dall, 1920); *Terebratella frontalis* Middendorff (Davidson, 1887, Peck and Harper (2010)); *Terebratalia tisimana* Nomura & Hatai, 1936 |
|  | *Discina striata* (Schumacher, 1817) |  |
|  | *Discinisca laevis* (Sowerby, 1822) |  |
|  | *Discinisca rikuzenensis*(Hatai, 1940) |  |
|  | *Discinisca tenuis* (Sowerby, 1847) |  |
|  | *Discinisca lamellosa* (Broderip, 1834) | *Discina lamellosa* Broderip (Dall, 1920) |
|  | *Discradisca cumingii* (Broderip, 1833) | *Discinisca cumingi* (Broderip, 1833) (Dall, 1920, Zezina, 1985, 1990) |
|  | *Discradisca indica* (Dall, 1920) | *Discinisca indica* Dall (Dall, 1920, Cooper, 1973b, Zezina, 1985) |
|  | *Discradisca sparselineata* (Dall, 1920) |  |
|  | *Discradisca stella* (Gould, 1862) | *Discinisca stella* (Gould, 1860) (Dall, 1920, Hatai, 1936a, Zezina, 1981, 1985) |
|  | *Discradisca strigata* (Broderip, 1834) | *Discinisca strigata* (Broderip, 1834) (Dall, 1920, Zezina, 1981, 1985) |
|  | *Discradisca antillarum* (d'Orbigny, 1846) | *Discinisca antillarum* (Orbigny, 1846) (Dall, 1920, Zezina, 1990) |
|  | *Dolichozygus*stearnsii (Dall et Pilsbry, 1891) | *Dallithyris stearnsi* (Dall et Pilsbry, 1892) (Zezina, 1985, BIOCEAN database); *Gryphus stearnsi* Dall & Pilsbry(Hatai, 1936a); *Liothyrella sternsi* (Dall and Pilsbry, 1891) (Zezina, 1981); *Dallithyris (Dallichozygus) sternsi* (Dall et Pilsbry, 1891) (Zezina, 2010) |
|  | *Dyscolia johannisdavisi* (Alcock, 1894) |  |
|  | *Dyscolia subquadrata* (Jeffreys, 1878) | *Gryphus subquadratus* Jeffreus (Dall, 1920) |
|  | *Dyscolia wyvillei* (Davidson, 1878) | *Terebratulina* (now=*Dyscolia) wyvilli* Davidson (1878) (Peck and Harper (2010)) |
|  | *Dyscritosia secreta* Cooper, 1982 |  |
|  | *Dysedrosia borneoensis* (Dall, 1920) | *Gryphus borneoensis* (Dall, 1920, Zezina, 1985); *Liothyrella borneoensis* (Dall, 1920) (Zezina, 1981) |
|  | *Ebiscothyris bellonensis* Bitner et Cohen, 2015 |  |
|  | *Ecnomiosa inexpectata* Cooper, 1981 |  |
|  | *Ecnomiosa gerda* Cooper, 1977 |  |
|  | *Erymnia angustata* Cooper, 1977 |  |
|  | *Erymnia muralifera* Cooper, 1977 |  |
|  | *Eucalathis cubensis* Cooper, 1977 |  |
|  | *Eucalathis daphneae* Bitner et Logan, 2016 |  |
|  | *Eucalathis ergastica* Fischer et Œhlert, 1890 |  |
|  | *Eucalathis fasciculata* Cooper, 1973 |  |
|  | *Eucalathis floridensis* Cooper, 1977 |  |
|  | *Eucalathis inflata* Cooper, 1973 |  |
|  | *Eucalathis macrorhynchus* Forster, 1974 |  |
|  | *Eucalathis magna* Cooper, 1981 |  |
|  | *Eucalathis malgachensis* Bitner et Logan, 2016 |  |
|  | *Eucalathis rugosa* Cooper, 1973 |  |
|  | *Eucalathis trigona* (Jeffreys, 1878) |  |
|  | *Eucalathis tuberata* (Jeffreys, 1878) |  |
|  | *Eucalathis murrayi*(Davidson, 1878) | *Eucalathis costella* (Cooper, 1981a); *Eucalathis costellata* (Cooper, 1981) (Zezina, 1985, 2010, Logan, 2007, Peck and Harper (2010), BIOCEAN database, IndOBIS); *Eucalathis rotundata* (Cooper, 1981a) (Cooper, 1981a, Zezina, 1985, 2010, Logan, 2007, Peck and Harper (2010), BIOCEAN database, IndOBIS) |
|  | *Fallax antarcticus* Foster, 1974 |  |
|  | *Fallax neocaledonensis* Laurin, 1997 | *Laurinia neocaledonensis* (Logan, 2007, Zezina, 2005, 2010) |
|  | *Fallax dalliniformis* Atkins, 1960 |  |
|  | *Fosteria spinosa* (Foster, 1974) | *Magellania spinosa* Foster, 1974 (Foster, 1974, Zezina, 1980) |
|  | *Frenulina cruenta*Cooper, 1973 |  |
|  | *Frenulina mauiensis* Dall, 1920 |  |
|  | *Frenulina sanguinolenta*(Gmelin,1790) |  |
|  | *Frieleia pellucida* (Yabe et Hatai, 1934) | *Hemithyris pellucida* Yabe & Hatai (Hatai, 1936a) |
|  | *Frieleia halli*Dall, 1895 |  |
|  | *Glaciarcula spitzbergensis* (Davidson, 1852) | *Glaciarcula* (described as *Terebratella) spitzbergensis* Davidson, 1852 (Peck and Harper (2010)); *Terebratalia spitzbergensis* Davidson (Dall, 1920, Davidson, 1887); *Glaciarcula frieli* (Davidson, 1878) (Zezina, 1981, 1985, 1994, 2010) |
|  | *Glottidia audebarti* (Broderip, 1833) |  |
|  | *Glottidia palmeri* Dall, 1871 |  |
|  | *Glottidia pyramidata* (Stimpson, 1860) |  |
|  | *Glottidia albida* (Hinds, 1844) |  |
|  | *Goniobrochus ewingi*(Cooper, 1973) | *Dyscolia ewingi* Cooper, 1973 (Cooper, 1973d, 1983, Zezina, 1985, Peck and Harper (2010)); *Dyscolia radiata* Cooper, 1981 |
|  | *Grammetaria africana*Hiller, 1986 |  |
|  | *Grammetaria minima* Zezina, 1994 |  |
|  | *Grammetaria bartschi*(Dall, 1920) | *Hemithyris bartschi* Dall, 1920 (Dall, 1920, Logan, 2007, Peck and Harper (2010)) |
|  | *Gryphus capensis* Jackson, 1952 | *Liothyrella capensis* Jackson, 1952 (Zezina, 1981) |
|  | *Gryphus clarkeana* (Dall, 1895) | *Liothyrella clarkeana* (Dall, 1895) (Zezina, 1981, 1985, 1994); *Liothyris clarkeana* (Zezina, 1976a) |
|  | *Gryphus tokionis*Dall, 1920 | *Liothyrella tokionis* (Dall, 1920) (Zezina, 1981) |
|  | *Gryphus vitreus* (Born, 1778) | *Liothyrella vitrea* (Born, 1778) (Zezina, 1981); *Liothyris vitrea* (Zezina, 1976a); *Gryphus vitreus* (Born) (Emig, 1987, Cooper, 1981b, Logan et al., 2002) |
|  | *Gwynia macrodentata*Lüter, 2008 |  |
|  | *Gwynia capsula* (Jeffreys,1859) |  |
|  | *Gyrothyris williamsi* Bitner, Cohen, Long, Richer de Forges et Saito, 2008 |  |
|  | *Gyrothyris mawsoni* Thomson, 1918 | *Gyrothyris mawsoni antipodesensis* (Foster, 1974); *Gyrothyris mawsoni aucklandensis* (Foster, 1974); *Gyrothyris mawsoni mawsoni* (Foster, 1974) |
|  | *Hemithiris woodwardi* (Adams, 1863) |  |
|  | *Hemithiris psittacea* (Gmelin, 1790) | *Rhynchonella psittacea* Gmelin (Davidson, 1887) |
|  | *Hillerella bisepta* Simon, Logan et Mottequin, 2016 |  |
|  | *Hispanirhynchia cornea* (Fischer, 1887) | *Hemithyris cornea* Fischer (Dall, 1920); *Rhynchonella cornea* Fischer (Jackson, 1918, Peck and Harper (2010)) |
|  | *Jaffaia jaffaensis* (Blochmann, 1910) | *Campages jaffaensis* Blochmann (Dall, 1920) |
|  | *Joania arguta* Grant, 1983 | *Argyrotheca arguta* Grant, 1983 (fide Bitner, 2008) (Zezina, 2010, Logan, 2007, Simon, 2010) |
|  | *Joania cordata* (Risso, 1826) | *Argyrotheca cordata* (Risso 1826) (Dall, 1920, Zezina, 1985, Martinez et al. 2005, Peck and Harper (2010), Asgaard & Bromley, 1991, Logan et al., 1997, 2002, Zezina, 1981, Taddei Ruggiero, 2001, Logan, 2007, Jackson, Goreau, Hartman, 1971; Alvarez et al. 2008) |
|  | *Jolonica alcocki* (Joubin, 1906) | *Compsoria alcocki* (Joubin, 1906) (Zezina, 1985); *Frenulina alcocki* (Joubin, 1907) (Dall, 1920, Cooper, 1973b); *Kingena alcocki Joubin* (Cooper, 1973b); *Frenulina* (=*Compsoria*) *alcocki* (Joubin, 1907) (Cooper, 1973b) |
|  | *Jolonica nipponica* Yabe et Hatai, 1934 |  |
|  | *Jolonica suffusa* (Cooper, 1973) | *Compsoria* (now = *Jolonica) suffusa* Cooper, 1973 (Peck and Harper (2010)); *Compsoria suffusa* (Cooper) (Cooper, 1973b, Zezina, 1985, Hiller, 1986, Brand et al., 2003; MacKinnon & Hiller 2010) |
|  | *Jolonica hedleyi* Dall, 1920 | *Campages (jolonica) hedleyi* (Dall, 1920) |
|  | *Kakanuiella chathamensis* Lüter, 2005 |  |
|  | *Kanakythyris pachyrhynchos*Laurin, 1997 |  |
|  | *Kraussina cognata* (Sowerby, 1847) |  |
|  | *Kraussina crassicostata* Jackson, 1952 |  |
|  | *Kraussina gardineri*Dall, 1910 |  |
|  | *Kraussina mercatori* Helmcke, 1939 |  |
|  | *Kraussina rubra* (Pallas, 1766) |  |
|  | *Lacazella caribbeanensis* Cooper, 1977 |  |
|  | *Lacazella mauritiana* Dall, 1920 |  |
|  | *Lacazella mediterranea* (Risso, 1826) | *Thecidea mediterranea* Risso, 1826 |
|  | *Laqueus blanfordi*(Dunker, 1882) | *Terebratella blanfordi* Dunker (Davidson, 1887) |
|  | *Laqueus concentricus* Yabe et Hatai, 1936 |  |
|  | *Laqueus japonicus* Yabe & Hatai, 1934 |  |
|  | *Laqueus jeffreysi* Dall, 1877 |  |
|  | *Laqueus morsei* Dall, 1908 |  |
|  | *Laqueus orbicularis* Yabe et Hatai, 1934 |  |
|  | *Laqueus pacificus*Hatai, 1936 |  |
|  | *Laqueus pallidus* Hatai, 1939 |  |
|  | *Laqueus proprius* Yabe et Hatai, 1934 |  |
|  | *Laqueus quadratus* Yabe et Hatai, 1934 |  |
|  | *Laqueus rubellus* (Sowerby, 1846) |  |
|  | *Laqueus suffusus* (Dall, 1870) |  |
|  | *Laqueus vancouveriensis* Davidson, 1887 | *Laqueus californianus* var*. vancouveriensis* Davidson, 1887 (Tunnicliffe & Wilson, 1988, MacKinnon & Long, 2000, Sloan et al., 2004) |
|  | *Laqueus erythraeus* Dall, 1920 | *Laqueus californicus* (MacKinnon & Long, 2001) |
|  | *Lenticellaria marerubris*Simon, Logan et Mottequin, 2016 |  |
|  | *Lenticellaria gregoryi* Simon, Logan et Mottequin, 2016 |  |
|  | *Leptothyrella fijiensis* Bitner, 2008 |  |
|  | *Leptothyrella incerta* (Davidson, 1880) | *Magasella incerta* (Davidson, 1880) *Phaneropora incerta* (Zezina, 1985, 2010, 2014, Martinez et al. 2005, BIOCEAN database, Gaspard, 2003, Logan, 1998); *Phaneropora incertae* (Davidson, 1878) (Zezina, 1994, Peck and Harper (2010)); *Platidia incerta* (Zezina, 1970, Zezina, 1976, ) |
|  | *Leptothyrella ignota* (Muir-Wood, 1959) |  |
|  | *Lingula adamsi* Dall, 1873 | *Lingula shantungensis* Hatai, 1931 (Emig & Hammond, 1986, Hatai, 1942, Ishida, 2016) |
|  | *Lingula parva* Smith, 1871 |  |
|  | *Lingula reevei*Davidson, 1880 |  |
|  | *Lingula rostrum* (Shaw, 1798) | *Lingula japspidea* Adams, 1863 (Dall, 1920, Hatai, 1941, Hatai, 1942, Zezina, 1985) |
|  | *Lingula translucida* Dall, 1920 |  |
|  | *Lingula tumidula* Reeve, 1841 |  |
|  | *Lingula unguis* (Linnaeus, 1758) | *Lingula unguis* Dall (Zezina, 1970) |
|  | *Lingula anatina* Lamarck, 1801 | *Lingula* aff*. L. exusta* Reeve, 1859 (Cooper, 1973b)*; Lingula bancrofti* Johnsion and Hirschfeld (Dall, 1920, Hatai, 1937, Craig, 1952); *Lingula exusta* Reeve (Dall, 1920, Hatai, 1937); *Lingula hians Swainson* (Cooper, 1973b); *Lingula murphiana* Reeve, 1859 (Dall, 1920, Hatai, 1936a, Hatai, 1937, Cooper, 1973b, Zezina, 1970); *Lingula lepidula* A. Adams (Dall, 1920, Hatai, 1941, Hatai, 1942, Zezina, 2009); *Lingula smaragdina* Adams (Hatai, 1941); *Lingula nipponica* Hayasaka, 1931 (Hatai, 1941, 1942) |
|  | *Liothyrella delsolari* Cooper, 1982 | *Liothyrella desolari*? Cooper, 1982 (Foster, 1974, Cooper, 1982, 1983, Logan, 2007, Peck and Harper (2010)) |
|  | *Liothyrella moseleyi* (Davidson, 1878) | *Gryphus moseleyi* Davidson (Dall, 1920) |
|  | *Liothyrella neozelanica* Thomson, 1918 |  |
|  | *Liothyrella winteri* (Blochmann, 1906) |  |
|  | *Liothyrella uva* (Broderip, 1883) | *Gryphus antarctica* Blochmann (Dall, 1920); *Liothyrella antarctica* (Blochmann, 1906) (Jackson, 1918, Cooper, 1983, Zezina, 1970 (1965?), Zezina, 1980, 1985, 1994, 2010, Logan, 2007, Southwestern Pacific OBIS, 2014); *Liothyrella expansa* (Cooper, 1982) (Cooper, 1983, Zezina, 2010, Peck and Harper (2010)); *Liothyrella fosteri* (Cooper, 1982) (Cooper, 1982, 1983, Zezina, 2010, Peck and Harper (2010)); *Liothyrella georgian*a Foster (Cooper, 1982); *Liothyrella notocadensis* (Cooper, 1983, Zezina, 2010, Ayala et al., 1975, Harper and Peck (2016), Peck and Harper (2010), Brand et al., 2003); *Liothyrella oblonga* (Cooper, 1973) (Cooper, 1973d, 1983， Zezina, 1985, 2010, Logan, 2007, Peck and Harper (2010)); *Liothyrella ovata* (Thomson, 1918) (Zezina, 1970 (1965?), 1985, 2010); *Liothyrella uva antarctica* (Blochmann, 1906) (Foster, 1974); *Liothyrella uva cancerderma* (Foster, 1974); *Liothyrella uva clarkeana* (Dall, 1895) (Zezina, 2010); *Liothyrella uva georgiana* Foster, 1974(Cooper, 1983, Foster, 1974, Zezina, 2010); *Liothyrella uva notorcardensis* Jackson, 1912 (Peck et al., 1987, Foster, 1974); *Liothyrella uva uva* Broderip, 1833 (Foster, 1974); *Liothyrella uva* var. *notorcardensis* Jackson (Jackson, 1918); *Liothyris antarctica* (Zezina, 1976a); *Stenosarina expansa* (Cooper) (Cooper, 1983) |
|  | *Macandrevia africana* Cooper, 1975 |  |
|  | *Macandrevia americana* Dall, 1895 | *Macandrevia americana diegensis* Dall (Cooper, 1982); *Macandrevia americana vanhoeffeni* Blochmann, 1906 (Foster, 1974); *Macandrevia craniella* (Dall, 1920, Zezina, 1976a, 1985, 1994, 2010, Cooper, 1982, Logan, 2007); *Macandrevia lata* Thomson, 1918 (Zezina, 1970 (1965?)); *Macandrevia vanhoeffeni* Blochman, 1906 (Jackson, 1918, Zezina, 1980, 1985, 1990, 2010, Cooper, 1982, Logan, 2007, Foster, 1974, Museums Victoria Marine Invertebrates Collection); *Macandrevia waldae* (Hondt, 1976) (Zezina, 1985, 1994, 2010) |
|  | *Macandrevia bayeri*Cooper, 1975 |  |
|  | *Macandrevia diamantina* Dall, 1895 | *Macandrevia (Notorygmia) diamantina* Dall, 1895 (Zezina, 1980, 1985); *Macandrevia abyssa* Cooper, 1972 (Logan, 2007); *Notorygmia diamantina* (Zezina, 1976a, 1981, Harper and Peck (2016)) |
|  | *Macandrevia emigi* Bitner et Logan, 2016 |  |
|  | *Macandrevia tenera* (Jeffreys, 1876) |  |
|  | *Macandrevia cranium* (Müller, 1776) | *Macandrevia novangliae* Dall 1920 (Cooper, 1977, 1981b, Martinez et al. 2005, Logan, 2007, Peck and Harper (2010)) |
|  | *Magadinella mineuri* Richardson, 1987 |  |
|  | *Magasella haurakiensis* (Allan, 1931) | *Terebratella haurakiensis* Allan 1931 (Lee, 1991, NIWA Invertebrate Collection, 2019, Blom and Moriarty, 2018, Logan, 2007, Zezina, 2010, Robinson et al., 2016) |
|  | *Magasella sanguinea* (Leach, 1814) | *Terebratella (Magasella) sanguinea* (Leach. 1814) (Zezina, 1980, 1985, 2010); *Terebratella cruenta* Dillwyn (Davidson, 1887); *Terebratella haurakiensis* (Lee, 1991, Logan, 2007, Zezina, 2010, Peck and Harper (2010)); *Terebratella rubicunda* (Sowerby, 1846) (Jackson, 1918, Davidson, 1887); *Terebratella rubiginosa* Dall, 1871 (Dall, 1920, Hiller, 1986, Cooper, 1973b, Zezina, 1985, Logan, 2007); *Terebratella sanguinea* (Dall, 1920, Jackson, 1918, Lee, 1991, Richardson, 1981, Parkinson et al., 2005, Peck and Harper (2010), Harper and Peck (2016), Parkinson et al., 2005, Cohen et al., 2011, Álvarez et al., 2017, Harper, 2011, Logan, 2007, Seidel and Lüter, 2014, Foster, 1974, Bitner et al., 2008b, NatureWatch NZ, 2016, NIWA Invertebrate Collection, 2019, Queensland Museum, Southwestern Pacific OBIS, 2014, SWPRON, 2018, Blom and Moriarty, 2018, SCAR - AntOBIS (2019), Campbell and Fleming, 1981, Lee, 1991, Seidel and Lüter, 2014, Robinson et al., 2016) |
|  | *Magellania fragilis* Smith, 1907 | *Stethothyris antarctica* (Thomson, 1918) (Zezina, 1985, 2010); *Aerothyris fragilis* (Smith, 1907) (Zezina, 1975, 1980, 1985, 2010); *Victorthyris antarctica* (Thomson) (Zezina, 1975) |
|  | *Magellania joubini* Blochmann, 1906 | *Aerothyris joubini* (Blochmann, 1906) (Zezina, 1970, 2010) |
|  | *Magellania venosa* (Solander, 1786) | *Magellania (Neothyris) venosa* Solander (Dall, 1920) |
|  | *Magellania flavescens* (Lamarck, 1819) | *Terebratella (T.) mayi* Blochmann, 1914 (Zezina, 2010); *Terebratella mayi* Blochmann, 1914 (Zezina, 1981, 1985, Logan, 2007); *Magellania australis* (Quoy and Gaimard, 1835) (Zezina, 1981, 1985, 2010); *Magellania iredalei* Allan, 1939 (Zezina, 1981, 1985, 2010) |
|  | *Manithyris rossi* Foster, 1974 |  |
|  | *Megathiris capensis* Jackson, 1952 |  |
|  | *Megathiris detruncata* (Gmelin, 1791) | *Argiope decollata* (Peck and Harper (2010)) |
|  | *Megerella hilleri* Bitner et Logan, 2016 |  |
|  | *Megerlia acrura* Hiller, 1986 |  |
|  | *Megerlia truncata* (Linné, 1767) | *Pantellaria* (now Megerlia) *monstruosa* (Sacchi) (Peck and Harper (2010)); *Muhlfeldtia truncata* (Linne, 1767) (Zezina, 1970 (1965?)); *Megerlia gigantea* (Harper and Peck (2016), BIOCEAN database); *Megerlia echinata* (Fischer & Oehlert) (Cooper, 1973b, 1981b, 1977, Hiller, 1986, Laurin, 1997, Gaspard, 2003, Zezina, 2010, Logan, 2007, Peck and Harper (2010), BIOCEAN database); *Pantellaria echinata* (Fischer and Oehlert, 1890) (Dall, 1920, Cooper, 1973b, Zezina, 1981); *Megerlia gigantea* (Deshayes) (Cooper, 1981a, Logan, 2007, Zezina, 2010); *Pantellaria gigantea* (Deshayes, 1863) (Zezina, 1985, 1990, Cooper, 1973b); *Megerlia gigantea* (Deshayes, 1863) (Peck and Harper (2010)); *Megerlia truncata paucistriata* Jackson (Cooper, 1973b); *Megerlina echinata* (Fischer & Oehlert) (Cooper, 1973b); *Megertia monstruosa* (Scacchi 1833) (Martinez et al. 2005); *Pantellaria monstruosa* (Scacchi, 1833) (Dall, 1920, Jackson et al., 1971, Cooper, 1981b, 1982, Zezina, 1981, 1985, 1994); *Megerlia granosa* Seguenza, 1865 (Zezina, 1985, 2010); *Muhlfeldtia granosa* (Seguenza, 1855) (Zezina, 1981); *Muhlfeldtia disculus* Pallas (Dall, 1920) |
|  | *Megerlina atkinsoni* (Woods, 1878) | *Kraussina atkinsoni Tenison* Woods (Twelvetrees and Petterd, 1900, Peck and Harper (2010)) |
|  | *Megerlina capensis* (Adams et Reeve, 1850) |  |
|  | *Megerlina davidsoni* (Vélain, 1877) |  |
|  | *Megerlina natalensis* (Krauss, 1843) |  |
|  | *Megerlina pisum* (Lamarck, 1819) | *Kraussina pisum* *Valciennes* (Twelvetrees and Petterd, 1900, Peck and Harper (2010)) |
|  | *Megerlina striata* Jackson, 1952 |  |
|  | *Megerlina lamarckiana* (Davidson, 1852) | *Kraussina lamarckiana* Davidson (Twelvetrees and Petterd, 1900, Peck and Harper (2010)) |
|  | *Melvicalathis macroctena* (Zezina, 1981) | *Eucalathis macroctena* Zezina, 1981 (Zezina, 1985, 1994, 2010, Foster, 1974, Logan, 2007, Peck and Harper (2010)) |
|  | *Minutella bruntoni*Hoffmann et Lüter, 2010 |  |
|  | *Minutella minuta* (Cooper, 1981) | *Thecidellina minuta* (Cooper, 1981a, Zezina, 1985, 2010, Logan, 2007, Lee and Robinson, 2003, Bitner, 2009, 2010, Hoffmann & Lüter 2010; Peck and Harper (2010), BIOCEAN database) |
|  | *Minutella tristani*Hoffmann et Lüter, 2010 |  |
|  | *Murravia exarata* (Verco, 1910) *in* Blochmann (1910) |  |
|  | *Nanacalathis atlantica* Zezina 1991 |  |
|  | *Nanacalathis minuta* Zezina, 1981 |  |
|  | *Neoaemula vector* MacKinnon, Hiller, Long et Marshall, 2008 |  |
|  | *Neoancistrocrania norfolki* Laurin, 1992 |  |
|  | *Neorhynchia strebeli* (Dall, 1908) | *Neorhynchia profunda* (Cooper) (Brand et al., 2003, Logan, 2007); *Neorhynchia strebeli* (NIWA Invertebrate Collection, 2019); *Neorhynchia strebeli rectimarginata* Foster, 1974 (Foster, 1974, Peck and Harper (2010)) |
|  | *Neothyris ovalis* (Hutton, 1886) |  |
|  | *Neothyris lenticularis* (Deshayes, 1839) | *Magellania (Neothyris) lenticularis* Deshayes (Dall, 1920); *Neothyris compressa* Neall, 1972 (Foster, 1974, Richardson, 1997b, 1981, Logan, 2007, Zezina, 1985, 2010, NIWA Invertebrate Collection, 2019); *Neothyris dawsoni* (Richardson, 1997b, Harper and Peck (2016), Peck and Harper (2010), Zezina, 1985, 2010, Logan, 2007, NIWA Invertebrate Collection, 2019); *Neothyris parva* (Cooper, 1982, Logan, 2007, Bitner et al., 2008b, Cohen et al., 2011, Peck and Harper (2010), NIWA Invertebrate Collection, 2019) |
|  | *Nipponithyris afra*Cooper, 1973 |  |
|  | *Nipponithyris lauensis* Bitner, 2008 |  |
|  | *Nipponithyris nipponensis* Yabe et Hatai, 1934 | *Japanithyris nipponensis* Yabe & Hatai, 1935 (Hatai, 1936a, Zezina, 1985, 2010) |
|  | *Notosaria reinga* Lee et Wilson, 1979 |  |
|  | *Notosaria nigricans* (Sowerby, 1846) |  |
|  | *Notozyga gracilis* Hiller, 1986 |  |
|  | *Notozyga lowenstami* Cooper, 1977 | *Eucalathis lowenstami* Cooper, 1977 (Zezina, 1985) |
|  | *Novocrania altivertex* Zezina, 1990 | *Crania altivertes* Zezina, 1990; *Novocrania altivertex*Zezina, 1990 (Zezina, 2010) |
|  | *Novocrania hawaiiensis* (Dall, 1920) | *Crania hawaiiensis* Dall, 1920 (Dall, 1920, Zezina, 1985, 1990, 2010) |
|  | *Novocrania huttoni* (Thomson, 1916) | *Neocrania huttoni* (Lee, 1991); *Crania huttoni* (Bowen, 1968) |
|  | *Novocrania indonesiensis* (Zezina, 1981) |  |
|  | *Novocrania lecointei* (Joubin, 1901) | *Crania joubini* Thomson, 1918 (Zezina, 1975); *Crania lecointei* Joubin, 1901 (Zezina, 1975, 1980, 1985); *Crania valdiviae* Helmcke, 1940 (Cooper, 1973b, Zezina, 1985, 2010); *Crania patagonica* Dall (Dall, 1920, Zezina, 1970, Cooper, 1973d); *Crania lecointei* (Foster, 1974); *Crania patagonia* (Cooper 1973b) |
|  | *Novocrania philippinensis* (Dall, 1920) | *Crania californica* Berry 1921 (Tunnicliffe & Wilson, 1988, Zezina, 1985, 1990, 2010); *Novocrania californica* (Robinson, 2017) |
|  | *Novocrania roseoradiata* (Jackson, 1952) | *Crania roseradiata* Jackson, 1952 (Zezina, 1975, 1985, 2010); *Neocrania roseoradiata* (Jackson) *Novocrania?* (Hiller, 1986) |
|  | *Novocrania turbinata*(Poli, 1795) | *Crania japonica* (Adams) (Logan and Long, 2001); *Crania kermes* Costa and Humphrey, 1770 (Dall, 1920, Zezina, 1990, 2010); *Crania anomala turbinata* (Poli) (Cooper, 1981b); *Crania suessi* Reeve, 1862 (Zezina, 1985, 1990, 2010); *Craniscus japonicus* (Adams, 1863) (Dall, 1920, Hatai, 1936a, Logan and Long, 2001, Zezina, 1990, 2010, Robinson, 2017); *Novocrania reevei* Lee and Brunton, 1986 (Bitner, 2010, Robinson, 2017); *Novocrania japonica* (Adams, 1863) (Robinson and Lee, 2011) |
|  | *Novocrania anomala* (Müller, 1776) | *Crania anomala* (Müller, 1776) (Zezina, 1997 (a, b, c, d), 1985, 1990, Cooper, 1981b, Flanders Marine Institute, 2004); *Crania lamellosa* Seguenza, 1865 (Dall, 1920, Zezina, 1985, 1990, 2010); *Crania pourtalesii* Dall, 1871 (Dall, 1920, Zezina, 1990, 2010, Jackson et al., 1971, Cooper, 1977, Santagata et al., 2009); *Crania rostrata* Hoeninghaus, 1828 (Dall, 1920, Zezina, 1985, 1990, 2010); *Cranla anomila* Muller (Dall, 1920); *Neocrania anomala* (Müller, 1776) (Taddei Ruggiero, 2001, Logan and Long, 2001); *Novocrania pourtalesii* (Robinson et al., 2017) |
|  | *Oceanithyris juveniformis* Bitner et Zezina, 2013 *in* Bitner *et al*. (2013) |  |
|  | *Ospreyella maldiviana* Logan, 2005 |  |
|  | *Ospreyella mayottensis*Simon, Hiller, Logan & Mottequin, 2019 |  |
|  | *Ospreyella mutiara*Simon et Hoffmann, 2013 |  |
|  | *Ospreyella palauensis* Logan, 2008 |  |
|  | *Ospreyella depressa* Lüter et Wörheide, 2003 |  |
|  | *Pajaudina atlantica* Logan, 1988 |  |
|  | *Parakinetica stewarti* Richardson, 1987 |  |
|  | *Parasphenarina ezogremena* (Zezina, 1981) | *Sphenarina exogremina* Zezina, 1981 (Zezina, 1985, 1990; Peck and Harper (2010)) |
|  | *Parasphenarina cavernicola*Motchurova-Dekova, Saito et Endo, 2002 |  |
|  | *Pelagodiscus atlanticus* (King, 1868) |  |
|  | *Pemphixina pyxidata* (Davidson, 1880) | *Hemithyris pyxidata* (Davidson) (Jackson, 1918); *Notosaria pyxidata* (Davidson) (Cooper, 1973b); *Rhynchonella nigricans* var. *pyxidata* (Jackson, 1918) |
|  | *Phaneropora galatheae* Zezina, 1981 |  |
|  | *Pictothyris elegans* Yabe et Hatai, 1936 |  |
|  | *Pictothyris laquaeformis* Yabe et Hatai, 1936 |  |
|  | *Pictothyris picta* (Dallwyn, 1817) |  |
|  | *Pirothyris vercoi* (Blochmann, 1910) | *Magasella vercoi* Blochmann (Dall, 1920) |
|  | *Platidia clepsidra* Cooper, 1973 |  |
|  | *Platidia davidsoni* (Deslongchampa 1855) |  |
|  | *Platidia anomioides* (Scacchi et Philippi, 1844 *in* Philippi, 1844) | *Platidia marionensis* (Cooper, 1973b, 1981a, Simoes et al., 2004, Logan, 2007, Zezina, 2010, Peck and Harper (2010), BIOCEAN database); *Platidia concentrica* (Zezina, 1980) (Zezina, 1985, 2010, Logan, 2007); |
|  | *Pumilus antiquatus* Atkins, 1958 |  |
|  | *Rectocalathis schemmgregoryi* Seidel & Lüter, 2014 |  |
|  | *Rhytirhynchia sladeni* (Dall, 1910) | *Rhytirhynchia sladeni* (Dall, 1910) *Rhytirhynchia sladeni*? (Cooper, 1973b); *Hemithyris sladeni* Dall (Dall, 1920) |
|  | *Septicollarina oceanica* Zezina, 1990 |  |
|  | *Septicollarina zezinae* Bitner, 2009 |  |
|  | *Septicollarina hemiechinata*Zezina, 1981 |  |
|  | *Shimodaia macclesfieldensis* MacKinnon et Long, 2009 |  |
|  | *Shimodaia pterygiota* MacKinnon, Saito et Endo, 1997 |  |
|  | *Simpliciforma profunda* Bitner et Zezina, 2013 *in* Bitner *et al*. (2013) |  |
|  | *Simplicithyris japonica* (Dall, 1920) | *Platidia japonica* (Dall, 1920, Hatai, 1936a, Zezina, 1970); *Amphithyris japonica* (Dall, 1920) (Zezina, 1981) |
|  | *Simplicithyris kurilensis* Zezina, 1976 |  |
|  | *Stenosarina crosnieri* (Cooper, 1983) | *Dallithyris (Dallithyris) crosnieri* (Cooper, 1983) (Zezina, 2010) |
|  | *Stenosarina globosa* Laurin, 1997 | *Dallithyris (Stenosarina) globosa* (Laurin, 1997) (Zezina, 2010) |
|  | *Stenosarina lata* Laurin, 1997 | *Dallithyris (Stenosarina) lata* (Laurin, 1997) (Zezina, 2010) |
|  | *Stenosarina nitens* Cooper, 1977 | *Dallithyris (Stenosarina) nitens* (Cooper, 1977) (Zezina, 2010) |
|  | *Stenosarina oregonae* Cooper, 1977 | *Dallithyris (Stenosarina) oregonae* (Cooper, 1977) (Zezina, 2010) |
|  | *Stenosarina parva* Cooper, 1977 |  |
|  | *Stenosarina sphenoidea* (Philippi, 1844) | *Dallithyris (Dallithyris) sphenoidea* (Philippi, 1844) (Zezina, 2010); *Dallithyris sphenoidea* (Philippi, 1844) (Zezina, 1985); *Dallithyris*? Aff D *sphenoides* (Peck and Harper (2010)); *Gryphus sphenoideus*(Philippi, 1844) (Dall, 1920); *Liothyris' spenoida* Philippi (Peck and Harper (2010)) *Stenosarina davidsoni* (Logan, 1998); *Gryphus sphenoideus*(Philippi, 1844) |
|  | *Stenosarina angustata* Cooper, 1977 | *Dallithyris (Stenosarina) angustata* (Cooper, 1977) (Zezina, 2010) |
|  | *Striarina valdiviae* (Helmcke, 1940) | *Rhynchonella* (=*Striarina) valdiviae* Helmcke (Cooper, 1973b) |
|  | *Syntomaria curiosa* Cooper, 1982 |  |
|  | *Terebratalia coreanica* (Adams et Reeve, 1850) |  |
|  | *Terebratalia gouldi* (Dall, 1891) | *Terebratella gouldii* (Dall, 1871) (Peck and Harper (2010)) |
|  | *Terebratalia xanthica* (Dall, 1920) | *Terebratalia (Pacificus) xanthica* Dall, 1920 (Zezina, 1981); *Terebratalia (Pacifithyris) xanthica* Dall, 1920. (Zezina, 2010) |
|  | *Terebratalia transversa* (Sowerby, 1846) | *Terebratalia caurina* (Dall, 1920, Hatai, 1942); *Terebratalia rubescens* Dall (Hatai, 1942) |
|  | *Terebratella crenulata* Sowerby, 1846 | *Magasella crenulata* Sowerby, 1846 (Zezina, 1985, 1990) |
|  | *Terebratella dorsata* (Gmelin, 1790) | *Terebratella submutica* Fischer and Oehlert, 1892 (Zezina, 1981); *Magasella flexuosa* King (Dall, 1920); *Magasella patagonica* (Davidson, 1887); *Terebratella enzenspergeri* Blochmann, 1906 (Cooper, 1981a, Jackson, 1918, Cooper, 1973b, 1981a, Zezina, 1985, Foster, 1974, Peck and Harper (2010)); *Terebratella (T.) rubiginosa* Dall, 1871 (Zezina, 2010); *Terebratella sowerbii* King (Dall, 1920) |
|  | *Terebratella tenuis* Tort, 2003 |  |
|  | *Terebratulina abyssicola* (Adams et Reeve, 1850) |  |
|  | *Terebratulina australis* Bitner, 2006 |  |
|  | *Terebratulina austroamericana* Zezina, 1981 |  |
|  | *Terebratulina cailleti* Crosse, 1865 |  |
|  | *Terebratulina callinome* Dall, 1920 |  |
|  | *Terebratulina cavata* Verco, 1910 |  |
|  | *Terebratulina compressa* Cooper, 1973 |  |
|  | *Terebratulina crossei* Davidson, 1882 |  |
|  | *Terebratulina hataiana* Cooper, 1973 |  |
|  | *Terebratulina hawaiiensis* Dall, 1920 |  |
|  | *Terebratulina japonica* (Sowerby, 1846) | *Terebratulina cumingii*Davidson, 1852 (Zezina, 1985, 2010) |
|  | *Terebratulina kiiensis* Dall et Pilsbry, 1891 | *Terebratulina magalhaenica* (Cooper, 1982) |
|  | *Terebratulina kitakamiensis* Hayasaka, 1938 |  |
|  | *Terebratulina kyusyuensis* Yabe et Hatai, 1934 | *Terebratulina (Shurugathyris) shurugaensis* Yabe et Hatai, 1934 (Hatai, 1936a, Zezina, 2010) |
|  | *Terebratulina meridionalis* Jackson, 1952 |  |
|  | *Terebratulina pacifica* Yabe et Hatai, 1934 |  |
|  | *Terebratulina photina* Dall, 1920 |  |
|  | *Terebratulina radula* Hedley, 1904 | *Cancellothyris radula* (Peck and Harper (2010), Harper and Peck (2016)) |
|  | *Terebratulina reevei* Dall, 1920 |  |
|  | *Terebratulina septentrionalis* (Couthouy, 1838) |  |
|  | *Terebratulina unguicula* (Carpenter, 1864) | *Terebratulina unguicula rotundata* (Cooper, 1973c) |
|  | *Terebratulina valdiviae* Blochmann, 1908 | *Terebratalia valvidia* (Harper and Peck (2016)) |
|  | *Terebratulina retusa* (Linné, 1758) | *Terebratulina retusa emarginata* (Risso, 1826) (Dall, 1920, Cooper, 1981b, Peck and Harper (2010), Zezina, 2010); *Terebratulina caputserpentis* abbreviata Jackson (Cooper, 1973b; Emig et al. 2015) |
|  | *Tethyrhynchia mediterranea*Logan 1994, *in* Logan & Zibrowius (1994) |  |
|  | *Thaumatosia anomala* Cooper, 1973 |  |
|  | *Thecidellina bahamiensis* Lüter, Hoffmann et Logan, 2008 |  |
|  | *Thecidellina blochmanni*Dall, 1920 |  |
|  | *Thecidellina congregata* Cooper, 1954 |  |
|  | *Thecidellina europa* Logan, Hoffmann et Lüter, 2015 |  |
|  | *Thecidellina insolita* Hoffmann et Lüter, 2010 |  |
|  | *Thecidellina japonica* (Hayasaka, 1938) |  |
|  | *Thecidellina leipnitzae* Simon, Hiller, Logan et Mottequin, 2019 |  |
|  | *Thecidellina mawaliana* Simon, Lüter, Logan et Mottequin, 2018 |  |
|  | *Thecidellina maxilla* (Hedley, 1899) | *Thecidea maxilla* Hedley, 1899 (Peck and Harper (2010)) |
|  | *Thecidellina meyeri* Hoffmann et Lüter, 2008 |  |
|  | *Thecidellina williamsi* Lüter, Hoffmann et Logan, 2008 |  |
|  | *Thecidellina barretti*(Davidson, 1864) |  |
|  | *Tichosina abrupta* Cooper, 1977 |  |
|  | *Tichosina bahamiensis* Cooper, 1977 |  |
|  | *Tichosina bartletti* (Dall, 1882) | *Gryphus bartlettii* Dall, 1882 (Dall, 1920, Logan, 2007); *Eurysina? bartletti* (Dall) (Cooper, 1983); *Liothyrella bartletti* (Zezina, 1981) |
|  | *Tichosina bartschi* (Cooper, 1934) |  |
|  | *Tichosina cubensis* (Pourtalès, 1867) | *Gryphus Cubensis* Pourtales (Dall, 1920); *Dallithyris cubensis* (Zezina, 1976, 1981) |
|  | *Tichosina elongata*Cooper, 1977 |  |
|  | *Tichosina erecta* Cooper, 1977 |  |
|  | *Tichosina expansa* Cooper, 1977 |  |
|  | *Tichosina labiata* Cooper, 1977 | *Eurysina labiata* (Cooper) (Cooper, 1983) |
|  | *Tichosina martinicensis* Cooper, 1977 |  |
|  | *Tichosina obesa* Cooper, 1977 | *Eurysina obesa* (Cooper) (Cooper, 1983) |
|  | *Tichosina pillsburyae* Cooper, 1977 |  |
|  | *Tichosina plicata* Cooper, 1977 | *Eurysina plicata* (Cooper) (Cooper, 1983); *Tichosina bullisi* Cooper, 1977 (Zezina, 1985, Cooper, 1977, Logan, 2007, Peck and Harper (2010)); *Eurysina bullisi* (Cooper) (Cooper, 1983); *Tichosina dubia* Cooper, 1977 (Zezina, 1985, Cooper, 1977, Logan, 2007, Peck and Harper (2010)); *Eurysina dubia* (Cooper) (Cooper, 1983) |
|  | *Tichosina rotundovata* Cooper, 1977 | *Tichosina ovata* (Cooper, 1977) (Cooper, 1977, 1983, Peck and Harper (2010) Logan, 2007, Zezina, 1985, 2010, Santagata et al., 2009); *Eurysina ovata* (Cooper) (Cooper, 1983) |
|  | *Tichosina solida* Cooper, 1977 | *Eurysina solida* (Cooper) Cooper, 1983 |
|  | *Tichosina subtriangulata*Cooper, 1977 |  |
|  | *Tichosina truncata* Cooper, 1977 |  |
|  | *Tichosina floridensis* Cooper, 1977 |  |
|  | *Tythothyris rosimarginata* Zezina, 1979 |  |
|  | *Valdiviathyris quenstedti* Helmcke, 1940 |  |
|  | *Xenobrochus agulhasensis* (Helmcke, 1939) | *Gryphus agulhasensis* Helmcke (Zezina, 1970 (1965?)); *Liothyrella agulhasensis* (Helmcke, 1938) (Cooper, 1973b); *Xenobrochus* ? *agulhasensis* (Helmcke, 1938) (Logan, 2007, Zezina, 2010) |
|  | *Xenobrochus anomalus* Cooper, 1981 |  |
|  | *Xenobrochus australis* Cooper, 1981 |  |
|  | *Xenobrochus indianensis* (Cooper, 1973) | *Gryphus indianensis* (Cooper, 1973b, Peck and Harper (2010)); *Liothyrella indianensis* (Cooper, 1973) (Zezina, 1981) |
|  | *Xenobrochus naudei* Hiller, 1994 |  |
|  | *Xenobrochus norfolkensis* Bitner, 2011 |  |
|  | *Xenobrochus parvus* (Cooper, 1977) | *Abyssothyris? parva* Cooper, 1977; *Xenobrochus? parvus* (Cooper, 1977) (Cooper, 1983; Zezina, 2010; Bitner & Molodtsova, 2020) |
|  | *Xenobrochus rotundus*Bitner, 2008 |  |
|  | *Xenobrochus translucidus*(Dall, 1920) | *Gryphus translucidus* Dall, 1920 (Dall, 1920, Peck and Harper (2010)); *Liothyrella translucida* (Dall, 1920) (Zezina, 1981) |
|  | *Xenobrochus africanus* (Cooper, 1973) | *Gryphus africanus* (Cooper, 1973b, Peck and Harper (2010)); *Liothyrella africana* (Cooper, 1973) (Zezina, 1981) |
|  | *Xenobrochus? affinis* | *Gryphus affinis* Calcara (Dall, 1920); *Liothyrella affinis* (Calcara, 1845); *Gryphus affinis* Calcara (Dall, 1920) |
|  | *Zygonaria davidsoni* (Adams, 1867) | *Gryphus davidsoni* A. Adams (Dall, 1920); *Liothyrella davidsoni* (Adams, 1867) (Zezina, 1981, 1985) |
|  | *Zygonaria joloensis* (Dall, 1920) | *Gryphus joloensis* (Dall, 1920, Zezina, 1985) |
