# Supplementary material for: Global biogeography of living brachiopods: Bioregionalization patterns and possible controls
Source: PLoS One. 2021 Nov 8;16(11):e0259004. doi: 10.1371/journal.pone.0259004 (PMC8575269; doi:10.1371/journal.pone.0259004)
Supplement: S2 Table — (DOCX) [file pone.0259004.s008.docx]

Supplementary Table 2: all available literature included in this research.

|  | References |
| --- | --- |
| Albano & Stockinger, 2017 | Paolo G. Albano & Martina Stockinger. 2017. Brachiopoda in a *Posidonia oceanica* meadow in Plakias, SW Crete, Greece |
| Alcock, 1894 | Alcock, A. 1894. A new brachiopod. Natural History notes from the Royal Indian Marine Survey steamer “Investigator,” Commander C. F. Oldham, R.N., commanding. Journal of the Asiatic Society of Bengal 63 (series II, no. 13):139–140. |
| Aliani, 1994 | Aliani, Stefano. 1994. I fondi a *Gryphus vitreus* (Brachiopoda) dell’arcipelago Toscano (Mediterraneo occidentale). Atti del 10° Congresso della Associazione Italiana di Oceanologia e Limnologia, Alassio, 4–6 November, 1992. Genova. p. 209–220. |
| Álvarez & Emig, 2005 | Álvarez, Fernando, and C. C. Emig. 2005. Brachiopoda. In F. Alvarez, C.C. Emig, C. Roldan, & J. M. Vieitez, Lophophorata, Phoronida, Brachiopoda, in A. Ramos and others, eds., Fauna Ibérica. Museo Nacional de Ciencias Naturales, Consejo Superior de Investigaciones Cientificas. Madrid. p. 57–177. |
| Álvarez et al., 2017 | Álvarez F., Emig C.C. and J. Tréguier, 2017. Brachiopodes actuels. Historique et révision de la collection D.­‐P. OEhlert (Laval). Brachiopodes des côtes françaises métropolitaines. Carnets de Géologie, Madrid, CG2017_B02, 386 p. |
| Anadón, 1994 | Anadón, Nuria. 1994. Braquiópodos actuales de la plataforma y talud continental de la costa central de Asturias (norte de España). Boletín de la Real Sociedad Española de Historia Natural, Sección Biológica 91(1-4):65–77. |
| Antoniadou, 1998 | Antoniadou C. (1998). Macro- and megafauna from the North Aegean Sea from 1997-1998. Aristotle University of Thessaloniki, Department of Biology, Laboratory of Zoology, Greece. |
| Asgaard & Bromley, 1991 | Asgaard U. and R. G. Bromley, 1991. Colonization by micromorph brachiopods in the shallow subtidal of the eastern Mediterranean Sea. In: Brachiopods through time, MacKinnon D. I., Lee D. E. & J. D. Campbell Eds, Balkema, Rotterdam, pp. 261-264. |
| Asgaard & Stentoft, 1984 | Asgaard, Ulla, and Niels Stentoft. 1984. Recent micromorph brachiopods from Barbados: Palaeoecological and evolutionary implications. Geobios (mémoire special) 8:29–33. |
| Atkins, 1958 | Atkins, Daphne. 1958. A new species and genus of Kraussinidae (Brachiopoda) with a note on feeding. Proceedings of the Zoological Society of London 131(4):559–581. |
| Atkins, 1959 | Atkins, Daphne. 1959. A new species of *Platidia* (Brachiopoda) from the La Chapelle Bank region. Journal of the Marine Biological Association of the United Kingdom 38:133–142. |
| Atkins, 1960a | Atkins, Daphne. 1960a. A new species and genus of Brachiopoda from the Western Approaches, and the growth stages of the lophophore. Journal of the Marine Biological Association of the United Kingdom 39:71–89. |
| Atkins, 1960b | Atkins, Daphne. 1960b. A note on *Dallina septigera* (Lovén), (Brachiopoda, Dallinidae). Journal of the Marine Biological Association of the United Kingdom 39:91–99. |
| Atkins, 1960c | Atkins, Daphne. 1960c. The ciliary feeding mechanism of the Megathyridae (Brachiopoda), and the growth stages of the lophophore. Journal of the Marine Biological Association of the United Kingdom 39:459–479. |
| Atkins, 1961a | Atkins, Daphne. 1961a. The generic position of the brachiopod *Megerlia echinata* (Fischer & Oehlert). Journal of the Marine Biological Association of the United Kingdom 41:89–94. |
| Atkins, 1961b | Atkins, Daphne. 1961b. The growth stages and adult structure of the lophophore of the brachiopods *Megerlia truncata* (L.) and *M. echinata* (Fischer & Oehlert). Journal of the Marine Biological Association of the United Kingdom 41:95–111. |
| Australian Zooplankton Database | The Australian Zooplankton Database (1938 onwards) |
| Ayala et al., 1975 | Francisco J. Ayala, James W. Valentine, Ted E. Delaca and Gary S. Zumwalt. 1975. Genetic Variability of the Antarctic Brachiopod Liothyrella notorcadensis and Its Bearing on Mass Extinction Hypotheses. Journal of Paleontology 49:1-9. |
| Araya and Bitner, 2018 | Araya J. F. and Bitner M. A. 2018. Rediscovery of *Terebratulina austroamericana* Zezina, 1981 (Brachiopoda: Cancellothyrididae) from off Northern Chile. Zootaxa 4407:443-446. |
| Baird et al., 2013 | Baird M.J, Lee D.E, Lamare M.D. 2013. Reproduction and Growth of the Terebratulid Brachiopod *Liothyrella neozelanica* Thomson, 1918 from Doubtful Sound, New Zealand. Biological Bulletin 225:125-136. |
| Bajnai et al., 2018 | Bajnai, D., Fiebig, J., Tomašových, A. et al. 2018. Assessing kinetic fractionation in brachiopod calcite using clumped isotopes. Sci Rep 8, 533. https://doi.org/10.1038/s41598-017-17353-7 |
| Bernard, 1972 | Bernard, F. R. 1972. The living Brachiopoda of British Columbia. Syesis 5:73–82. |
| Besteiro & Urgorri, 1984 | Besteiro, C., and V. Urgorri. 1984. Sobre la presencia de *Argyrotheca cistellula* (Searles-Wood, 1841), *Gwynia capsula* (Jeffreys, 1859) (Brachiopoda, Terebratulida) y *Leptosynapta minuta* (Becher, 1906) (Holothuroidea, Apoda) en las costas gallegas. In V. M. Marques, ed., Actas do IVo Simpósio ibérico de estudos do benthos marinho, vol. 3, 2–25 May, 1984. Lisboa. p. 219–226. |
| BIOCEAN database | Fabri, M-C. et al., Ifremer BIOCEAN database (Deep Sea Benthic Fauna). Institut Français de Recherche pour l'Exploitation de la Mer, Ifremer, Issy-les-Moulineaux, France. World Wide Web electronic publication, http://www.ifremer.fr/isi/biocean |
| Bitner & Cahuzac, 2004 | Bitner M. A. and Bruno Cahuzac. 2004. The genus *Cryptopora* (Brachiopoda) from the Miocene of France and its history and biogeography. Geobios 37:1–12.. |
| Bitner & Cohen, 2015 | Bitner, M. A. and Cohen, B. L. 2015. Congruence and conflict: case studies of morphotaxonomy versus rDNA gene tree phylogeny among articulate brachiopods (Brachiopoda: Rhynchonelliformea), with description of a new genus. Zoological Journal of the Linnean Society 173: 486-504. |
| Bitner & Logan, 2016 | Bitner M. A. and Logan A. 2016. Recent Brachiopoda from the Mozambique-Madagascar area, western Indian Ocean. Zoosystema, 38:5-41. |
| Bitner & Molodtsova, 2018 | Bitner, M. A. and Molodtsova, T. N. 2018. Redescription of *Nanacalathis atlantica* Zezina, 1991 (Brachiopoda: Chlidonophoridae) from the North Atlantic. Marine Biodiversity 48: 995–999. |
| Bitner & Molodtsova, 2020 | Bitner, M.A. & Molodtsova, T.N. 2020. New records of Recent brachiopods (Terebratulida) from the northern Mid-Atlantic Ridge. *Marine Biology Research*, |
| Bitner & Romanin, 2018 | Bitner M. A. and Romanin M. 2018. Recent brachiopods collected during the ZhongSha 2015 expedition to the South China Sea West Pacific. Marine Biology Research, 14:551-564. |
| Bitner et al., 2013 | Bitner M. A. Melnik V. P. and Zezina O. 2013. New paedomorphic brachiopods from the abyssal zone of the north-eastern Pacific Ocean. Zootaxa 3613:281-288. |
| Bitner et al., 2008a | Bitner, M. A. Logan, A. and Gischler, E. 2008a. Recent brachiopods from the Persian Gulf and their biogeographical significance. Scientia Marina 72: 279-285. |
| Bitner et al., 2008b | Bitner, M. A., Cohen, B. L., Long, S. L., Richer de Forges, B., and Saito, M. 2008b. *Gyrothyris williamsi* sp. nov. and inter-relationships of some taxa from waters around New Zealand and the southern oceans (Rhynchonelliformea: Terebratelloidea). Earth and Environmental Science Transactions of the Royal Society of Edinburgh, 98, 425–435. |
| Bitner, 2006a | Bitner M. A. 2006a. First record of brachiopods from the Marquesas Islands, French Polynesia, south central Pacific. Pacific Science 60: 417–424. |
| Bitner, 2006b | Bitner M. A. 2006b. Recent Brachiopoda from the Fiji and Wallis and Futuna Islands, south-west Pacific. In B. Richer de Forges & J.-L. Justine, eds., Tropical Deep-Sea Benthos, vol. 24. Mémoires du Muséum national d’Histoire naturelle 193:15–32. |
| Bitner, 2007 | Bitner M. A. 2007. Recent brachiopods from the Austral Islands, French Polynesia, South Central Pacific. Zoosystema 29: 491-502. |
| Bitner, 2008 | Bitner M. A. 2008. New data on the recent brachiopods from the Fiji and Wallis and Futuna islands, South-West Pacific. Zoosystema 30 (2): 419-461. |
| Bitner, 2010 | Bitner M. A. 2010. Biodiversity of shallow-water brachiopods from New Caledonia, SW Pacific, with description of a new species. Scientia Marina, 74: 643-657. |
| Bitner, 2011 | Bitner M. A. 2011. *Xenobrochus norfolkensis* (Brachiopoda Dyscoliidae), a new species from the Norfolk Ridge, New Caledonia, South-West Pacific. Carnets de Géologie / Notebooks on Geo-logy, Brest, Article 2011/05 (CG2011_A05), p. 203-211. |
| Bitner, 2014 | Bitner M. A. 2014. Living Brachiopods from French Polynesia, Central Pacific, with Descriptions of Two New Species. Pacific Science, 68:245-265. |
| Bitner, 2015 | Bitner M. A. 2015. Checklist of recent brachiopod species collected during the Terrasses and Exbodi cruises in the New Caledonian region, SW Pacific. Zookeys. 537: 33–50. |
| Bitner, 2019 | Bitner M. A. 2019. Recent brachiopods from the Tonga islands, SW Pacific taxonomy and biogeography. Proceedings of the 8th International Brachiopod Congress. 125: 587-608. |
| Blochmann, 1906 | Blochmann, F. 1906. Neue Brachiopoden der Valdivia-und Gaussexpeditionen. Zoologischer Anzeiger30(21-23):690–702. |
| Blochmann, 1908 | Blochmann, F. 1908. Zur Systematik und geographischen Verbreitung der Brachiopoden. Zeitschrift für wissenschaftliche Zoologie 90:596–644. |
| Blochmann, 1910 | Blochmann, F. 1910. New brachiopods from South Australia. Transactions of the Royal Society of South Australia 34:90–99. |
| Blochmann, 1914 | Blochmann, F. 1914. Some Australian brachiopods. Papers and Proceedings of the Royal Society of Tasmania for 1913:112–115. |
| Blom & Moriarty, 2018 | Blom W, Moriarty A (2018). Auckland Museum NZ Marine Collection. Version 1.11. Auckland War Memorial Museum. Occurrence Dataset https://doi.org/10.15468/plyefd accessed via GBIF.org on 2018-01-15 |
| Bousfield, 1960 | Bousfield, E. L. 1960. Canadian Atlantic Sea Shells. National Museum of Canada. Ottawa. 72 p., 13 pl. |
| Bowen, 1968 | Bowen, Z. P. 1968. A guide to New Zealand Recent brachiopods. Tuatara 16(2):127–150. |
| Branch, 2002 | Branch (2002) West Coast biodiversity survey. University of Cape Town. Published by AfrOBIS; consulted via iOBIS. |
| Brand et al., 2003 | Brand U. Logan A. Hiller N. Richardson J. 2003. Geochemistry of modern brachiopods: applications and implications for oceanography and paleoceanography. Chemical Geology 198: 305-334. |
| Brunton & Curry, 1979 | Brunton, C. H. C., and G. B. Curry. 1979. British Brachiopods. In D. M. Kermack & R. S. K. Barnes, eds., Synopses of the British Fauna (new series) 17:64 p., 30 |
| Brunton, 1988 | Brunton, C. H. C. 1988. Some brachiopods from the eastern Mediterranean Sea. Israel Journal of Zoology 35:151–169. |
| Brunton, 1996 | Brunton, C. H. C. 1996. The functional morphology of the Recent brachiopod *Bouchardia rosea*. Acta Zoologica 77:233–240. |
| Buhl-Mortensen, 2014 | Buhl-Mortensen, L. (2014). MAREANO - Base-line mapping of hyperbenthic crustacea fauna obtained with RP-sledge. Institute of Marine Research, Norway https://doi.org/10.15468/gecvl4 |
| Cals & Emig, 1979 | Cals, P. and Emig, C. C. 1979. Lingules d'amboine, *Lingula reevii* Davidson et *Lingula rostrum* (Shaw), données écologiques et taxonomiques concernant les problèmes de speciation et de repartition. Cah. Indo-pacifique; Fra 2: 153-164. |
| Came et al., 2014 | Came R. E., Brand U., Affek H. P. 2014. Clumped isotope signatures in modern brachiopod carbonate. Chemical Geology 377: 20-30. |
| Campbell & Fleming, 1981 | Campbell, J. D., and C. A. Fleming. 1981. Brachiopoda from Fiordland, New Zealand, collected during the Golden Hind Expedition, 1946. New Zealand Journal of Zoology 8:145–155. |
| CeDAMar database | Martinez Arbizu, P. Smith, C. R., Keller, S. & Ebbe, B. (Editors). Biogeographic Database of the Census of Abyssal Marine Life. [date accessed]. World Wide Web electronic publication. Available online at http://www.cedamar.org/biogeography https://doi.org/10.15468/oc9tsb |
| Chapman & Richardson, 1981 | Chapman, B. E., and J. R. Richardson. 1981. Recent species of *Neothyris* (Brachiopoda: Terebratellinae). New Zealand Journal of Zoology 8:157–161. |
| Clark, 1976 | W.C. Clark (1976) The genus *Rhynchothorax costa* (Pycnogonida) in New Zealand waters, Journal of the Royal Society of New Zealand, 6:3, 287-296, DOI: 10.1080/03036758.1976.10421476 |
| Cohen et al., 1991 | Cohen, B. L., Peter Balfe, Moyra Cohen, and G. B. Curry. 1991. Genetic divergence within and between populations of the North Atlantic morphospecies *Terebratulina retusa* and *T. septentrionalis*. In D. I. MacKinnon, D. E. Lee, & J. D. Campbell, eds., Brachiopods through Time. Proceedings of the 2nd International Brachiopod Congress, University of Otago, Dunedin, New Zealand, 5-9 February, 1990. Balkema. Rotterdam. p. 109–115. |
| Cohen et al., 1993 | Cohen, B. L., Peter Balfe, Moyra Cohen, & G. B. Curry. 1993. Molecular and morphometric variation in European populations of the articulate brachiopod *Terebratulina retusa*. Marine Biology 115:105–111. |
| Cohen et al., 2011 | Cohen, B. L. Bitner, M. A. Harper, E. M. Lee, D. E. Mutschke, E. and Sellanes, J. 2011. Vicariance and convergence in Magellanic and New Zealand long-looped brachiopod clades (Pan-Brachiopoda: Terebratelloidea). Zoological Journal of the Linnean Society, 162: 631–645. |
| Cooper & Doherty, 1993 | G. A. Cooper & P. J. Doherty (1993) *Calloria variegata*, a new Recent species of brachiopod (Articulata: Terebratulida) from northern New Zealand, Journal of the Royal Society of New Zealand, 23:3, 271-281, DOI: 10.1080/03036758.1993.10721225 |
| Cooper & Lee, 1993 | Cooper, G. A., and D. E. Lee. 1993. *Calloria*, a replacement name for the Recent brachiopod genus *Waltonia* from New Zealand. Journal of the Royal Society of New Zealand 23(3):257–270. |
| Cooper, 1934 | Cooper, G. A. 1934. New brachiopods. Reports on the collections obtained by the first Johnson-Smithsonian deep-sea expedition to the Puerto Rican Deep. Smithsonian Miscellaneous Collections 91(10):5 p., 2 |
| Cooper, 1954a | Cooper, G. A. 1954a. Brachiopoda occurring in the Gulf of Mexico. Fishery Bulletin of the Fish and Wildlife Service 55:363–365. |
| Cooper, 1954b | Cooper, G. A. 1954b. Recent Brachiopods. Bikini and nearby atolls, Marshall Islands. Geological Survey Professional Paper 260-G:315–318. |
| Cooper, 1957 | Cooper, G. A. 1957. Tertiary and Pleistocene brachiopods of Okinawa, Ryukyu Islands. United States Geological Survey Professional Paper 314-A:20. |
| Cooper, 1959 | Cooper, G. A. 1959. Genera of Tertiary and Recent rhynchonelloid brachiopods. Smithsonian Miscellaneous Collections 139(5):1–90. |
| Cooper, 1964 | Cooper, G. A. 1964. Brachiopods from Eniwetok and Bikini drill holes. Bikini and nearby atolls, Marshall Islands. United States Geological Survey Professional Paper 260-FF:1117–1120. |
| Cooper, 1972 | Cooper, G. A. 1972. Homeomorphy in Recent deep-sea brachiopods. Smithsonian Contributions to Paleobiology 11:25 p., 5 |
| Cooper, 1973a | Cooper, G. A. 1973a. Brachiopods (Recent). Memoirs of the Hourglass Cruises 3(3):17 p., 6 |
| Cooper, 1973b | Cooper, G. A. 1973b. New Brachiopoda from the Indian Ocean. Smithsonian Contributions to Paleobiology 16:43 p., 1 |
| Cooper, 1973c | Cooper, G. A. 1973c. Fossil and Recent Cancellothyridacea (Brachiopoda). Science Reports of the Tohoku University, Sendai, Japan, Second Series (Geology), Special Volume 6 (Hatai Memorial Volume):371–390, 2 |
| Cooper, 1973d | Cooper, G. A. 1973d. Vema’s Brachiopoda (Recent). Smithsonian Contributions to Paleobiology 17:51 p., 5 |
| Cooper, 1975 | Cooper, G. A. 1975. Brachiopods from West African waters with examples of collateral evolution. Journal of Paleontology 49:911–927, 7 |
| Cooper, 1977 | Cooper, G. A. 1977. Brachiopods from the Caribbean Sea and adjacent waters. Studies in Tropical Oceanography no. 14. Rosenstiel School of Marine and Atmospheric Science, University of Miami. Miami. 212 p., 8 |
| Cooper, 1981a | Cooper, G. A. 1981a. Brachiopoda from the southern Indian Ocean (Recent). Smithsonian Contributions to Paleobiology 43:93 p., 30 |
| Cooper, 1981b | Cooper, G. A. 1981b. Brachiopoda from the Gulf of Gascogne, France (Recent). Smithsonian Contributions to Paleobiology 44:35 p., 5 |
| Cooper, 1983 | Cooper, G. A. 1983. The Terebratulacea (Brachiopoda), Triassic to Recent: A study of the brachidia (loops). Smithsonian Contributions to Paleobiology 50:445 p., 17 fig., 77 pl. |
| Cooper, 1982 | Cooper, G. A. 1982. New Brachiopoda from the southern hemisphere and *Cryptopora* from Oregon (recent). *Smithsonian Contributions to Paleobiology.* 41:1–43 |
| Craig, 1952 | Craig G. Y. 1952. A comparative study of the ecology and palaeoecology of *Lingula*. Trans. Edinb. Geol. Soc., 15, 110-120. |
| Cross et al., 2016 | Cross E. L., Peck L. S., Lamare M. D., Harper E. M. 2016. No ocean acidification effects on shell growth and repair in the New Zealand brachiopod *Calloria inconspicua* (Sowerby, 1846). ICES Journal of Marine Science 73: 920-926. |
| CSIRO - Southern Surveyor voyage SS 01/2000 | CSIRO - Southern Surveyor voyage SS 01/2000, SE large marine domain and the Great Australian Bight |
| CSIRO - Southern Surveyor voyage SS 10/2005 | CSIRO - Southern Surveyor voyage SS 10/2005, benthic biodiversity of the deep continental shelf and slope in Australia's SW region |
| CSIRO Marine Invertebrates Image Collection | CSIRO Marine Invertebrates Image Collection (MIIC) |
| CSIRO, Cruise SS200404 | CSIRO, Cruise SS200404, Benthic Biodiversity, Tasmanian Coast; Bass Strait; Tasman Sea; NSW Coast, 2004 |
| CSIRO, Cruise SS200803 | CSIRO, Cruise SS200803, Marine Biological Survey, Great Australian Bight; South Australian and Tasmanian continental slope, 2008 |
| Curry, 1982 | Curry, G. B. 1982. Ecology and population structure of the Recent brachiopod *Terebratulina* from Scotland. Palaeontology 25(2):227–246. |
| Cusson, 2018 | Cusson M. 2018. Biodiversity of benthic assemblages on the Arctic continental shelf: historical data from Canada (1955 to 1977). v1.2. Canadian node of the Ocean Biogeographic Information System (OBIS Canada). Dataset/Occurrence. http://ipt.iobis.org/obiscanada/resource?r=cusson_arcticbenthos&v=1.2 |
| d’Hondt, 1973 | d’Hondt, Jean-Loup. 1973. Bryozoaires et Brachiopodes de la Campagne Noratlante. Bulletin du Muséum national d’Histoire naturelle (series 3, no. 179) Zoologie 118:1209–1223. |
| d’Hondt, 1976 | d’Hondt, Jean-Loup. 1976. Sur quelques Brachiopodes actuels (océan Atlantique, Mediterranée, Kerguelen). Bulletin du Muséum national d’Histoire naturelle (series 3, no. 350) Zoologie 243:1–13. |
| d’Hondt, 1987 | d’Hondt, Jean-Loup. 1987. Observations sur les Brachiopodes actuels de Nouvelle-Calédonie et d’autres localités de l’Indo-Pacifique. Bulletin du Muséum national d’Histoire naturelle (series 4) 9(section A, no. 1):33–46. |
| Dahle & Cochrane, 1992 | Dahle S., S. Cochrane, 1992: Northern Barents Sea 1992. Akvaplan-niva, Norway |
| Dahle et al., 1992 | Dahle S., R. Palerud, N. Anisimova, 1992: Benthic fauna around Franz Josef Land. Akvaplan-niva, Norway. |
| Dall, 1871 | Dall, W. H. 1871. Report on the Brachiopoda obtained by the United States Coast Survey Expedition, in charge of L.F. de Pourtalès, with a revision of the Craniidae and Discinidae. Bulletin of the Museum of Comparative Zoology, Harvard University 3(1):1–45. |
| Dall, 1895 | Dall, W. H. 1895. Report on Mollusca and Brachiopoda dredged in deep water, chiefly near the Hawaiian Islands, with illustrations of hitherto unfigured species from northwest America. Scientific results of explorations by the U.S. Fish Commission Steamer Albatross. Proceedings of the United States National Museum 17:675–733. |
| Dall, 1910 | Dall, W. H. 1910. Report on the Brachiopoda obtained from the Indian Ocean by the Sealark Expedition, 1905. *Transactions Linnaean Society London (series 2, Zoology).* 13(3):439–441. |
| Dall, 1920 | Dall, W. H. 1920. Annotated list of the recent Brachiopoda in the collection of the United States National Museum, with descriptions of thirty-three new forms. Proceedings of the United States National Museum 57(2314):261–377. |
| Danish Deep Sea Expedition, 1950-52 | The Danish Biodiversity Information Facility, Galathea II, Danish Deep Sea Expedition 1950-52 https://doi.org/10.15468/ouseij |
| Davidson, 1880 | Davidson, Thomas. 1880. Report on the Brachiopoda dredged by H.M.S. Challenger during the years 1873-76. In Report of the Scientific Results of the Voyage of H.M.S. Challenger during the years 1873–1876. Zoology 1:67. |
| Davidson, 1887 | Davidson, Thomas. 1886–1888. A monograph of Recent Brachiopoda. The Transactions of the Linnean Society of London. Second Series, Volume IV, Zoology. Part I, 1886:1–74, 13 pl., Part II, 1887:75–182, 12 pl., Part III, 1888:183–248. |
| Dawson, 1971 | Dawson, E. W. 1971. A reference list and bibliography of the Recent Brachiopoda of New Zealand. Journal of the Royal Society of New Zealand 1(2):159–174. |
| Dawson, 1991 | Dawson E. W., 1991. The systematics and biogeography of the living Brachiopoda of New Zealand. In: Brachiopods through time, MacKinnon D. I., Lee D. E. & J. D. Campbell Eds, Balkema, Rotterdam, pp. 431-437. |
| Deudero et al., 2011 | Deudero, Vallespir, Obrador 2011. Atlas de Biodiversidad Marina del Mar Balear. http://www.ba.ieo.es |
| DFO, 2016 | DFO. (2016). Central and Arctic Multi-Species Stock Assessment Surveys Version 6 In OBIS Canada Digital Collections. Bedford Institute of Oceanography, Dartmouth, NS, Canada. Published by OBIS, Digital http://www.iobis.org/. Accessed on –INSERT DATE |
| d'Hondt & Gordon, 1996 | d'Hondt, J.-L. and D.P. Gordon. 1996. Bryozoa: Ctenostomes Et Cheilostomes (Cellularines, Scrupariines Et Malacosteges) Des Campagnes Musorstom Autour De La Nouvelle-Caledonie. Memoires du Museum National d'Histoire Naturelle 168: 55-59. As viewed in K. Stocks, SeamountsOnline 5 June 2010. http://seamounts.sdsc.edu. |
| Di Geronimo et al., 2001 | Di Geronimo, I., R. La Perna, A. Rosso, R. Sanfilippo, & E. Taddei Ruggieri. 2001. Associazioni bentoniche da sedimenti di grotto carsiche in Sicilia. Atti del 1o Seminario di Studi sul il Carsismo negli Iblei e nell’area sud Mediterranea, Ragusa, 9–11 aprile, 1999. Speleologia Iblea 8:97–102. |
| Doherty, 1979 | Doherty, P. J. 1979. A demographic study of a subtidal population of the New Zealand articulate brachiopod *Terebratella inconspicua*. Marine Biology 52:331–342. |
| Donovan & Gale, 1990 | Donovan S. K. and Gale A.S. 1990. Predatory asteroids and the decline of the articulate brachiopods. Lethaia 23: 77-86. |
| Du Bois, 1916 | Du Bois H. M., 1916. Variation induced in brachiopods by environmental conditions.Publ. Puget Sd Mar. Sta. 1 (16): 177–188. |
| Emig & Hammond, 1981 | Emig, C. C. and Hammond L. S. 1981. On the status of *Lingula tumidula* Reeve, *L. adamsi* Dall, and *L. shantungensis* Hatai (Brachiopoda, Inarticulata). Bulletin of Marine Science, 31: 37-45. |
| Emig, 1977a | Emig, C. C. 1977a. *Lingula murphiana* Reeve (Brachiopoda) recollée a Madagascar. Bull. Mus. Hisl. nal. Paris 446 (Zool. 309): 401-10. |
| Emig, 1977b | Emig, C. C. 1977b. Un nouvel embranchement: Les Lophophorates. Bulletin de la Société Zoologique de France 102:341–344. |
| Emig, 1983a | Emig, C. C. 1983a. Comportement expérimental de *Lingula anatina* (Brachipode, Inarticulé) dans diver substrats meubles (Blaie de Matsu. Japan). Marine Biology 75: 207-213. |
| Emig, 1983b | Emig, C. C. 1983b. Taxonomie du genre *Glottidia* (Brachiopodes, Inarticulés). Bulletin du Muséum national d'histoire naturelle. Section A, Zoologie, biologie et écologie animales. 5: 469-489. |
| Emig, 1985 | Emig, C. C. 1985. Distribution et synécologie des fonds à *Gryphus vitreus* (Brachiopoda) en Corse. Marine Biology 90:139–146. |
| Emig, 1987 | Emig, C. C. 1987. Offshore brachiopods investigated by submersible. Journal of Experimental Marine Biology and Ecology 108:261–273. |
| Emig, 1997a | Emig, C. C. 1997a. Ecology of inarticulated brachiopods, p. 473–495. In Kaesler, R. L. (ed.), Treatise on Invertebrate Paleontology, Part H, revised, Brachiopoda, Volume 1. Geological Society of America and University of Kansas Press, Lawrence. |
| Emig, 1997b | Emig, C. C. 1997b. "Biogeography of the Inarticulated Brachiopods." In Treatise on Invertebrate Paleontology, Part H revised. Brachiopoda, vol. 1, edited by R. L. Kaesler. Boulder, CO, and Lawrence, KS: Geological Society of America and University of Kansas, 1997. |
| Emig, 1999 | Emig, C. 1999. Brachiopoda from sampling campaigns in the French part of the Mediterranean during the 1970-1990s. Centre National de la Recherche Scientifique; Institut National de Science de l'Univers; Centre d'Océanologie de Marseille; Station Marine d'Endoume, France. |
| Emig, 2008 | Emig, C. C. 2008. On the history of the names *Lingula anatina*, and on the confusion of the forms assigned them among the Brachiopoda. Carnets de Géologie / Notebooks on Geology, Brest, Article 2008/08 (CG2008_A08). |
| Emig, 2017 | Emig, C.C. 2017. Atlas of Antarctic and sub-Antarctic Brachiopoda.- Carnets de Géologie, Madrid, ISBN13: 978-2-916733-15-9, Book 2017/03 (CG2017_B03), 93 p. |
| Emig, 2018 | Emig, C. C. 2018. Brachiopodes récoltés lors de campagnes (1976-2014) dans l’étage Bathyal des côtes françaises méditerranéennes. Redéfinition des limites du système phytal dans le domaine marin benthique. Carnets de Géologie, Madrid, CG2018_B01, 100 p. |
| Emig et al, 2015 | Emig, C.C., Bitner, M.A. & Alvarez, F. 2015. Scientific death-knell of databases? Errors induced by database manipulations and its consequences. *Carnets Geol*. 15 (16), 321-328. |
| Endo & Curry, 1991 | Endo, K. and Curry G. B. 1991. Molecular and morphological taxonomy of a Recent brachiopod genus *Terebratulina*. In D. I. MacKinnon, D. E. Lee, & J. D. Campbell, eds., Brachiopods through Time. Proceedings of the 2nd International Brachiopod Congress, University of Otago, Dunedin, New Zealand, 5–9 February, 1990. Balkema. Rotterdam. p. 101–108. |
| Endo, 1987 | Endo, K., 1987. Life habit and relative growth of some laqueid brachiopods from Japan. Transactions and Proceedings of the Palaeontological Society of Japan. New Series, no. 147, pp. 180–194. |
| Eshleman & Wilkens, 1979 | Eshleman Q. P. and Wilkens J. L. 1979. Actomyosin ATPase activities in the brachiopod *Terebratalia transversa*. Canadian Journal of Zoology, 57: 1944-1949. |
| Fischer & Oehlert, 1892 | Fischer, P., AND D.-P. Oehlert. 1892. Brachiopodes. Mission scientifique du Cap Horn (1882–1883), Bulletin de la Société d’Histoire Naturelle d’Autun 5:82 p., 7 |
| Fischer & Oehlert, 1891 | Fischer, P., AND D.-P. Oehlert. 1891. Brachiopodes. Expéditions scientifiques du Travailleur et du Talisman pendant les années 1880, 1881, 1882, 1883. Masson. Paris. 139 p., 15 |
| Flanders Marine Institute, 2004 | Flanders Marine Institute (VLIZ). Taxonomic Information System for the Belgian coastal area. 10 Aug 2004, Oostende, Belgium, |
| Forbes, 1844 | Forbes, Edward. 1844. Report on the Mollusca and Radiata of the Aegean Sea, and on their distribution, considered as bearing on geology. Report of the British Association for the Advancement of Science, 1843, Palliobranchiata. London. p. 130–147. |
| Foster, 1969 | Foster, M. W. 1969. Distribution of selected groups of marine invertebrates in waters South of 35°S Latitude. Brachiopoda. Folio 11, Antarctic Map Folio Series, American Geographical Society. p. 21–22. |
| Foster, 1974 | Foster, M. W. 1974. Recent Antarctic and Subantarctic brachiopods. American Geophysical Union, Antarctic Research Series 21:189 p., 39 |
| Foster, 1989 | Foster, M. W. 1989. Brachiopods from the extreme South Pacific and adjacent waters. Journal of Paleontology 63(3):268–301. |
| Funaki, 2019 | Funaki S (2019). Brachiopods Specimens of Akita Prefectural Museum. National Museum of Nature and Science, Japan. Occurrence dataset https://doi.org/10.15468/zxenpz accessed via GBIF.org on 2020-02-27. https://www.gbif.org/occurrence/380236852 |
| Gaspard, 2003a | Gaspard, Danièle. 2003a. Some Cretaceous long-looped terebratulide brachiopods analysed in the light of the diversity observed in the ontogeny of Recent representatives. Bulletin Société géologique de France 174(3):261–269, 2 fig. |
| Gaspard, 2003b | 2003b. Gaspard. Recent brachiopods collected during the “SEAMOUNT 1” CRUISE off Portugal and the Ibero-Moroccan Gulf (Northeastern Atlantic) in 1987Brachiopodes actuels récoltés lors de la campagne Seamount |
| Goldsmit, Jesica. 2016 | Goldsmit, Jesica. 2016. CAISN: Abundance and biomass of benthic invertebrates collected in four ports of the Canadian Arctic during summers of 2011 and 2012. Version 2 In OBIS Canada Digital Collections. Bedford Institute of Oceanography, Dartmouth, NS, Canada. Published by OBIS, Digital http://www.iobis.org/. Accessed on –INSERT DATE |
| Grant, 1983 | Grant, R. R. 1983. *Argyrotheca arguta*, a new species of brachiopod from the Marshall Islands, western Pacific. Proceedings of the Biological Society of Washington 96(1):178–180. |
| Grant, 1987 | Grant, R. R. 1987. Brachiopods of Enewetak Atoll. In D. M. Devaney, E. S. Reese, B. L. Burch, & P. Helfrich, eds., The Natural History of Enewetak Atoll, vol. 2. Biogeography and Systematics. Office of Scientific and Technical Information, U.S. Department of Energy. Oak Ridge, Tennessee. p. 77–84. |
| Great Barrier Reef, 2003-2006 | CSIRO - Great Barrier Reef seabed biodiversity study 2003-2006 |
| Hammen & Lum, 1977 | Hammen C. S. and Lum S. C. 1977. Salinity tolerance and pedicle regeneration of *Lingula*. Journal of Paleontology 51:548-551. |
| Hammond, 1983 | Hammond L. S. 1983. Experimental studies of salinity tolerance, burrowing behavior and pedicle regeneration in *Lingula anatina* (Brachiopoda, Inarticulata). Journal of Paleontology 57:1311-1316. |
| Harper & Peck 2009 | Harper E. M. Peck L. S. Hendry K. R. 2009. Patterns of shell repair in articulate brachiopods indicate size constitutes a refuge from predation. Marine Biology, 156: 1993-2000. |
| Harper & Peck, 2016 | Harper E. M. and Peck L. S. 2016. Latitudinal and depth gradients in marine predation pressure. Global ecology and biogeography 25: 670-678. |
| Harper et al., 1996 | Harper, D. A. T., S. K. Donovan, and C. J. Veltkamp. 1996. The micromorphic articulate brachiopod *Gwynia* from the Western Approaches, U.K. Journal of Paleontology 70(2):331–333. |
| Harper, 2011 | Harper E. M. 2011 What do we really know about predation on modern rhynchonelliforms? Memoirs of the Association of Australasian Palaeontologists 41: 45-57. |
| Hassel, 2014 | Hassel, A. (2014). MAREANO - Base-line mapping of epifauna obtained with Beamtrawl. Institute of Marine Research, Norway https://doi.org/10.15468/iomgfj |
| Hatai, 1936a | Hatai K. M. 1936a. The geographic distribution of Brachiopoda. Part 1. Recent Brachiopoda of Japan. Bulletin of the Biogeographical Society of Japan 6(8):64–70. |
| Hatai, 1937 | Hatai K. M., 1937. On some Recent brachiopods from Eastern Shantung, China.Bulletin of the Biogeographical Society of Japan 7 (13): 318–324. |
| Hatai, 1940 | Hatai K. M. 1940. The Cenozoic Brachiopoda of Japan. Science Reports of the Tohuku Imperial University, Sendai, Japan (second series, Geology) 20:413 p., 26 |
| Hayward & Morley, 2004 | Bruce W Hayward and Margaret S. Morley (2004) Intertidal biota of the proposed Nga Motu Marine Reserve, New Plymouth. Poirieria 01/2002; 28:1-11. |
| Hayward et al., 1999 | Hayward, B.W., Morley, M.S., Blom, W., Grenfell, H., Prasad, R., Rogan, D., Thompson, F., Cheetham, J., and Webb, M. (1999) Intertidal and subtidal biota and habitats of the central Waitemata Harbour. Auckland Regional Council Technical Report 127. 36pp. |
| Hedley, 1906 | Hedley C. 1906. The Mollusca of Mast Head Reef, Capricorn Group, Queensland. Proceedings of the Linnean Society of New South Wales 31:453–467, 1 pl. |
| Helmcke, 1939 | Helmcke J. G. 1939. *Kraussina mercatori* n. sp. und die Verbreitung der Gattung Kraussina. Résultats scientifiques des croisières du Navire-École Belge “Mercator,” vol. 2. Mémoires Museum Royal d’Histoire naturelle de Belgique (series 2, fasc. 15) 11(10):135–139. |
| Helmcke, 1940 | Helmcke, J. G. 1940. Die Brachiopoden der deutschen Tiefsee-Expedition. Wissenschaftliche Ergebnisse der deutschen Tiefsee-Expedition auf dem Dampfer “Valdivia” 1898–1899, vol. 24, no. 3. G. Fischer Verlag. 1932–1940. Jena. p. 217–316, 40 fig. |
| Henkes et al., 2013 | Henkes G. A., Passey B. H., Wanamaker A. D., Grossman E. L., Ambrose W. G., Carroll M. L., 2013. Carbonate clumped isotope compositions of modern marine mollusk and brachiopod shells. Geochimica et Cosmochimica Acta 106:307-325 |
| Hertlein & Grant, 1944 | Hertlein, L. G., and U. S. Grant IV. 1944. The Cenozoic Brachiopoda of western North America. Publications of the University of California at Los Angeles in Mathematical and Physical Sciences 3:236 p., 21 |
| Hiller, 1986 | Hiller, N., 1986. The South African Museum’s Meiring Naude cruises, Part 16. Brachiopoda from the 1975–1979 cruises. Annals of the South African Museum 97:97–140. |
| Hiller, 1990 | Hiller, N., and D. I. MacKinnon. 2000. A reappraisal of the systematics of the *Stethothyris* group of brachiopods from the Cenozoic of New Zealand and Australia. New Zealand Journal of Geology and Geophysics 43:59–81. |
| Hiller, 1991 | Hiller, N. 1991. The southern African Recent brachiopod fauna. In D. I. MacKinnon, D. E. Lee, & J. D. Campbell, eds., Brachiopods through Time. Proceedings of the 2nd International Brachiopod Congress, University of Otago, Dunedin, New Zealand, 5–9 February, 1990. Balkema. Rotterdam. p. 439–445. |
| Hiller, 1994 | Hiller, N. 1994 The environment, biogeography, and origin of the Southern African recent brachiopod fauna. Journal of Paleontology 68: 776-786. |
| Hoffmann & Lüter, 2009 | Hoffmann, J. and Lüter, C. 2009. Shell development, growth and sexual dimorphism in the Recent thecideide brachiopod *Thecidellina meyeri* sp. nov. from the Lesser Antilles, Caribbean. Journal of the Marine Biological Association of the United Kingdom 89:469-479 |
| Hoffmann & Lüter, 2010 | Hoffmann, J. & Lüter, C. 2010. Shell development in thecidellinine brachiopods with description of a new Recent genus. *Special Papers in* *Palaeontology,* 84, 137-160. |
| Hoffmann et al., 2009 | Hoffmann et al. 2009. Recent thecideide brachiopods from the northern Great Barrier Reef, Australia (SW Pacific Ocean) |
| Holte, 2014 | Holte, B. (2014). MAREANO - Base-line mapping of fauna obtained with grab. Institute of Marine Research, Norway https://doi.org/10.15468/dlaxsw |
| IFREMER, 2016 | Institut Français de Recherche pour l'Exploitation de la Mer – IFREMER (2016): COMARGIS: Information System on Continental Margin Ecosystems https://doi.org/10.15468/0djslr |
| IndOBIS | Indian Ocean Biogeographic Information System (IndOBIS)- Distribution records of marine organisms from the Indian Ocean |
| Ishida, 2016 | Ishida S (2016). Marine Invertebrata specimen database of Osaka Museum of Natutal History. National Institute of Genetics, ROIS. Occurrence dataset https://doi.org/10.15468/zhubgk accessed via GBIF.org on yyyy-mm-dd. |
| Jackson & Stiasny, 1937 | Jackson, J. W., & G. Stiasny. 1937. The Brachiopoda of the Siboga Expedition. In M. C. W. Weber, ed., Siboga Expedité (Leiden) 27:20 |
| Jackson et al., 1971 | Jackson, J. B. C., T. F. Goreau, & W. D. Hartman. 1971. Recent brachiopod-coralline sponge communities and their paleoecological significance. Science 173:623–625. |
| Jackson, 1918 | Jackson, J. W. 1918. Brachiopoda. British Antactic (“Terra Nova”) Expedition 1910. British Museum of Natural History, Reports in Zoology 2(8):177–202. |
| Jackson, 1952 | Jackson, J. W. 1952. A revision of some South African Brachiopoda; with descriptions of new species. Annals of the South African Museum 41(1):40 |
| Jeffreys, 1869 | Jeffreys, J. G. 1869. The deep-sea dredging expedition in H.M.S. “Porcupine”. *Nature.* 1: 135-136. |
| Jeffreys, 1878 | Jeffreys, J. G. 1878. On the Mollusca procured during the "Lightning" and "Porcupine" Expeditions, 1868–70. (Part I), Volume for 1878. Proceedings of the Zoological Society of London. p. 393–416. |
| Jintsu-Uchifune & Yamamoto, 2016 | Jintsu-Uchifune, Y., Yamamoto, H. (2016) Marine organism occurrence data of the Asia-Pacific region extracted from literature. Available at http://www.godac.jamstec.go.jp/bismal/e/S9-5_Asia-Pacific. Accessed on yyyy-mm-dd. |
| JODC (2013) | JODC (2013) JODC Dataset. (Available: http://www.godac.jamstec.go.jp/bismal/j/JODC_J-DOSS. Accessed: YYYY-MM-DD). |
| Kaesler, 1997 | Kaesler, R. L., ed. 1997. Treatise on Invertebrate Paleontology. Part H (Revised), vol. 1. Geological Society of America & University of Kansas Press. Boulder, Colorado & Lawrence, Kansas. xx + 539 p., 417 |
| Kaesler, 2002 | Kaesler, R. L., ed. 2002. Treatise on Invertebrate Paleontology. Part H (Revised), vol. 4. Geological Society of America & Paleontological Institute. Boulder, Colorado & Lawrence, Kansas. xxxix + 768 p., 484 |
| Kenchington & Hammond, 1978 | Kenchington R. and Hammond L.S. 1978. Population structure, growth and distribution of Lingula anatina (Brachiopoda) in Queensland, Australia. Journal of Zoology 184(1):63-81. |
| Kowalewski & others, 2002 | Kowalewski, Michel, M. G. Simões, Monica Carroll, & D. L. Rodland. 2002. Abundant brachiopods on a tropical upwelling-influenced shelf (Southeast Brazilian Bight, South Atlantic). PALAIOS 17:277–286. |
| La Perna, 1998 | La Perna, R. 1998. A new Mediterranean Skenoides (Gastropoda: Skeneidae) from a shallow-water cave. Journal of Conchology 36(4):21–27. |
| Laperousaz, 2017 | Laperousaz T (2017): South Australian Museum Marine Invertebrates Collection. v1.1. CSIRO Oceans and Atmosphere. Dataset/Occurrence. http://ogc-act.csiro.au/ipt/resource?r=sam_marine_inverts&v=1.1 |
| Laurin & Gaspard, 1987 | Laurin, B., and Gaspard, D. 1987. Variations morphologiques et croissance du brachiopode abyssal *Macandrevia africana* Cooper. Oceanologica Acta 10:445–454. |
| Laurin, 1997 | Laurin, B. 1997. Brachiopodes récoltés dans les eaux de la Nouvelle-Calédonie et des îles Loyauté, Matthew et Chesterfield. In A. Crosnier, ed., Résultats des Campagnes MUSORSTOM, vol. 18. Mémoires du Muséum national d’Histoire naturelle 176:411–471. |
| Lee & Wilson, 1979 | Lee, D. E., & J. B. Wilson. 1979. Cenozoic and Recent rhynchonellide brachiopods of New Zealand: Systematics and variation in the genus *Notosaria*. Journal of the Royal Society of New Zealand 9(4):437–463. |
| Lee & Robinson, 2003 | Lee D. E. and Robinson J. H. 2003. *Kakanuiella* (gen. nov.) and *Thecidellina*: Cenozoic and Recent thecideide brachiopods from New Zealand. Journal of the Royal Society of New Zealand 33: 341-361. |
| Lee, 1978 | Lee, D. E. 1978. Aspects of the ecology and paleoecology of the brachiopod *Notosaria nigricans* (Sowerby). Journal of the Royal Society of New Zealand 8(4):395–417. |
| Lee, 1991 | Lee, D. E. 1991. Aspects of the ecology and distribution of the living Brachiopoda of New Zealand. In D. I. MacKinnon, D. E. Lee, & J. D. Campbell, eds., Brachiopods through Time. Proceedings of the 2nd International Brachiopod Congress, University of Otago, Dunedin, New Zealand, 5–9 February, 1990. Balkema. Rotterdam. p. 273–279, 1 fig. |
| Logan & Bitner, 2013 | Logan, A. and Bitner, M. A. 2013. New records of Recent Brachiopoda from the Red Sea with a description of a new species. Zootaxa 3746:161-74. |
| Logan & Long, 2001 | Logan, A., and Long, S.L., 2001. Shell morphology and geographical distribution of *Neocrania* (Brachiopoda, Recent) in the eastern North Atlantic and Mediterranean Sea. p. 71-79. In: Brachiopods Past and Present. Brunton, C.H.C., Cocks, L.R.M., Long, S.L. (Eds). Taylor & Francis, London |
| Logan & Noble, 1971 | Logan, Alan, & J. P. A. Noble. 1971. A Recent shallow water brachiopod community from the Bay of Fundy. Maritime Sediments 7(2):85–91. |
| Logan & Zibrowius, 1994 | Logan, A., and Zibrowius, H. 1994. A new genus and species of rhynchonellid (Brachiopoda, Recent) from submarine caves in the Mediterranean Sea. P.S.Z.N.: Marine Ecology 15 (1):77–88. |
| Logan et al., 1997 | Logan, A. MacKinnon, D. I. and Phorson. J. E. 1997. Morphology, distribution, life habits and phylogenetic affinities of the Recent brachiopod *Gwynia* *capsula* (Jeffreys). P.S.Z.N.: Marine Ecology 18:239–252. |
| Logan et al., 2002 | Logan, Alan, C. K. Bianchi, Carla Morri, Helmut Zibrowius, & Ghazi Bitar. 2002. New records of Recent brachiopods from the eastern Mediterranean Sea. Annali del Museo Civico di Storia Naturale “G. Doria,” Genova 94:407–418. |
| Logan et al., 2004 | Logan, Alan, C. K. Bianchi, Carla Morri, & Helmut Zibrowius. 2004. The present-day Mediterranean brachiopod fauna: Diversity, life habits, biogeography and paleobiogeography. In J. D. Ros, T. T. Packard, J. M. Gili, J. L. Pretus, & D. Blasco, eds., Biological Oceanography at the turn of the Millennium. Scientia Marina 68(supplement 1):163–170. |
| Logan et al., 2007 | 2007. Logan et al. New record of *Novocrania* (Brachiopoda, Craniida) from Madeira, with notes on Recent brachiopod occurrences in the Macaronesian archipelagos. Arquipélago. Life and Marine Sciences 24: 17-22. |
| Logan et al., 2015 | Logan, A. Hoffmann, J. Lüter C. 2015. Checklist of Recent thecideoid brachiopods from the Indian Ocean and Red Sea, with a description of a new species of Thecidellina from Europa Island and a re-description of T. blochmanni Dall from Christmas Island. Zootaxa 4013: 225-234. |
| Logan, 1975 | Logan, A. 1975. Ecological observations on the Recent articulate brachiopod Argyrotheca bermudana Dall from the Bermuda Platform. Bulletin of Marine Science 25:186–204. |
| Logan, 1977 | Logan, A. 1977. Reef-dwelling articulate brachiopods from Grand Cayman, B.W.I. Proceedings of the Third International Coral Reef Symposium, Miami, vol. 1. Biology. p. 87–93. |
| Logan, 1979 | Logan, A. 1979. The Recent Brachiopoda of the Mediterranean Sea. Bulletin de l’Institut Océanographique, Monaco 72(1,434):112 p., 22 |
| Logan, 1981 | Logan, A. 1981. Sessile invertebrate coelobite communities from shallow reef tunnels, Grand Cayman, B.W.I. Proceedings of the Fourth International Coral Reef Symposium, Manila 2:735–744. |
| Logan, 1983 | Logan, Alan. 1983. Brachiopoda collected by CANCAP I-III expeditions to the south-east North Atlantic, 1976–1978. Zoologische Mededelingen 57(18):165–189. |
| Logan & Noble, 1983 | Logan, A. & J. P. A. Noble. 1983. Recent brachiopods from Malta. Central Mediterranean Naturalist 1:34–42. |
| Logan, 1988a | Logan, A. 1988a. Brachiopoda collected by CANCAP IV and VI expeditions to the south-east North Atlantic. 1980–1982. Zoologische Mededelingen 62(5):59–74. |
| Logan, 1988b | Logan, A. 1988b. A new thecideid genus and species (Brachiopoda, Recent) from the southeast North Atlantic. Journal of Paleontology 62(4):546–551. |
| Logan, 1990 | Logan, A. 1990. Recent Brachiopoda from the Snellius and Luymes expeditions to the Surinam-Guyana Shelf, Bonaire-Curacao, and Saba Bank, Caribbean Sea, 1966 and 1969–72. Zoologische Mededelingen 63(11):123–136. |
| Logan, 1993 | Logan, A. 1993. Recent brachiopods from the Canarian-Cape Verdean region: Diversity, biogeographic affinities, bathymetric range and life habits. Courier Forschungs-Institut Senckenberg, 159:229–233. |
| Logan, 1998 | Logan, A. 1998. Recent Brachiopoda from the oceanographic expedition SEAMOUNT 2 to the north-eastern Atlantic in 1993. Zoosystema 20(4):549–562. |
| Logan, 2003 | Logan, A. 2003. Marine fauna of the Mljet National Park (Adriatic Sea, Croatia). 3. Brachiopoda. Natura Croatica 12(4):233–243.. |
| Logan, 2004 | Logan, A. 2004. Ecological, reproductive and ontogenetic features in Pajaudina atlantica Logan (Thecideidae, Brachiopoda, Recent) from the Canary Islands. P.S.Z.N.: Marine Ecology 25(3):207–215. |
| Logan, 2005 | Logan, A. 2005. A new lacazelline species (Brachiopoda, Recent) from the Maldive Islands, Indian Ocean. Systematics and Biodiversity 3(1):97–104. |
| Logan, 2007 | Logan, A. 2007. Geographic distribution of extant articulated brachiopods. *In: Treatise on Invertebrate Paleontology, Part H, Brachiopoda (revised), vol.6, 3082–3115. Geological Society of America, Boulder, Colorado, and University of Kansas Press, Lawrence, Kansas.* |
| Lüter & Cohen, 2002 | Lüter, C. and Cohen. B. L. 2002. DNA sequence evidence for speciation, paraphyly and a Mesozoic dispersal of cancellothyrid articulate brachiopods. Marine Biology 141:65–74. |
| Lüter et al., 2003 | Lüter, C., Worheide, G., and Reitner, J., A new thecideid genus and species (Brachiopoda, Recent) from submarine caves of Osprey Reef (Queensland Plateau, Coral Sea, Australia). Journal of Natural History, 37:1423-1432. |
| Lüter, 2004 | Lüter, C. 2004. How brachiopods get covered with nanometric silicon chips. Proceedings of the Royal Society London (series B, Supplement) Biology Letters 271:S465–S467. |
| Lüter, 2005 | Lüter, C. 2003. The first recent species of the unusual brachiopod *Kakanuiella* (Thecideidae) from New Zealand deep waters. *Systematics and Biodiversity.* 3(1): 105-111. |
| Lüter, 2008 | Lüter, C. 2008. Recent brachiopods collected during the deep-sea cruise SO 168 ZEALANDIA with the research vessel FS Sonne between Mt. Spong (Tasman Sea) and the Chatham Islands (Pacific) in 2002–2003. Fossils and Strata 54:311-320. |
| MacKinnon & Long, 2000 | MacKinnon, D. L., & S. L. Long. 2000. *Terebratula californiana* Küster, 1844, and reappraisal of west coast North American brachiopod species referred to the genus *Laqueus* Dall, 1870. Bulletin of the Natural History Museum of London (Geology) 56(2):85–90. |
| MacKinnon & Long, 2009 | MacKinnon, D. I., and Long S. L., 2009. A New Species of the Recent Brachiopod Genus *Shimodaia* (Laqueoidea: Terebratulida) from the South China Sea. Paleontological Research, 13:309-317. |
| MacKinnon & Hiller, 2010 | MacKinnon, D. & Hiller, N. 2010. Endoskeletal plate development in the Recent Indo-Pacific brachiopod genus *Jolonica* Dall, 1920 (Terebratulida: Laqueoidea). Special Papers in Palaeontology, 84, 193–202. |
| MacKinnon et al., 1997 | MacKinnon, D. I., Saito, M., Endo, K., 1997. Morphology and systematics of the Recent Japanese brachiopod *Shimodaia pterygiota* gen. et sp. nov. : Laqueidae : Terebratulida. Paleontological Research 1:225-233. |
| Maclellan & Sprague, 2016 | Maclellan, D.C. and J.B. Sprague. 2016. FRB: Bottom fauna of Saint John Harbour and estuary as surveyed in 1959 and 1961. Version 1 In OBIS Canada Digital Collections. Bedford Institute of Oceanography, Dartmouth, NS, Canada. Published by OBIS, Digital http://www.iobis.org/. Accessed on –INSERT DATE |
| Manceñido & Griffin, 1988 | Manceñido, M. O., and Miguel Griffin. 1988. Distribution and palaeoenvironmental significance of the genus *Bouchardia* (Brachiopoda, Terebratellidina): Its bearing on the Cenozoic evolution of the South Atlantic. Revista Brasileira de Geociências 18(2):201–211. |
| Marine Benthic Fauna List | The Danish Biodiversity Information Facility, Marine Benthic Fauna List, Island of Læsø, Denmark. https://doi.org/10.15468/ty0smg |
| Martinez et al. 2005 | Lophophorata: Phoronida, Brachiopoda [BOOK] |
| Massy, 1925 | Massy, A. L. 1925. The Brachiopoda of the coasts of Ireland. Proceedings of the Royal Irish Academy (section B) 37:37–46. |
| Mauzey et al., 1968 | Mauzey, K. P. Birkeland, C. Dayton, P. K. 1968. Feeding Behavior of Asteroids and Escape Responses of their Prey in the Puget Sound Region. Ecology 49: 603-619 |
| McCammon & Buchsbaum, 1968 | McCammon, H. M., and Ralph Buchsbaum. 1968. Size and shape variation of three Recent brachiopods from the Strait of Magellan. In W. L. Schmitt and G. A. Llano, eds., Biology of the Antarctic seas, Antarctic Research Series 11(3):215–225. |
| McCammon, 1969 | McCammon, H. M. 1969. The Food of Articulate Brachiopods. Journal of Paleontology 43:976-985. |
| McCammon, 1973 | McCammon, H. M. 1973. The ecology of *Magellania venosa*, an articulate brachiopod. Journal of Paleontology 47:266–278. |
| Meidlinger et al., 1998 | Meidlinger, K. Tyler, P. A. Peck, L. S. 1998. Reproductive patterns in the Antarctic brachiopod *Liothyrella uva*. Marine Biology 132: 153–162 |
| Meile & Pajaud, 1971 | Meile, B., & Pajaud, D. 1971. Présence de Brachiopodes dans le Grand Banc des Bahamas. Comptes rendus des séances de l‘Académie des Sciences 273:469–472. |
| Miller et al., 2014 | Miller R, Nozères C, Kennedy M (2014). DFO Quebec Region MLI museum collection. Version 2. OBIS Canada Digital Collections. Published by OBIS http://www.iobis.org/. Accessed on –INSERT DATE |
| Motchurova-Dekova et al., 2002 | Motchurova-Dekova N. Saito, M. Endo, K. 2002. The Recent rhynchonellide brachiopod *Parasphenarina cavernicola* gen. et sp. nov. from the submarine caves of Okinawa, Japan. Paleontological Research 6:299–319. |
| Muir-Wood, 1959 | Muir-Wood, H. M. 1959. Report on the Brachiopoda of the John Murray Expedition. In The John Murray Expedition 1933–34, Scientific Reports 10:283–317. |
| Muir-Wood, 1960 | Muir-Wood, H. M. 1960. Homeomorphy in Recent Brachiopoda: Abyssothyris and Neorhynchia. The Annals and Magazine of Natural History (series 13) 3:521–528. |
| Museums Victoria Marine Invertebrates Collection | Museums Victoria Marine Invertebrates Collection The publisher and rights holder of this work is CSIRO Oceans and Atmosphere. This work is licensed under a Creative Commons Attribution (CC-BY) 4.0 License. |
| NaGISA Project | NaGISA Project |
| NatureWatch NZ, 2016 | NatureWatch NZ (2016). Marine species citizen-science observations from NatureWatch NZ. Southwestern Pacific OBIS, National Institute of Water and Atmospheric Research (NIWA), Wellington, New Zealand, 13030 records, Online http://nzobisipt.niwa.co.nz/resource.do?r=naturewatchnz released on March 26, 2017. |
| Nauendorf et al., 2014 | Nauendorf, A.Wöhrheide, G.and Lüter, C. 2014. Revision of the brachiopod genus *Amphithyris* (Rhynchonelliformea Platidiidae) with descriptions of two new species. Zootaxa, 3847:221-240. |
| Neall, 1970 | Neall, V. E. 1970. Notes on the ecology and paleoecology of *Neothyris*, an endemic New Zealand brachiopod. New Zealand Journal of Marine and Freshwater Research 4(2):117–125. |
| Neall, 1972 | Neall, V. E. 1972. Systematics of the endemic New Zealand brachiopod *Neothyris*. Journal of the Royal Society of New Zealand 2(2):229–247. |
| NEFSC Benthic Database | Northeast Fisheries Science Center, National Marine Fisheries Service, NOAA, U.S. Department of Commerce. 2010. NEFSC Benthic Database. Northeast Fisheries Science Center, 166 Water Street, Woods Hole Laboratories, Woods Hole, MA 02543.Retrieved from http://www.usgs.gov/obis-usa/ |
| NIWA (2015) | NIWA (2015). Antarctic Biodiversity Studies 2006 - Ross Sea, Scott Island, and Balleny Islands (TAN0602). Southwestern Pacific OBIS, National Institute of Water and Atmospheric Research, Wellington, New Zealand, 1061 records, Online http://nzobisipt.niwa.co.nz/resource.do?r=tan0602 released on April 17, 2015. |
| NIWA (2018) | NIWA (2018): NIWA Invertebrate Collection. v1.1. The National Institute of Water and Atmospheric Research (NIWA). Dataset/Occurrence. https://nzobisipt.niwa.co.nz/resource?r=obisspecify&amp;v=1.1 |
| NIWA (2014) | NIWA (2014). 2004 Ross Sea Biodiversity Survey (BIOROSS) data. Southwestern Pacific OBIS, National Institute of Water and Atmospheric Research, Wellington, New Zealand, 4093 records, Online http://nzobisipt.niwa.co.nz/resource.do?r=mbis_caml released on June 18, 2014. |
| NOAA (2018) | NOAA (2018): National Benthic Inventory. v1. GBIF Secretariat. Dataset/Occurrence. https://nccos-coastalscience-products-web-ipt.azurewebsites.net/ |
| Noble et al., 1976 | Noble, J. P. A. Logan, A. Webb, G. R. 1976. The Recent *Terebratulina* Community in the rocky subtidal zone of the Bay of Fundy, Canada. Lethaia 9: 1-18. |
| Norwegian Oil Industry Association, 2002 | The Norwegian Oil Industry Association, 2002: Offshore reference stations, Norwegian/Barents Sea. The Norwegian Oil Industry Association (OLF), Akvaplan-niva and Det Norske Veritas, Norway |
| Ocean Survey (2013) | Ocean Survey 20/20 (2013). International Polar Year and Census of Antarctic Marine Life Ross Sea voyage (TAN0802) biodiversity data. Southwestern Pacific OBIS, National Institute of Water and Atmospheric Research, Wellington, New Zealand, 8748 records, Online http://nzobisipt.elasticbeanstalk.com/resource.do?r=mbis_caml released on Dec 12, 2013. |
| Ostrow et al., 2001 | Ostrow, D. G., S. R. Wing, P. V. Mladenov, & M. S. Roy. 2001. Genetic differentiation of *Terebratella sanguinea* in the New Zealand fiords: A dispersal barrier in the marine environment? In C. H. C. Brunton, L. R. M. Cocks, & S. L. Long, eds., Brachiopods Past and Present. The Systematics Association Special Volume Series 63:150–159. |
| Pajaud, 1970 | Pajaud, Daniel. 1970. Monographie des Thécidées. Mémoires de la Société Géologique de France (new series) 49(112):349 p., 140 |
| Pajaud, 1974 | Pajaud, D. 1974. Écologie de Thécidées. Lethaia 7:203–218. |
| Parkinson et al., 2005 | Parkinson, D., Curry, G. B., Cusack, M., Fallick, A. E. 2005. Shell structure, patterns and trends of oxygen and carbon stable isotopes in modern brachiopod shells. Chemical Geology 219: 193-235. |
| Parr, (MarLIN) | Parr, J. Marine Life Information Network (MarLIN) marine survey data (Professional). Marlin, Collated Marine Life Survey Datasets, Marine Biological Association of the UK, Plymouth, UK |
| Peck & Harper, 2010 | Peck L. S. and Harper, E. M. 2010. Variation in size of living articulated brachiopods with latitude and depth. Marine Biology 157: 2205–2213. |
| Peck et al., 1987 | Peck, L. S. Clarke, A. Holmes, L. J. 1987. Summer metabolism and seasonal changes in biochemical composition of the Antarctic brachiopod Liothyrella uva (Broderip, 1833). Journal of Experimental Marine Biology and Ecology 114: 85-97. |
| Peck et al., 1997 | Peck, L. S., Brockington, S. and Brey, T. A. 1997. Growth and metabolism in the Antarctic brachiopod *Liothyrella uva*. Philosophical Transactions of the Royal Society of London (series B, Biological Sciences) 352:851–858. |
| Pohle et al., 2005 | Pohle, Gerhard, A. Holmes. (2005). Grand Manan Basin Benthos. Version 1. In OBIS Canada Digital Collections. Bedford Institute of Oceanography, Dartmouth, NS, Canada. Published by OBIS, Digital http://www.iobis.org/. Accessed on –INSERT DATE |
| Puura & Nemliher, 2001 | Puura, I., and Nemliher, J. 2001. Apatite varieties in Re-cent and fossil linguloid brachiopod shells. In Brunton, C. H. C.; Cocks, L. R. M.; and Long, S. L., eds. Brachiopods past and present. London, Taylor & Francis,441 p. |
| Pyle, 2016 | Pyle R (2016). Bernice P. Bishop Museum. Version 8.1. Bernice Pauahi Bishop Museum. Occurrence dataset https://doi.org/10.15468/s6ctus accessed via GBIF.org on 2018-11-16. |
| Queen Victoria Museum and Art Gallery | Queen Victoria Museum and Art Gallery - Marine Invertebrates |
| Queensland Museum | Queensland Museum Other Invertebrates - marine records |
| Ralph & Yaldwin, 1956 | Ralph P.M. and Yaldwin, J.C. 1956. Seafloor Animals from the Region of Portobello Marine Biological Station, Otago Harbour. Tuatara: Volume 6, Issue 2, December 1956 |
| REVIZEE | Invertebrates, REVIZEE South Region - Benthos |
| Rhodes & Thayer, 1990 | Rhodes M. C. and Thayer, C. W. 1990. effects of turbidity on suspension feeding are brachiopods better than bivalves? In: Brachiopods through time, MacKinnon D. I., Lee D. E. & J. D. Campbell Eds, Balkema, Rotterdam, pp. 191-196. |
| Richardson, 1973 | Richardson, J. R. 1973. Studies on Australian Cainozoic Brachiopods 2. The family Laqueidae (Terebratellidae). Proceedings of the Royal Society of Victoria 85:117–126. |
| Richardson, 1981 | Richardson, J. R. 1981. Recent brachiopods from New Zealand — background to the study cruises of 1977–79. New Zealand Journal of Zoology 8:133–143. |
| Richardson, 1987 | Richardson, J. R. 1987. Brachiopods from carbonate sands of the Australian Shelf. Proceedings of the Royal Society of Victoria 99(1):37–50. |
| Richardson, 1994 | Richardson, J. R. 1994. Origins and dispersal of a brachiopod family—The systematics, biogeography and evolution of the family Terebratellidae. Proceedings of the Royal Society of Victoria 106:17–29. |
| Richardson, 1997a | Richardson, J. R., 1997a, Ecology of articulated brachiopods, in: Brachiopoda (A. Williams, ed.), Treatise on Invertebrate Paleontology, Part H, revised, GSA, Boulder, and U. Kansas, Lawrence, pp. 441–462. |
| Richardson, 1997b | Richardson J. R., 1997b. Biogeography of articulated brachiopods. In: Kaesler R ed. Boulder, Colorado, Geological Society of America and University of Kansas. Treatise of invertebrate paleontology, Brachiopoda (rev.), Part H. Vol. 1, 463-472. |
| Rioult, 1971 | Rioult, M. 1971. *Argyrotheca cistellula* (Searles-Wood) (Brachiopode, Terebratellacea) au large des côtes du Calvados. Bulletin de la Société Linnéenne de Normandie 102:26–28. |
| Robinson & Lee, 2011 | Robinson, J. H. and Lee, D. E. 2011. Spine formation in *Novocrania* and *Danocrania* (Brachiopoda, Craniata). Memoirs of the Association of Australasian Palaeontologists 41: 25–37. |
| Robinson et al., 2016 | Robinson, J. H. Donald, K. M. Brandt, A. J. and Lee, D. E. 2016. *Magasella sanguinea* (Leach, 1814) and *Magasella haurakiensis* (Allan, 1931): resolving the taxonomic placement of these endemic New Zealand brachiopods using morphological and molecular traits. Journal of the Royal Society of New Zealand 46: 139-163. |
| Robinson, 2017 | Robinson, J. H. 2017. A review of all Recent species in the genus *Novocrania* (Craniata, Brachiopoda). Zootaxa 4329: 501-559. |
| Rojas et al., 2015 | Rojas et al. 2015. Brachiopods from off the San Bernardo Archipelago (Colombian Caribbean), with comments on specific synonymies in *Tichosina* Cooper, 1977 |
| Rudwick, 1962b | Rudwick, M. J. S. 1962b. Notes on the ecology of brachiopods in New Zealand. Transactions of the Royal Society of New Zealand, Zoology 1(25):327–335. |
| Taddei Ruggiero, 2001 | Taddei Ruggiero, E. 2001. Brachiopods of the Isca submarine cave: observations during ten years. In: Brachiopods, Past and Present (Eds C. Howard, CHC Brunton, L. Robin, M.Cocks and SL Long), pp. 261-267. Taylor and Francis, New York, NY. |
| Saito & Tazawa, 2002 | Saito, M., and Tazawa, J. 2002. *Hemithiris woodwardi* (A. Adams) (Rhynchonellida, Brachiopoda) from the Pleistocene Shichiba Formation, Sado Island, central Japan. Science Reports of Niigata University (series E, Geology) 17:7–15. |
| Saito, 1996 | Saito, M. 1996. Early loop ontogeny of some Recent laqueid brachiopods. Tran. Proc. Palaeont. Soc. Japan, N.S., 183: 485-499. |
| Saiz Salinas, 1989 | Saiz Salinas, J. I. 1989. Verzeichnis der rezenten Brachiopoden (Brachiopoda) von den iberischen Küsten und den angrenzenden Meeren. Bonner Zoologische Beiträge 40(2):141–154. |
| Santagata & Tunnell, 2009 | Santagata, S. and J. W. Tunnell Jr. 2009. Brachiopoda of the Gulf of Mexico, Pp. 1137-1141.Felder, D.L. and D.K. Camp (eds.), Gulf of Mexico-Origins, Waters, and Biota. Biodiversity. Texas A&M Press, College Station, Texas. |
| Savage, 1972 | Savage, N. M. 1972. Some observations on the behaviour of the Recent brachiopod *Megerlina pisum* under laboratory conditions. Lethaia 5:61–67.. |
| SCAR - AntOBIS (2019) | SCAR - AntOBIS (2019). Biogeographic distribution of the Antarctic and Sub-Antarctic brachiopods (living forms). |
| Seidel & Lüter, 2014 | Seidel, R. and Lüter, C. 2014. Overcoming the fragility – X-ray computed micro-tomography elucidates brachiopod endoskeletons. Frontiers in Zoology 11:65 |
| Simões et al., 2004 | Simões, M. G. Kowalewski, M. Mello, L. H. C. Rodland, D. L. & Carroll, M. 2004. Recent brachiopods from the southern Brazilian shelf_ palaeontological and biogeographical implications. Palaeontology 47: 515-533. |
| Simon & Hoffmann, 2013 | Simon, E. and Hoffmann, J. 2013. Discovery of Recent thecideide brachiopods (Order Thecideida, Family Thecideidae) in Sulawesi, Indonesian Archipelago, with implications for reproduction and shell size in the genus *Ospreyella*. Zootaxa 3694: 401–433. |
| Simon & Willems, 1999 | Simon, E. and Willems, G. 1999. *Gwynia capsula* (Jeffreys, 1859) and other Recent brachiopods from submarine caves in Croatia. Bullétin de l’Institut Royal des Sciences naturelles de Belgique, Biologie 69:15–21. |
| Simon et al., 2016 | Simon, E., Logan, A., Zuschin, M., Mainguy, J., Mottequin, B. 2016. *Lenticellaria* and *Hillerella*, new kraussinoid genera (Kraussinoidea, Brachiopoda) from Indo-Pacific and Red Sea waters evolution in the subfamily Megerliinae. Zootaxa 4137: 1-34. |
| Simon et al., 2018 | Simon, E., Lüter, C., Logan, A., Mottequin, B. 2018. Recent thecideide brachiopods (Thecideida, Thecideoidea) from northern Sulawesi (Indonesia) with discovery of a new Thecidellina species (Thecidellinidae). Zootaxa 4526:481-515. |
| Simon et al., 2019 | Simon, E., Logan, A., Theuerkauff, D., Mottequin, B. 2019. Recent thecideide brachiopods from a submarine cave in the Department of Mayotte (France), northern Mozambique Channel. Zootaxa 4613: 201-239. |
| Simon, 2010 | Simon, E. 2010. Two micromorphic megathyrid brachiopods (Terebratulida, Megathyridoidea) from the Indonesian Archipelago. Bulletin de l'Institut royal des Sciences naturelles de Belgique, Biologie. 80:277-295. |
| Simonet Roda et al. 2019 | Simonet Roda, M., Ziegler, A., Griesshaber, E., Yin, X. Rupp, U., Greiner, M., Henkel, D., Häussermann, V., Eisenhauer, A., Laudien, J., Schmahl, W.W. 2019. Terebratulide brachiopod shell biomineralization by mantle epithelial cells. Journal of Structural Biology 207: 136-157. |
| Sloan et al., 2004 | Sloan, N.A., Bartier, P.M., Austin, W.C. 2004. Gwaii Haanas Invertebrates (Living marine legacy of Gwaii Haanas II: Marine invertebrate baseline to 2000). Parks Canada-Technical Reports in Ecosystem Science. OBIS Canada, Bedford Institute of Oceanography, Dartmouth, Nova Scotia, Canada, Version 1, Digital, retrieved from http://iobis.org/. |
| Smolyar (2009) | Baranova, O.K, T.D. O'Brien, T.P. Boyer and I.V. Smolyar (2009). Plankton data. Chapter 16 in Boyer, T. P., J. I. Antonov , O. K. Baranova, H. E. Garcia, D. R. Johnson, R. A. Locarnini, A. V. Mishonov, T. D. O'Brien, D. Seidov, I. V. Smolyar, M. M. Zweng, 2009. World Ocean Database 2009. S. Levitus, Ed., NOAA Atlas NESDIS 66, U.S. Gov. Printing Office, Wash., D.C., 216 pp., DVDs |
| Southern Surveyor Voyage, 2007 | CSIRO - Southern Surveyor Voyage SS 02/2007, biodiversity survey for SE Marine Protected Areas including the Tasmanian Sea Mounts Marine Reserve |
| Southern Surveyor voyage, 97 | CSIRO - Southern Surveyor voyage SS 01/97, Tasmanian Seamounts study |
| Southwestern Pacific OBIS, 2014 | Southwestern Pacific OBIS (2014). British Antarctic (Terra Nova) Expedition, 1910-1913. Southwestern Pacific OBIS, National Institute of Water and Atmospheric Research (NIWA), Wellington, New Zealand, 1779 records, Online http://nzobisipt.niwa.co.nz/resource.do?r=terranova released on July 29, 2014 |
| Stüder, 1889 | Stüder, T. 1889. Die Forschungsreise S.M.S. “Gazelle” in den Jahren 1874 bis 1876. III. Zoologie und Geologie. Mittler and Sohn. Berlin. 322 p., 33 |
| Suter, 1908b | Suter, H. (1908b) Result of dredging for Mollusca near Cuvier Island, with descriptions of new species. Transactions and Proceedings of the New Zealand Institute, 40, 344Â–359, pls. 26Â–27, 30. |
| SWPRON, 2018 | SWPRON, 2018. Data from: Scientific results of the New Zealand Government Trawling Expedition, 1907. Waite, E.R. (ed.). Records of the Canterbury Museum, Vol. 2, No. 2. |
| Tasmanian Museum a | Tasmanian Museum and Art Gallery Invertebrate Collection - marine records |
| Tasmanian Museum b | Tasmanian Museum and Art Gallery provider for OZCAM - marine records |
| Thayer & Allmon, 1991 | Thayer, C. W., and Allmon, R. A. 1991. Unpalatable thecideid brachiopods from Palau: Ecological and evolutionary implications. In D. I. MacKinnon, D. E. Lee, & J. D. Campbell, eds., Brachiopods through Time. Proceedings of the 2nd International Brachiopod Congress, University of Otago, Dunedin, New Zealand, 5–9 February, 1990. Balkema. Rotterdam. p. 253–260. |
| Thayer, 1975 | Thayer, C. W. 1975. Size-frequency and population structure of brachiopods. Palaeogeography, Palaeoclimatology, Palaeoecology 17:139–148. |
| Thayer, 1977 | Thayer, C. W. 1977. Recruitment, growth, and mortality of a living articulate brachiopod, with implications for the interpretation of survivorship curves. Paleobiology 3: 98-109. |
| Thomson, 1927 | Thomson, J. A. 1927. Brachiopod morphology and genera (Recent and Tertiary). New Zealand Board of Science and Art, Manual No. 7:333 p., 103 |
| Tommasi, 1970 | Tommasi, L. R. 1970. Sôbre o Braquiópode *Bouchardia rosea* (Mawe, 1823). Boletim do Instituto Oceanografico, São Paulo 19:33–42. |
| Tort, 2003 | Tort, A. 2003. Morphological plasticity of the outline and the internal structures of the shell of the Recent Terebratella tenuis sp. nov. (Brachiopoda, Terebratulida). Zoomorphology 122:47–54. |
| Tunnicliffe & Wilson, 1988 | Tunnicliffe, V. and Wilson, K. 1988. Brachiopod populations: Distribution in fjords of British Columbia (Canada) and tolerance of low oxygen concentrations. Marine Ecology—Progress Series 47:117–128. |
| Twelvetrees & Petterd, 1900 | Twelvetrees, W. H. and Petterd W. F. 1900. On the genus *Kraussina* in Tasmania. |
| University of Florida Museum of Natural History | University of Florida Museum of Natural History Invertebrate Zoology Collection |
| Van Guelpen, 2016 | Van Guelpen, L., 2016. Atlantic Reference Centre Museum of Canadian Atlantic Organisms - Invertebrates and Fishes Data. Version 4 In OBIS Canada Digital Collections. Bedford Institute of Oceanography, Dartmouth, NS, Canada. Published by OBIS, Digital http://www.iobis.org/. Accessed on –INSERT DATE |
| Webb et al., 1976 | Webb, G. R., Logan, A. & Noble, J. P. A. 1976. Occurrence and Significance of Brooded Larva in a Recent Brachiopod, Bay of Fundy, Canada. Journal of Paleontology 50: 869-871. |
| Wesenberg-Lund, 1938 | Wesenberg-Lund, E. 1938. Brachiopoda. In The Zoology of Iceland. Ejnar Munksgaard. Copenhagen and Reykjavik. 11 p., 3 |
| Wesenberg-Lund, 1939 | Wesenberg-Lund, E. 1939. Recent Norwegian Brachiopods. Det Kongelige NorskeVidenskabers Selskab, Forhandlinger 11(52):201–204, 1 |
| Wesenberg-Lund, 1940a | Wesenberg-Lund, E. 1940a. Brachiopoda. In A. S. Jensen, W. Lundbeck, Th. Mortensen, and R. Spèarck, eds., The Zoology of the Faroes 59:8 p., 2 |
| Wesenberg-Lund, 1940b | Wesenberg-Lund, E. 1940b. Brachiopods from the waters west of Greenland. The Godthaab Expedition 1928. Meddelelser om Grønland 80(6):24 p., 9 |
| Wesenberg-Lund, 1941 | Wesenberg-Lund, E. 1941. Brachiopoda. The Danish Ingolf-Expedition 4 (12):17 p., 3 fig., 10 charts. White, C. A. 1879. Paleontological Papers No.9; Fossils of the Jura-Trias of Southeastern Idaho. United States Geological Survey of the Territories, Bulletin 5(1):105–117. |
| Western Australian Museum | Western Australian Museum Marine Invertebrate Collection, http://ogc-act.csiro.au/ipt/resource?r=wam_marine_inverts |
| Witman & Cooper, 1983 | Witman, J. D. and Cooper, R. A. 1983. Disturbance and contrasting patterns of population structure in the brachiopod *Terebratulina septentrionalis* (Couthouy) from two subtidal habitats. Journal of Experimental Marine Biology and Ecology 73:57–79. |
| Yamamoto et al., 2011 | Yamamoto, K. Asami, R. Iryu, Y. 2011. Brachiopod taxa and shell portions reliably recording past ocean environments toward establishing a robust paleoceanographic proxy. Geophysical research letters 38: L13601. |
| Ye et al., 2018 | Ye, F. Crippa, G. Angiolini, L. Brand, U. Capitani, G. Cusack, M. Garbelli, C. Griesshaber, E. Harper, E. Schmahl, W. 2018. Mapping of recent brachiopod microstructure A tool for environmental studies. Journal of Structural Biology 201: 221-236. |
| Ye et al., 2019 | Ye, F., Jurikova, H., Angiolini, L., Brand, U., Crippa, G., Henkel, D., Laudien, J., Hiebenthal, C. & Smajgl, D. 2019. Variation in brachiopod microstructure and isotope geochemistry under low-pH–ocean acidification–conditions. Biogeosciences 16: 617-642. |
| Yellow Sea | Qualitative analysis of macrobenthos in the Yellow Sea, This dataset contains macrobenthos data collected in the Yellow Sea during the 15-28 June 2007. The samples were collected with Agassiz trawl. |
| Yellow Sea 2 | Quantitative study of macrobenthos in the Yellow Sea, This dataset contains macrobenthos data collected in the Yellow Sea during the 15-28 June 2007. |
| Zezina, 2014 | Zezina O.N. 2014. Deep-sea fauna of European seas: An annotated species check-list of benthic invertebrates living deeper than 2000 m in the seas bordering Europe. Brachiopoda // Invertebrate Zoology. Vol.11. No.1: 83-88 |
| Zezina, 1965 | Zezina O. N., 1965. Distribution of the deepwater brachiopod *Pelagodiscus atlanticus* (King) [in Russian]. Okeanology, 5 (2), 354-358. |
| Zezina, 1970 | Zezina, O. N. 1970. Brachiopod distribution in the Recent ocean, with reference to problems of zoogeographic zoning. Paleontologicheskii Zhurnal 2:3–17. In Russian. |
| Zezina, 1975a | Zezina, O. N. 1975a. Recent Caribbean deep-sea brachiopod fauna, the sources and the conditions of its formation. Akademiia Nauk CCCP, Moscow 188–195, 3 fig. In Russian with English abstract. |
| Zezina, 1976a | Zezina, O. N. 1976a. The Ecology and Distribution of Modern Brachiopods (Nauka, Moscow, 1976a) [in Russian]. |
| Zezina, 1976b | Zezina, O. N. 1976b. [A new genus of Recent terebratelloid brachiopod from the sublittoral of the Kurilo- Kamchatka region.] In Donnaia Fauna Kraevykh Morei SSSR [Bottom Fauna of the USSR Fringing Seas]. Akademia Nauk SSSR, P. P. Shirshov Institute of Oceanology. Moscsow. p. 101–105, 1 pl. In Russian with English abstract. |
| Zezina, 1980 | Zezina, O. N. 1980. On composition, distribution and some biological features of the cold water brachiopods in the Southern Hemisphere [in Russian]. In: Ecological investigations of the shelf, Shirshov Inst. Oceanol. Moscow, p. 9-35 |
| Zezina, 1981a | Zezina, O. N. 1981a. Recent deep-sea Brachiopoda from the western Pacific. Galathea Report 15:7–20. |
| Zezina, 1981b | Zezina, O. N. 1981b. The composition and the ways of formation of the thalassobathyal brachiopod fauna. In A. P. Zuznetsov & N. A. Mironov, eds., Benthos of the submarine mountains Marcus-Necker and adjacent Pacific regions. Akademiia Nauk CCCP. Moscow. p. 141–149, 1 pl. In Russian with English abstract. |
| Zezina, 1981c | Zezina, O. N. 1981c. New and rare cancellothyroid brachiopods in the bathyal and abyssal ocean. In Deep-water benthic fauna of the Pacific Ocean. Trudy Instituta Okeanologie, Akademiia Nauk CCCP, Moscow 115:155–164. In Russian. |
| Zezina, 1985 | Zezina, O. N. 1985. Modern Brachiopods and Problems of the Bathyal Zones of the Ocean (Nauka, Moscow, 1985) [in Russian]. |
| Zezina, 1987 | Zezina, O. N. 1987. Brachiopods collected by BENTHEDI-Cruise in the Mozambique Channel. Bulletin du Muséum national d’Histoire naturelle (series 4) 9(section A, no. 3):551–563. |
| Zezina, 1990a | Zezina, O. N. 1990a. Composition and distribution of articulate brachiopods from the underwater rises of the eastern Pacific. Akademiia Nauk CCCP, Moscow 124:264–268, 1 fig. In Russian with English abstract. |
| Zezina, 1990b | Zezina, O. N. 1990b. Brachiopods of Northern Siberia (in Russian). Issled. Faun. Mor., Leningrad, 37 (45), 139-146. |
| Zezina, 1991 | Zezina, O. N. 1991. New species of cancellothyroid brachiopods from the “Atlantis” cruise to the Mid-Atlantic Ridge. Zoologicheski Zhurnal 70(6):152–153. In Russian. |
| Zezina, 1994 | Zezina, O. N. 1994. Deep-sea brachiopods. Their peculiarities in morphology and evolution. Sarsia 79: 59-64. |
| Zezina, 1997a | Zezina, O. N. 1997a. Annotated list of recent brachiopods species in the seas of Russia and adjacent waters [in Russian]. In: Composition and distribution of bottom invertebrate animals in the seas of Russia and adjacent waters [in Russian]. Russian Acad. Sci., Moscow, p. 61-73. |
| Zezina, 1997b | Zezina, O. N. 1997b. Biogeography of the Bathyal Zone. Advances in Marine Biology 32:389–426. |
| Zezina, 1997c | Zezina, O. N. 1997c. Recent brachiopods in the composition of the biofiltering biota of Russian Seas. Akademiia Nauk. Moscow. 83 p, 46 fig. In Russian. |
| Zezina, 1997d | Zezina, O. N. 1997d. Brachiopods in a natural biofilter at the the shelves and slopes in the far-eastern seas of Russia [in Russian]. In: Composition and distribution of bottom invertebrate animals in the seas of Russia and adjacent waters [in Russian]. Russian Acad. Sci., Moscow, p. 52-60. |
| Zezina, 2000 | Zezina, O. N. 2000. Russian collections of the deep-sea brachiopods in the Atlantic Ocean. In A. P. Kuznetsov and O. N. Zezina, eds., Benthos of the Russian seas and the northern Atlantic. Akademiia Nauk. Moscow. 26–36. In Russian with English abstract. |
| Zezina, 2001a | Zezina, O. N. 2001a. Global surface-water circulation and the main features of brachiopod biogeography. In C. H. C. Brunton, L. R. M. Cocks, & S. L. Long, eds., Brachiopods Past and Present. The Systematics Association Special Volume Series 63:102–107. |
| Zezina, 2001b | Zezina, O. N. 2001b. Articulate brachiopods near Vietnam shores in the belt of suspension-feeders at the continental slope of Asia. In A. P. Kuznetsov and O. N. Zezina, eds., Composition and structure of the marine bottom biota. Akademiia Nauk. Moscow. p. 63–68, 2 fig. In Russian with English abstract. |
| Zezina, 2005 | Zezina, O. N. 2005. On the systematic position of some Recent brachiopod species from the Norfolk Ridge (West Pacific). Invertebrate Zoology 2:29–33. |
| Zezina, 2006 | Zezina, O. N. 2006. Deep-Sea Brachiopods in Russian Collections from the Atlantic Ocean. in Biogeography of the North Atlantic Seamounts, Ed. by A. N. Mironov, A. V. Gebruk, and A. J. Southward (KMK Scientific Press Ltd., Moscow, 2006), pp. 67–75. |
| Zezina, 2008 | Zezina, O. N. 2008. Biogeography of the recent brachiopods. Paleontological Journal 42: 830-858. |
| Zezina, 2009 | Zezina, O. N. 2009. Relicts of the Tethyan Fauna in the Recent Oceans. Paleontological Journal 43:877-880. |
| Zezina, 2010 | Zezina, O. N. 2010. Check-list of Holocene Brachiopods Annotated with Geographical Ranges of Species. Paleontological Journal 44:1176–1199. |
