# Supplementary material for: Global biogeography of living brachiopods: Bioregionalization patterns and possible controls
Source: PLoS One. 2021 Nov 8;16(11):e0259004. doi: 10.1371/journal.pone.0259004 (PMC8575269; doi:10.1371/journal.pone.0259004)
Supplement: S3 Table — (DOCX) [file pone.0259004.s009.docx]

Supplementary Table 3: Jaccard similarity coefficient matrix comparing 20 different clusters

|  | cluster 1 | cluster 2 | cluster 3 | cluster 4 | cluster 5 | cluster 6 | cluster 7 | cluster 8 | cluster 9 | cluster 10 | cluster 11 | cluster 12 | cluster 13 | cluster 14 | cluster 15 | cluster 16 | cluster 17 | cluster 18 | cluster 19 | cluster 20 |
| --- | --- | --- | --- | --- | --- | --- | --- | --- | --- | --- | --- | --- | --- | --- | --- | --- | --- | --- | --- | --- |
| cluster 1 | 1 |  |  |  |  |  |  |  |  |  |  |  |  |  |  |  |  |  |  |  |
| cluster 2 | 0.11 | 1 |  |  |  |  |  |  |  |  |  |  |  |  |  |  |  |  |  |  |
| cluster 3 | 0.10 | 0.05 | 1 |  |  |  |  |  |  |  |  |  |  |  |  |  |  |  |  |  |
| cluster 4 | 0.03 | 0.01 | 0.04 | 1 |  |  |  |  |  |  |  |  |  |  |  |  |  |  |  |  |
| cluster 5 | 0.02 | 0 | 0.04 | 0.15 | 1 |  |  |  |  |  |  |  |  |  |  |  |  |  |  |  |
| cluster 6 | 0.03 | 0 | 0.02 | 0.09 | 0.33 | 1 |  |  |  |  |  |  |  |  |  |  |  |  |  |  |
| cluster 7 | 0.02 | 0.04 | 0.02 | 0.25 | 0.04 | 0.04 | 1 |  |  |  |  |  |  |  |  |  |  |  |  |  |
| cluster 8 | 0.05 | 0.03 | 0.03 | 0.11 | 0.04 | 0.06 | 0.07 | 1 |  |  |  |  |  |  |  |  |  |  |  |  |
| cluster 9 | 0.07 | 0.04 | 0.04 | 0.11 | 0.04 | 0.04 | 0.14 | 0.29 | 1 |  |  |  |  |  |  |  |  |  |  |  |
| cluster 10 | 0.04 | 0 | 0.02 | 0.13 | 0.28 | 0.38 | 0.03 | 0.08 | 0.05 | 1 |  |  |  |  |  |  |  |  |  |  |
| cluster 11 | 0.03 | 0 | 0.2 | 0.03 | 0 | 0 | 0 | 0.06 | 0.02 | 0 | 1 |  |  |  |  |  |  |  |  |  |
| cluster 12 | 0.03 | 0.08 | 0.01 | 0.03 | 0.04 | 0.08 | 0.01 | 0.06 | 0.01 | 0.10 | 0 | 1 |  |  |  |  |  |  |  |  |
| cluster 13 | 0.09 | 0.23 | 0.09 | 0.05 | 0.08 | 0.07 | 0.05 | 0.06 | 0.08 | 0.07 | 0 | 0.01 | 1 |  |  |  |  |  |  |  |
| cluster 14 | 0.06 | 0.03 | 0.04 | 0.14 | 0.18 | 0.18 | 0.03 | 0.08 | 0.05 | 0.16 | 0.01 | 0.06 | 0.13 | 1 |  |  |  |  |  |  |
| cluster 15 | 0.06 | 0.04 | 0.23 | 0.07 | 0.02 | 0.04 | 0 | 0.03 | 0.05 | 0.02 | 0.14 | 0.01 | 0.04 | 0.02 | 1 |  |  |  |  |  |
| cluster 16 | 0.07 | 0.05 | 0.14 | 0.05 | 0.04 | 0.05 | 0.02 | 0.07 | 0.06 | 0.04 | 0.1 | 0.03 | 0.13 | 0.12 | 0.06 | 1 |  |  |  |  |
| cluster 17 | 0 | 0 | 0 | 0.03 | 0.06 | 0.07 | 0 | 0.02 | 0 | 0.05 | 0 | 0.12 | 0 | 0.02 | 0 | 0 | 1 |  |  |  |
| cluster 18 | 0.03 | 0.06 | 0.05 | 0.03 | 0 | 0 | 0.02 | 0.03 | 0.02 | 0 | 0 | 0.09 | 0.05 | 0.06 | 0 | 0.08 | 0 | 1 |  |  |
| cluster 19 | 0.14 | 0.07 | 0.10 | 0.10 | 0.07 | 0.09 | 0.03 | 0.08 | 0.08 | 0.06 | 0.06 | 0.06 | 0.08 | 0.17 | 0.09 | 0.20 | 0.02 | 0.08 | 1 |  |
| cluster 20 | 0.10 | 0.16 | 0.05 | 0.04 | 0.03 | 0.04 | 0.03 | 0.04 | 0.03 | 0.04 | 0.01 | 0.2 | 0.06 | 0.11 | 0.02 | 0.09 | 0.01 | 0.15 | 0.13 | 1 |
