# Supplementary material for: Global biogeography of living brachiopods: Bioregionalization patterns and possible controls
Source: PLoS One. 2021 Nov 8;16(11):e0259004. doi: 10.1371/journal.pone.0259004 (PMC8575269; doi:10.1371/journal.pone.0259004)
Supplement: S4 Table — (DOCX) [file pone.0259004.s010.docx]

Supplementary Table 4: Jaccard similarity coefficient matrix comparing seven different Goups

|  | Group A | Group B | Group C | Group D | Group E | Group F | Group G |
| --- | --- | --- | --- | --- | --- | --- | --- |
| Group A | 1.000 |  |  |  |  |  |  |
| Group B | 0.170 | 1.000 |  |  |  |  |  |
| Group C | 0.053 | 0.086 | 1.000 |  |  |  |  |
| Group D | 0.129 | 0.087 | 0.182 | 1.000 |  |  |  |
| Group E | 0.095 | 0.063 | 0.082 | 0.158 | 1.000 |  |  |
| Group F | 0.110 | 0.073 | 0.049 | 0.131 | 0.164 | 1.000 |  |
| Group G | 0.022 | 0.026 | 0.000 | 0.014 | 0.000 | 0.050 | 1.000 |
